# New Phenylglycinamide Derivatives with Hybrid Structure as Candidates for New Broad-Spectrum Anticonvulsants

**DOI:** 10.3390/cells11121862

**Published:** 2022-06-07

**Authors:** Marcin Jakubiec, Michał Abram, Mirosław Zagaja, Marta Andres-Mach, Aleksandra Szewczyk, Gniewomir Latacz, Bartłomiej Szulczyk, Katarzyna Socała, Dorota Nieoczym, Piotr Wlaź, Cameron S. Metcalf, Karen Wilcox, Rafał M. Kamiński, Krzysztof Kamiński

**Affiliations:** 1 Department of Medicinal Chemistry, Faculty of Pharmacy, Jagiellonian University Medical College, Medyczna 9, 30-688 Krakow, Poland; marcin.jakubiec@doctoral.uj.edu.pl (M.J.); michal.abram@uj.edu.pl (M.A.); rafal1.kaminski@uj.edu.pl (R.M.K.); 2 Isobolographic Analysis Laboratory, Institute of Rural Health, Jaczewskiego 2, 20-950 Lublin, Poland; zagaja.miroslaw@imw.lublin.pl (M.Z.); andres.marta@imw.lublin.pl (M.A.-M.); szewczyk.aleksandra@imw.lublin.pl (A.S.); 3 Department of Technology and Biotechnology of Drugs, Faculty of Pharmacy, Jagiellonian University Medical College, Medyczna 9, 30-688 Krakow, Poland; gniewomir.latacz@uj.edu.pl; 4 Department of Pharmacodynamics, Centre for Preclinical Research and Technology, Medical University of Warsaw, Banacha 1B, 02-097 Warsaw, Poland; bartlomiej.szulczyk@wum.edu.pl; 5 Department of Animal Physiology and Pharmacology, Institute of Biological Sciences, Faculty of Biology and Biotechnology, Maria Curie-Skłodowska University, Akademicka 19, 20-033 Lublin, Poland; katarzyna.socala@mail.umcs.pl (K.S.); dorota.nieoczym@mail.umcs.pl (D.N.); piotr.wlaz@mail.umcs.pl (P.W.); 6 Department of Pharmacology and Toxicology, University of Utah, Salt Lake City, UT 84112, USA; cameron.s.metcalf@utah.edu (C.S.M.); karen.wilcox@hsc.utah.edu (K.W.)

**Keywords:** hybrid molecules, multimodal/multi-target compounds, amino acid derivatives, antiseizure activity, in vitro binding/functional studies, ADME-Tox properties

## Abstract

In the present study, a focused combinatorial chemistry approach was applied to merge structural fragments of well-known TRPV1 antagonists with a potent anticonvulsant lead compound, **KA-104**, that was previously discovered by our group. Consequently, a series of 22 original compounds has been designed, synthesized, and characterized in the in vivo and in vitro assays. The obtained compounds showed robust in vivo antiseizure activity in the maximal electroshock (MES) test and in the 6 Hz seizure model (using both 32 and 44 mA current intensities). The most potent compounds **53** and **60** displayed the following pharmacological profile: ED_50_ = 89.7 mg/kg (MES), ED_50_ = 29.9 mg/kg (6 Hz, 32 mA), ED_50_ = 68.0 mg/kg (6 Hz, 44 mA), and ED_50_ = 73.6 mg/kg (MES), ED_50_ = 24.6 mg/kg (6 Hz, 32 mA), and ED_50_ = 56.3 mg/kg (6 Hz, 44 mA), respectively. Additionally, **53** and **60** were effective in the *iv*PTZ seizure threshold and had no influence on the grip strength and body temperature in mice. The in vitro binding and functional assays indicated a multimodal mechanism of action for **53** and **60**. These molecules, beyond TRPV1 antagonism, inhibited calcium currents and fast sodium currents in patch-clamp assays. Further studies proved beneficial in vitro ADME-Tox properties for **53** and **60** (i.e., high metabolic stability, weak influence on CYPs, no neurotoxicity, etc.). Overall, **53** and **60** seem to be interesting candidates for future preclinical development in epilepsy and pain indications due to their interaction with the TRPV1 channel.

## 1. Introduction

Epilepsy is recognized as one of the most common neurological disorders just after stroke [1]. It is characterized by a multifactorial pathogenesis, which often substantially limits the clinical efficacy of currently available antiseizure drugs (ASDs). Notably, despite significant advances in epilepsy research and approval of several new ASDs, nearly 30% of patients do not respond to current therapy and have drug-resistant epilepsy (DRE) [2]. Therefore, DRE is a serious clinical condition that puts the patient at risk of sudden unexpected death in epilepsy (SUDEP), as well as psychiatric, psychosocial, and medical complications, having a profound influence on the overall quality of life. 

The current strategy for the effective management of multifactorial diseases assumes the application of drug combinations or multimodal (multi-target/multi-functional) drugs, acting on several complementary molecular mechanisms [3,4]. Such multi-target molecules are designed most often as hybrid or chimeric approaches that integrate multiple pharmacophores into a single molecule in order to provide broader and hopefully synergic mechanism of action. Notably, the transition from the single-target to the multi-target concept is observed predominantly in the case of neurodegenerative diseases (i.e., Alzheimer’s and Parkinson’s), depression, diabetes, metabolic and inflammatory diseases, cancer, as well as other neurological disorders such as epilepsy and neuropathic pain [5,6,7,8,9,10,11,12]. Importantly, the current literature data indicate higher clinical efficacy of ASDs with a multimodal mechanism of action vs. drugs that act on a single target, such as ASD with superior efficacy valproic acid (VPA) [13]. It should be also stressed that combining different molecular mechanisms is potentially beneficial in the treatment of diseases with a high risk of drug resistance, including epilepsy, as well as infectious diseases that are caused by different pathogens (i.e., bacteria, fungi, etc.) [14,15,16]. Finally, the use of multi-target drugs may reduce the overall drug load (especially during combination therapy), and as a result, may reduce the risk of potential drug–drug interactions (DDIs) and multiple side effects leading to a better therapy compliance.

The TRPV1 (transient receptor potential cation channel, subfamily V member 1, also known as the vanilloid receptor 1) is a nonselective cation channel that is permeable principally for calcium ions. The TRPV1, cloned in 1997 by David Julius and colleagues, was a breakthrough in sensory biology and pain research [17]. TRPV1 channels are expressed predominantly in the afferent sensory neurons and are particularly abundant in C and Aδ nociceptive fibers, where they play a key role in the detection of noxious painful stimuli [18]. For many years TRPV1 has been recognized as one of the most promising and widely explored molecular targets for new and effective analgesics [19,20]. The results of hitherto studies (including clinical trials) have shown that TRPV1 antagonists may be useful in the treatment of pain of various origins, e.g., inflammatory, neuropathic, postoperative, visceral, cancer, and migraine pain [21,22,23]. Interestingly enough, recent studies show that TRPV1 channels are also located in the central nervous system (CNS) (i.e., hippocampus, cortex), and their activation may play an essential role in the induction of seizures and propagation of epileptogenesis [24,25,26]. Thus, it is postulated that TRPV1 antagonists penetrating the blood-brain barrier may be promising ASD candidates, which adds to their potent central and peripheral antinociceptive properties. It should be emphasized herein that cannabidiol (CBD) which is one of the newest ASDs effective in DRE (i.e., Dravet and Lennox-Gastaut syndromes in children), has a multi-target mechanism of action and causes desensitization of the TRPV1, besides the inhibition of sodium and calcium conductance [27,28]. Therefore, it is suggested that a multimodal mechanism of action underlines a broad spectrum of antiseizure activity of CBD that was demonstrated in preclinical studies [29].

**Figure 1:**
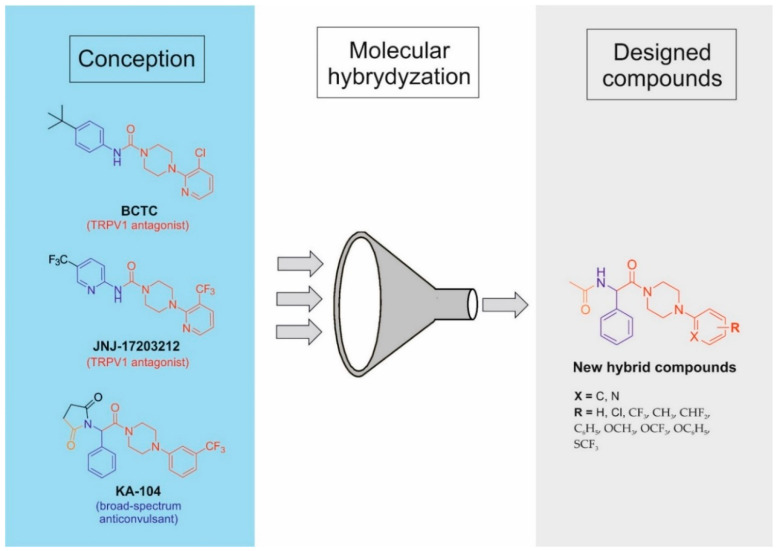
Design strategy and general structure of new hybrid molecules.

Following the concept of multi-target strategy, we have recently discovered several series of hybrid compounds that are based on the pyrrolidine-2,5-dione scaffold [30,31,32,33]. These hybrid molecules proved to possess potent and broad-spectrum anticonvulsant activity in the maximal electroshock (MES), the 6 Hz (32/44 mA), and the pentylenetetrazole-induced (*sc*PTZ) seizure models in mice. The most promising antiseizure properties and a favorable safety profile were reported, among others, for compound **KA-104** (Figure 1) [32,34].

In addition to its potent anticonvulsant efficacy, **KA-104** effectively decreases nociceptive responses in formalin-induced tonic pain, capsaicin-induced neurogenic pain, and notably, and oxaliplatin-induced neuropathic pain in mice [32,34]. **KA-104** displays a multi-target mechanism of action including TRPV1 channel antagonism as well as a blockade of voltage-gated sodium channels (VGSCs) and Cav_1.2_ (L-type) calcium channels. Importantly, the VGSCs play a fundamental role in establishing and regulating the excitability of CNS neurons as well as conductance of the pain stimuli [35,36]. Additionally, Cav_1.2_ channels that are located in the dorsal horn neurons are known to be crucial for the long-term sensitization of pain [37,38]. Notably, the Cav_1.2_ voltage-gated calcium channels are also widely distributed throughout the CNS and participate in neuronal firing and gene expression regulation [39]. Thus, Cav_1.2_ channels play a key role in the pathophysiology of several neurological disorders, i.e., epilepsy, Parkinson disease, and pain [35,40,41]. 

In order to improve antiseizure and/or potentially antinociceptive activity, we developed herein a new series of phenylglycinamide derivatives by application of the focused combinatorial chemistry approach. In consequence, these novel compounds were designed as hybrids that integrate structural fragments of chemical prototype–**KA-104** and acyclic selective TRPV1 antagonists such as **BCTC** and **JNJ-17203212** with proven analgesic activity in preclinical studies (Figure 1) [42,43,44]. Furthermore, the hybrids that are described herein may be also recognized as close analogs of compound **KA-104** with degraded succinimide moiety. The chemical modification of the phenylpiperazine moiety was focused mainly on the introduction of electron-withdrawing substituents that were favorable for the antiseizure effect, as it was described for succinimide derivatives previously [32,33,45]. Therefore, we hypothesize that such molecules may be characterized by a multi-targeted mechanism of action, namely, they may provide an interaction with TRPV1, Na_v_x, and Ca_v1.2_ channels, and thus may provide potent anticonvulsant and probably analgesic activity.

The current studies were designed as an integrated drug discovery approach consisting of design, synthesis, in vitro testing in binding/functional assays, and in vivo determination of anticonvulsant activity. In addition, we assessed several ADME-Tox properties that were crucial for early development of new drug candidates such as permeability, metabolic stability, hepatotoxicity, neurotoxicity, or influence on the function of several cytochrome P-450 isoforms (CYP3A4, CYP2D6, and CYP2C9).

## 2. Materials and Methods

### 2.1. Chemistry

All chemicals and solvents were purchased from commercial suppliers and were used without further purification. The melting points (mp.) were determined in open capillaries on a Büchi 353 melting point apparatus (Büchi Labortechnik, Flawil, Switzerland). TLC and the gradient UPLC chromatography were used to assess the purity and homogeneity of the compounds. TLC was carried out on silica gel 60 F_254_ pre-coated aluminum sheets (Macherey-Nagel, Düren, Germany), using the following developing systems: S_1_–DCM:MeOH (9:0.2; *v*/*v*), S_2_–DCM:MeOH (9:0.3; *v/v*), and S_3_–DCM:MeOH (9:0.5; *v*/*v*). Spots detection: UV light (λ = 254 nm). The UPLC and mass spectra (LC-MS) were obtained on a Waters ACQUITY™ TQD system (Waters, Milford, CT, USA) with the MS-TQ detector and UV-Vis-DAD eλ detector. The ACQUITY UPLC BEH C18, 1.7 μm (2.1 × 100 mm) column was used with the VanGuard Acquity UPLC BEH C18, 1.7 μm (2.1 × 5 mm) (Waters, Milford, CT, USA). Standard solutions (1 mg/mL) of each compound were prepared in analytical grade MeCN/water mixture (1:1; *v*/*v*). The conditions that were applied were as follows: eluent A (water/0.1% HCOOH), eluent B (MeCN/0.1% HCOOH), a flow rate of 0.3 mL/min, a gradient of 5–100% B over 10 min, and an injection volume of 10 μL. The UPLC retention times (*t*_R_) are given in minutes (min). The purity of target compounds was determined by the use of chromatographic UPLC method was ≥99%. Preparative column chromatography was performed using silica gel 60 (particle size 0.063–0.200; 70–230 Mesh ATM) that was purchased from Merck (Darmstadt, Germany). ^1^H NMR and ^13^C NMR spectra were obtained in a JEOL-500 spectrometer (JEOL USA, Inc. MA, USA) in CDCl_3_ operating at 500 MHz (^1^H NMR) and 126 MHz (^13^C NMR). Chemical shifts are reported in δ values (ppm) relative to TMS δ = 0 (^1^H), as an internal standard. The *J* values are expressed in Hertz (Hz). Signal multiplicities are represented by the following abbreviations: s (singlet), br. s. (broad singlet), d (doublet), dd (double doublet), t (triplet), td (triple doublet), q (quartet), and m (multiplet).

#### 2.1.1. General Method for the Preparation of Intermediates **A1–A12**

**Scheme 1:**
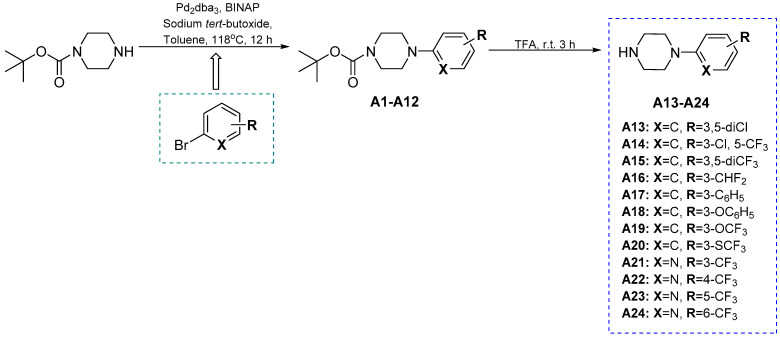
Synthesis of starting and non-commercial 4-arylpiperazine derivatives **A13**–**A24**.

The starting (non-commercial) Boc-derivatives of 4-aryl-piperazine were obtained in *N*-arylation reaction according to Scheme 1 (see Section 3 Results and Discussion). The appropriate aryl bromide (10 mmol, 1 eq), Pd_2_dba_3_ (0.37 g, 0.4 mmol, 0.04 eq), BINAP (0.37 g, 0.59 mmol, 0.06 eq), sodium *tert*-butoxide (1.35 g, 14 mmol, 1.4 eq), and Boc-piperazine (3.74 g, 20 mmol, 2 eq) were suspended in an inert gas (nitrogen) atmosphere in 50 mL of dry toluene. Next, the reaction mixture was refluxed for 12 h, subsequently cooled, and filtered through Celite 545 Merck (Darmstadt, Germany), and then concentrated under reduced pressure. The Boc-protected amines **A1**−**A12** were purified by column chromatography using the following developing systems: S_1_ (**A1**–**A4**, **A7**–**A12**) or S_2_ (**A5**, **A6**).

***Tert*-butyl 4-(3,5-dichlorophenyl)piperazine-1-carboxylate (A1)**. Yellow oil, yield 65% (2.15 g); TLC: *R*_f_ = 0.83 (S_1_); UPLC (purity > 99%): *t*_R_ = 9.32 min. LC-MS (ESI): *m/z* calcd for C_15_H_20_Cl_2_N_2_O_2_ (M+H)^+^ 331.09, found 331.1.

***Tert*-butyl 4-(3-chloro-5-(trifluoromethyl)phenyl)piperazine-1-carboxylate (A2)**. Yellow oil, yield 72% (2.63 g); TLC: *R*_f_ = 0.84 (S_1_); UPLC (purity 93.7%): *t*_R_ = 9.27 min. LC-MS (ESI): *m/z* calcd for C_16_H_20_ClF_3_N_2_O_2_ (M+H)^+^ 365.12, found 365.2.

***Tert*-butyl 4-(3,5-bis(trifluoromethyl)phenyl)piperazine-1-carboxylate (A3)**. Yellow oil, yield 63% (2.51 g); TLC: *R*_f_ = 0.89 (S_1_); UPLC (purity > 99%): *t*_R_ = 9.34 min. LC-MS (ESI): *m/z* calcd for C_17_H_20_F_6_N_2_O_2_ (M+H)^+^ 399.14, found 399.5.

***Tert*-butyl 4-(3-(difluoromethyl)phenyl)piperazine-1-carboxylate (A4)**. Yellow oil, yield 72% (2.25 g); TLC: *R*_f_ = 0.84 (S_1_); UPLC (purity > 99%): *t*_R_ = 7.83 min. LC-MS (ESI): *m/z* calcd for C_16_H_22_F_2_N_2_O_2_ (M+H)^+^ 313.16, found 313.4.

***Tert*-butyl 4-([1,1’-biphenyl]-3-yl)piperazine-1-carboxylate (A5)**. Yellow oil, yield 64% (2.17 g); TLC: *R*_f_ = 0.70 (S_2_); UPLC (purity > 99%): *t*_R_ = 8.95 min. LC-MS (ESI): *m/z* calcd for C_21_H_26_N_2_O_2_ (M+H)^+^ 339.23, found 339.2.

***Tert*-butyl 4-(3-phenoxyphenyl)piperazine-1-carboxylate (A6)**. Yellow oil, yield 67% (2.37 g); TLC: *R*_f_ = 0.77 (S_2_); UPLC (purity 92.3%): *t*_R_ = 9.07 min. LC-MS (ESI): *m/z* calcd for C_21_H_26_N_2_O_3_ (M+H)^+^ 355.20, found 355.3.

***Tert*-butyl 4-(3-(trifluoromethoxy)phenyl)piperazine-1-carboxylate (A7)**. Yellow oil, yield 68% (2.36 g); TLC: *R*_f_ = 0.72 (S_1_); UPLC (purity > 99%): *t*_R_ = 8.73 min. LC-MS (ESI): *m/z* calcd for C_16_H_21_F_3_N_2_O_3_ (M+H)^+^ 347.16, found 347.2.

***Tert*-butyl 4-(3-((trifluoromethyl)thio)phenyl)piperazine-1-carboxylate (A8)**. Yellow oil, yield 71% (2.57 g); TLC: *R*_f_ = 0.74 (S_1_); UPLC (purity 93.8%): *t*_R_ = 9.14 min. LC-MS (ESI): *m/z* calcd for C_16_H_21_F_3_N_2_O_2_S (M+H)^+^ 363.13, found 363.2.

***Tert*-butyl 4-(3-(trifluoromethyl)pyridin-2-yl)piperazine-1-carboxylate (A9)**. Yellow oil, yield 67% (2.2 g); TLC: *R*_f_ = 0.82 (S_1_); UPLC (purity 91.3%): *t*_R_ = 8.11 min. LC-MS (ESI): *m/z* calcd for C_15_H_20_F_3_N_3_O_2_ (M+H)^+^ 332.15, found 332.3.

***Tert*-butyl 4-(4-(trifluoromethyl)pyridin-2-yl)piperazine-1-carboxylate (A10)**. Yellow oil, yield 69% (2.29 g); TLC: *R*_f_ = 0.81 (S_1_); UPLC (purity 92.4%): *t*_R_ = 8.02 min. LC-MS (ESI): *m/z* calcd for C_15_H_20_F_3_N_3_O_2_ (M+H)^+^ 332.15, found 332.4.

***Tert*-butyl 4-(5-(trifluoromethyl)pyridin-2-yl)piperazine-1-carboxylate (A11)**. Yellow oil, yield 63% (2.09); TLC: *R*_f_ = 0.8 (S_1_); UPLC (purity 84.7%): *t*_R_ = 8.11 min. LC-MS (ESI): *m/z* calcd for C_15_H_20_F_3_N_3_O_2_ (M+H)^+^ 332.15, found 332.3.

***Tert*-butyl 4-(6-(trifluoromethyl)pyridin-2-yl)piperazine-1-carboxylate (A12)**. Yellow oil, yield 66% (2.19 g); TLC: *R*_f_ = 0.82 (S_1_); UPLC (purity 88.9%): *t*_R_ = 8.05 min. LC-MS (ESI): *m/z* calcd for C_15_H_20_F_3_N_3_O_2_ (M+H)^+^ 332.15, found 332.1.

#### 2.1.2. General Method for the Preparation of Starting Amines **A13**–**24**

The solution of **A1**–**A12** (5 mmol, 1 eq) in DCM (5 mL) was treated with TFA (1.71 g, 15 mmol, 3 eq) and stirred at room temperature for 3 h. Afterwards, the organic solvents were evaporated to dryness. The resulting oil residue was dissolved in water (20 mL), and then 25% ammonium hydroxide was carefully added until pH 8. The aqueous layer was extracted with DCM (3 × 20 mL), dried over Na_2_SO_4_, and concentrated to give **A13**–**A24** as yellow or bronze oils. Non-commercial amines **A13**–**A24** were used as substrates for the next reactions without purification. The synthetic pathway is shown in Scheme 1.

**1-(3,5-Dichlorophenyl)piperazine (A13)**. Yellow oil, yield 97% (1.21 g); TLC: *R*_f_ = 0.48 (S_3_); UPLC (purity > 99%): *t*_R_ = 3.99 min. LC-MS (ESI): *m/z* calcd for C_10_H_12_Cl_2_N_2_ (M+H)^+^ 231.04, found 231.0. ^1^H NMR (500 MHz, CDCl_3_) δ 2.02–2.18 (m, 1 H, piperazine) 2.97–3.00 (m, 4 H, piperazine), 3.11–3.14 (m, 4 H, piperazine), 6.72 (s, 2 H, ArH), 6.77 (s, 1 H, ArH).

**1-(3-Chloro-5-(trifluoromethyl)phenyl)piperazine (A14)**. Yellow oil, yield 97% (1.2 g); TLC: *R*_f_ = 0.49 (S_3_); UPLC (purity 95.2%): *t*_R_ = 4.53 min. LC-MS (ESI): *m/z* calcd for C_11_H_12_ClF_3_N_2_ (M+H)^+^ 265.06, found 265.2. ^1^H NMR (500 MHz, CDCl_3_) δ 2.64–2.73 (m, 2 H, piperazine), 3.01–3.06 (m, 3 H, piperazine), 3.20–3.26 (m, 4 H, piperazine), 6.97 (d, *J* = 9.7 Hz, 2 H, ArH), 7.00–7.03 (m, 1 H, ArH).

**1-(3,5-Bis(trifluoromethyl)phenyl)piperazine (A15)**. Yellow oil, yield 96% (1.43 g); TLC: *R*_f_ = 0.52 (S_3_); UPLC (purity > 99%): *t*_R_ = 4.80 min. LC-MS (ESI): *m/z* calcd for C_12_H_12_F_6_N_2_ (M+H)^+^ 299.09, found 299.3. ^1^H NMR (500 MHz, CDCl_3_) δ 2.63 (br. s, 1 H, piperazine), 3.03–3.05 (m, 4 H, piperazine), 3.23–3.25 (m, 4 H, piperazine), 7.22 (s, 2 H, ArH), 7.25–7.26 (m, 1 H, ArH).

**1-(3-(Difluoromethyl)phenyl)piperazine (A16)**. Yellow oil, yield 98% (1.04 g); TLC: *R*_f_ = 0.49 (S_3_); UPLC (purity > 99%): *t*_R_ = 3.01 min. LC-MS (ESI): *m/z* calcd for C_11_H_14_F_2_N_2_ (M+H)^+^ 213.11, found 213.0. ^1^H NMR (500 MHz, CDCl_3_) δ 2.63–2.66 (m, 1 H, piperazine), 3.15–3.18 (m, 4 H, piperazine), 3.27–3.30 (m, 4 H, piperazine), 6.40–6.55 (m, 1 H, CHF_2_), 6.60–6.73 (m, 1 H, ArH), 6.96–7.03 (m, 1 H, ArH), 7.05–7.11 (m, 1 H ArH), 7.15–7.23 (m, 1 H, ArH).

**1-([1,1’-Biphenyl]-3-yl)piperazine (A17)**. Yellow oil, yield 95% (1.13 g); TLC: *R*_f_ = 0.52 (S_3_); UPLC (purity > 99%): *t*_R_ = 4.39 min. LC-MS (ESI): *m/z* calcd for C_16_H_18_N_2_ (M+H)^+^ 239.15, found 239.2. ^1^H NMR (300 MHz, CDCl_3_) δ 1.15–1.28 (m, 1 H, piperazine), 3.14–3.76 (m, 8 H, piperazine), 6.65–7.73 (m, 9 H, ArH).

**1-(3-Phenoxyphenyl)piperazine (A18)**. Yellow oil, yield 97% (1.23 g); TLC: *R*_f_ = 0.45 (S_3_); UPLC (purity 93.6%): *t*_R_ = 4.61 min. LC-MS (ESI): *m/z* calcd for C_16_H_18_N_2_O (M+H)^+^ 255.15, found 255.3. ^1^H NMR (500 MHz, CDCl_3_) δ 2.59–2.69 (m, 1 H, piperazine) 3.00 (s, 1 H, piperazine) 3.14–3.22 (m, 4 H, piperazine) 3.27–3.34 (m, 3 H, piperazine) 6.41–6.72 (m, 3 H, ArH) 6.96–7.39 (m, 6 H, ArH).

**1-(3-(Trifluoromethoxy)phenyl)piperazine (A19)**. Yellow oil, yield 97% (1.20 g); TLC: *R*_f_ = 0.41 (S_3_); UPLC (purity 95.7%): *t*_R_ = 3.99 min. LC-MS (ESI): *m/z* calcd for C_11_H_13_F_3_N_2_O (M+H)^+^ 247.10, found 247.1. ^1^H NMR (300 MHz, CDCl_3_) δ 1.23 (s, 1 H, piperazine) 2.91–3.16 (m, 8 H, piperazine) 6.59–6.85 (m, 3 H, ArH), 7.19 (t, *J* = 8.3 Hz, 1 H, ArH).

**1-(3-((Trifluoromethyl)thio)phenyl)piperazine (A20)**. Yellow oil, yield 96% (1.25 g); TLC: *R*_f_ = 0.42 (S_3_); UPLC (purity 93.9%): *t*_R_ = 3.69 min. LC-MS (ESI): *m/z* calcd for C_11_H_13_F_3_N_2_S (M+H)^+^ 263.08, found 263.1. ^1^H NMR (500 MHz, CDCl_3_) δ 2.65–2.71 (m, 1 H, piperazine), 3.10–3.16 (m, 3 H, piperazine), 3.18–3.31 (m, 5 H, piperazine), 6.99–7.01 (m, 1 H, ArH), 7.09–7.16 (m, 2 H, ArH), 7.25–7.30 (m, 1 H, ArH).

**1-(3-(Trifluoromethyl)pyridin-2-yl)piperazine (A21)**. Bronze oil, yield 96% (1.11 g); TLC: *R*_f_ = 0.43 (S_3_); UPLC (purity 94.3%): *t*_R_ = 2.66 min. LC-MS (ESI): *m/z* calcd for C_10_H_12_F_3_N_2_ (M+H)^+^ 232.10, found 232.4. ^1^H NMR (500 MHz, CDCl_3_) δ 2.97–3.07 (m, 4 H, piperazine), 3.10–3.19 (m, 2 H, piperazine), 3.24–3.29 (m, 3 H, piperazine), 6.91–7.02 (m, 1 H, ArH), 7.79–7.87 (m, 1 H, ArH), 8.40 (dd, *J* = 4.8, 1.32 Hz, 1 H, ArH).

**1-(4-(Trifluoromethyl)pyridin-2-yl)piperazine (A22)**. Bronze oil, yield 97% (1.12 g); TLC: *R*_f_ = 0.42 (S_3_); UPLC (purity 92.4%): *t*_R_ = 2.89 min. LC-MS (ESI): *m/z* calcd for C_10_H_12_F_3_N_2_ (M+H)^+^ 232.10, found 232.3. ^1^H NMR (500 MHz, CDCl_3_) δ 3.54 (d, *J* = 5.9 Hz, 4 H, piperazine), 3.56–3.60 (m, 5 H, piperazine), 6.76–6.80 (m, 2 H, ArH), 8.29 (d, *J* = 5.1 Hz, 1 H, ArH).

**1-(5-(Trifluoromethyl)pyridin-2-yl)piperazine (A23)**. Bronze oil, yield 96% (1.11 g); TLC: *R*_f_ = 0.41 (S_3_); UPLC (purity 86.8%): *t*_R_ = 2.74 min. LC-MS (ESI): *m/z* calcd for C_10_H_12_F_3_N_2_ (M+H)^+^ 232.10, found 232.2. ^1^H NMR (500 MHz, CDCl_3_) δ 3.46–3.57 (m, 4 H, piperazine), 3.58–3.66 (m, 5 H, piperazine), 6.62 (d, *J* = 9.0 Hz, 1 H, ArH), 7.59–7.66 (m, 1 H, ArH), 8.39 (dd, *J* = 1.6, 0.9 Hz, 1 H, ArH).

**1-(6-(Trifluoromethyl)pyridin-2-yl)piperazine (A24)**. Bronze oil, yield 98% (1.13 g); TLC: *R*_f_ = 0.42 (S_3_); UPLC (purity 89.7%): *t*_R_ = 2.96 min. LC-MS (ESI): *m/z* calcd for C_10_H_12_F_3_N_2_ (M+H)^+^ 232.10, found 232.3. ^1^H NMR (500 MHz, CDCl_3_) δ 3.19–3.25 (m, 5 H, piperazine), 3.57–3.67 (m, 4 H, piperazine), 7.21–7.34 (m, 1 H), 7.40 (s, 1 H), 7.64–7.79 (m, 1 H).

#### 2.1.3. General Method for the Preparation of Intermediates **1**–**22**

**Scheme 2:**
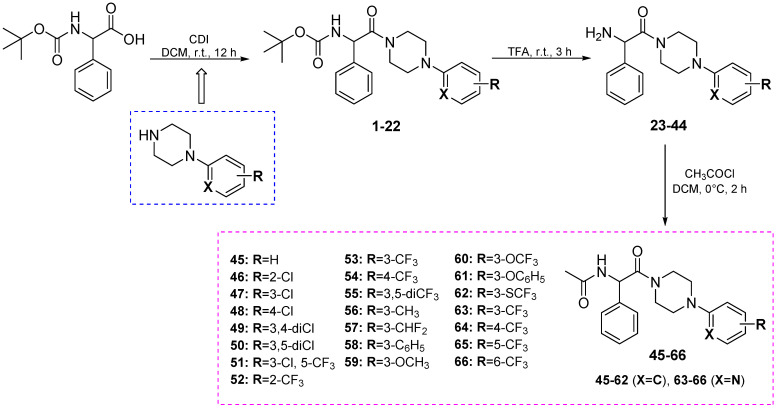
Synthesis of intermediates **1**–**44** and final compounds **45**–**66**.

Carbonyldiimidazole (CDI) (0.39 g, 2.4 mmol, 1.2 eq) was dissolved in DCM (10 mL). Afterward, this solution was added to Boc-phenylglicine (0.5 g, 2 mmol, 1 eq) dissolved in 10 mL of DCM (while stirring). After 0.5 h, the respective piperazine derivatives (2 mmol, 1 eq) that were dissolved in 5 mL of DCM was added in drops. The mixture was stirred for approximately 2 h at room temperature and evaporated to dryness. The column chromatography was applied for the purification of crude products using mixture S_2_ as a developing system. Compounds **1**–**22** were obtained as light oils followed by a concentration of organic solvents under reduced pressure. The synthetic pathway is shown in Scheme 2 (see Section 3 Results and Discussion).

***Tert*-butyl (2-oxo-1-phenyl-2-(4-phenylpiperazin-1-yl)ethyl)carbamate (1)**. Light yellow oil, yield 87% (0.72 g); TLC: *R*_f_ = 0.70 (S_1_); UPLC (purity > 99%): *t*_R_ = 7.78 min. LC-MS (ESI): *m/z* calcd for C_23_H_29_N_3_O_3_ (M+H)^+^ 396.22, found 396.3.

***Tert*-butyl (2-(4-(2-chlorophenyl)piperazin-1-yl)-2-oxo-1-phenylethyl)carbamate (2)**. Light yellow oil, yield 81% (0,69 g); TLC: *R*_f_ = 0.78 (S_2_); UPLC (purity > 99%): *t*_R_ = 8.52 min. LC-MS (ESI): *m/z* calcd for C_23_H_28_N_3_O_3_Cl (M+H)^+^ 430.19, found 430.4.

***Tert*-butyl (2-(4-(3-chlorophenyl)piperazin-1-yl)-2-oxo-1-phenylethyl)carbamate (3)**. Light yellow oil, yield 82% b (0.7 g); TLC: *R*_f_ = 0.77 (S_2_); UPLC (purity > 99%): *t*_R_ = 8.38 min. LC-MS (ESI): *m/z* calcd for C_23_H_28_N_3_O_3_Cl (M+H)^+^ 430.19, found 430.4.

***Tert*-butyl (2-(4-(4-chlorophenyl)piperazin-1-yl)-2-oxo-1-phenylethyl)carbamate (4)**. Light yellow oil, yield 85% (0.73 g); TLC: *R*_f_ = 0.78 (S_2_); UPLC (purity > 99%): *t*_R_ = 8.32 min. LC-MS (ESI): *m/z* calcd for C_23_H_28_N_3_O_3_Cl (M+H)^+^ 430.19, found 430.3.

***Tert*-butyl (2-(4-(3,4-dichlorophenyl)piperazin-1-yl)-2-oxo-1-phenylethyl)carbamate (5)**. Light yellow oil, yield 83% (0.77 g); TLC: *R*_f_ = 0.83 (S_2_); UPLC (purity > 99%): *t*_R_ = 9.12 min. LC-MS (ESI): *m/z* calcd for C_23_H_27_N_3_O_3_Cl_2_ (M+H)^+^ 464.15 found 464.5. 

***Tert*-butyl (2-(4-(3,5-dichlorophenyl)piperazin-1-yl)-2-oxo-1-phenylethyl)carbamate (6)**. Light yellow oil, yield 80% (0.74 g); TLC: *R*_f_ = 0.81 (S_2_); UPLC (purity > 99%): *t*_R_ = 9.03 min. LC-MS (ESI): *m/z* calcd for C_23_H_27_N_3_O_3_Cl_2_ (M+H)^+^ 464.15 found 464.6. 

***Tert*-butyl (2-(4-(3-chloro-5-(trifluoromethyl)phenyl)piperazin-1-yl)-2-oxo-1-phenyl ethyl) carbamate (7)**. Light yellow oil, yield 81% (0.8 g); TLC: *R*_f_ = 0.81 (S_2_); UPLC (purity > 99%): *t*_R_ = 8.92 min. LC-MS (ESI): *m/z* calcd for C_24_H_27_N_3_O_3_ClF_3_ (M+H)^+^ 498.17 found 498.4.

***Tert*-butyl (2-oxo-1-phenyl-2-(4-(2-(trifluoromethyl)phenyl)piperazin-1-yl)ethyl) carbamate (8)**. Light yellow oil, yield 87% (0.8 g); TLC: *R*_f_ = 0.81 (S_2_); UPLC (purity > 99%): *t*_R_ = 8.71 min. LC-MS (ESI): *m/z* calcd for C_24_H_28_N_3_O_3_F_3_ (M+H)^+^ 464.21, found 464.2.

***Tert*-butyl (2-oxo-1-phenyl-2-(4-(3-(trifluoromethyl)phenyl)piperazin-1-yl)ethyl) carbamate (9)**. Light yellow oil, yield 83% (0.77 g); TLC: *R*_f_ = 0.79 (S_2_); UPLC (purity > 99%): *t*_R_ = 8.47 min. LC-MS (ESI): *m/z* calcd for C_24_H_28_N_3_O_3_F_3_ (M+H)^+^ 464.21, found 464.3.

***Tert*-butyl (2-oxo-1-phenyl-2-(4-(4-(trifluoromethyl)phenyl)piperazin-1-yl)ethyl) carbamate (10)**. Light yellow oil, yield 86% (0.79 g); TLC: *R*_f_ = 0.81 (S_2_); UPLC (purity > 99%): *t*_R_ = 8.43 min. LC-MS (ESI): *m/z* calcd for C_24_H_28_N_3_O_3_F_3_ (M+H)^+^ 464.21, found 464.4.

***Tert*-butyl (2-(4-(3,5-bis(trifluoromethyl)phenyl)piperazin-1-yl)-2-oxo-1-phenylethyl) carbamate (11)**. Light yellow oil, yield 80% (0.85 g); TLC: *R*_f_ = 0.85 (S_2_); UPLC (purity > 99%): *t*_R_ = 9.17 min. LC-MS (ESI): *m/z* calcd for C_25_H_27_N_3_O_3_F_6_ (M+H)^+^ 532.20 found 532.1. 

***Tert*-butyl (2-oxo-1-phenyl-2-(4-(m-tolyl)piperazin-1-yl)ethyl)carbamate (12)**. Light yellow oil, yield 88% (0.72 g); TLC: *R*_f_ = 0.68 (S_2_); UPLC (purity > 99%): *t*_R_ = 8.13 min. LC-MS (ESI): *m/z* calcd for C_24_H_31_N_3_O_3_ (M+H)^+^ 410.24 found 410.3. 

***Tert*-butyl (2-(4-(3-(difluoromethyl)phenyl)piperazin-1-yl)-2-oxo-1-phenylethyl) carbamate (13)**. Light yellow oil, yield 85% (0.75 g); TLC: *R*_f_ = 0.78 (S_2_); UPLC (purity > 99%): *t*_R_ = 7.82 min. LC-MS (ESI): *m/z* calcd for C_24_H_29_N_3_O_3_F_2_ (M+H)^+^ 446.22 found 446.3. 

***Tert*-butyl (2-(4-([1,1’-biphenyl]-3-yl)piperazin-1-yl)-2-oxo-1-phenylethyl)carbamate (14)**. Light yellow oil, yield 83% (0.78 g); TLC: *R*_f_ = 0.75 (S_2_); UPLC (purity > 99%): *t*_R_ = 8.85 min. LC-MS (ESI): *m/z* calcd for C_29_H_33_N_3_O_3_ (M+H)^+^ 472.26 found 472.5. 

***Tert*-butyl (2-(4-(3-methoxyphenyl)piperazin-1-yl)-2-oxo-1-phenylethyl)carbamate (15)**. Light yellow oil, yield 82% (0.69 g); TLC: *R*_f_ = 0.65 (S_2_); UPLC (purity > 99%): *t*_R_ = 7.64 min. LC-MS (ESI): *m/z* calcd for C_24_H_31_N_3_O_4_ (M+H)^+^ 426.23 found 426.3. 

***Tert*-butyl (2-oxo-1-phenyl-2-(4-(3-(trifluoromethoxy)phenyl)piperazin-1-yl)ethyl) carbamate (16)**. Light yellow oil, yield 83% (0.78 g); TLC: *R*_f_ = 0.81 (S_2_); UPLC (purity > 99%): *t*_R_ = 8.90 min. LC-MS (ESI): *m/z* calcd for C_24_H_28_N_3_O_4_ F_3_ (M+H)^+^ 481.21 found 481.5.

***Tert*-butyl (2-oxo-2-(4-(3-phenoxyphenyl)piperazin-1-yl)-1-phenylethyl)carbamate (17)**. Light yellow oil, yield 82% (0.8 g); TLC: *R*_f_ = 0.79 (S_2_); UPLC (purity > 99%): *t*_R_ = 8.85 min. LC-MS (ESI): *m/z* calcd for C_29_H_33_N_3_O_4_ (M+H)^+^ 488.25 found 488.3. 

***Tert*-butyl (2-oxo-1-phenyl-2-(4-(3-((trifluoromethyl)thio)phenyl)piperazin-1-yl)ethyl) carbamate (18)**. Light yellow oil, yield 82% (0.81 g); TLC: *R*_f_ = 0.82 (S_2_); UPLC (purity > 99%): *t*_R_ = 8.95 min. LC-MS (ESI): *m/z* calcd for C_24_H_28_N_3_O_3_SF_3_ (M+H)^+^ 496.18 found 496.2. 

***Tert*-butyl (2-oxo-1-phenyl-2-(4-(3-(trifluoromethyl)pyridin-2-yl)piperazin-1-yl)ethyl) carbamate (19)**. Light bronze oil, yield 82% (0.76 g); TLC: *R*_f_ = 0.78 (S_2_); UPLC (purity > 99%): *t*_R_ = 8.12 min. LC-MS (ESI): *m/z* calcd for C_23_H_27_N_4_O_3_F_3_ (M+H)^+^ 465.21 found 465.4.

***Tert*-butyl (2-oxo-1-phenyl-2-(4-(4-(trifluoromethyl)pyridin-2-yl)piperazin-1-yl)ethyl) carbamate (20)**. Light bronze oil, yield 80% (0.74 g); TLC: *R*_f_ = 0.74 (S_2_); UPLC (purity > 99%): *t*_R_ = 8.11 min. LC-MS (ESI): *m/z* calcd for C_23_H_27_N_4_O_3_F_3_ (M+H)^+^ 465.21 found 465.3. 

***Tert*-butyl (2-oxo-1-phenyl-2-(4-(5-(trifluoromethyl)pyridin-2-yl)piperazin-1-yl)ethyl) carbamate (21)**. Light bronze oil, yield 82% (0.76 g); TLC: *R*_f_ = 0.75 (S_2_); UPLC (purity > 99%): *t*_R_ = 8.17 min. LC-MS (ESI): *m/z* calcd for C_23_H_27_N_4_O_3_F_3_ (M+H)^+^ 465.21 found 465.5.

***Tert*-butyl (2-oxo-1-phenyl-2-(4-(6-(trifluoromethyl)pyridin-2-yl)piperazin-1-yl)ethyl) carbamate (22)**. Light bronze oil, yield 81% (0.75 g); TLC: *R*_f_ = 0.76 (S_2_); UPLC (purity > 99%): *t*_R_ = 8.29 min. LC-MS (ESI): *m/z* calcd for C_23_H_27_N_4_O_3_F_3_ (M+H)^+^ 465.21 found 465.3. 

#### 2.1.4. General Method for the Preparation of Intermediates **23**–**44**

The solution of **1**–**22** (1.5 mmol, 1 eq) in DCM (5 mL) was treated with TFA (4.5 mmol, 3 eq) and stirred at room temperature for 3 h. Afterwards, the organic solvents were evaporated to dryness. The resulting oil residue was dissolved in water (20 mL) and then 25% ammonium hydroxide was carefully added until pH 8. The aqueous layer was extracted with DCM (3 × 20 mL), dried over Na_2_SO_4_, and concentrated to give **23**–**44** as yellow or bronze oils. Intermediates **23**–**44** were advanced to the last step reaction without purification. The synthetic pathway is shown in Scheme 2 (see Section 3 Results and Discussion).

**2-Amino-2-phenyl-1-(4-phenylpiperazin-1-yl)ethan-1-one (23)**. Yellow oil, yield 95% (0.43 g); UPLC (purity > 99%): *t*_R_ = 3.94 min. LC-MS (ESI): *m/z* calcd for C_18_H_21_N_3_O (M+H)^+^ 296.18, found 296.3.

**2-Amino-1-(4-(2-chlorophenyl)piperazin-1-yl)-2-phenylethan-1-one (24)**. Yellow oil, yield 95% (0.47 g); UPLC (purity > 99%): *t*_R_ = 4.53 min. LC-MS (ESI): *m/z* calcd for C_18_H_20_N_3_OCl (M+H)^+^ 330.13, found 330.2.

**2-Amino-1-(4-(3-chlorophenyl)piperazin-1-yl)-2-phenylethan-1-one (25)**. Yellow oil, yield 96% (0.48 g); UPLC (purity > 99%): *t*_R_ = 4.58 min. LC-MS (ESI): *m/z* calcd for C_18_H_20_N_3_OCl (M+H)^+^ 330.13, found 330.2.

**2-Amino-1-(4-(4-chlorophenyl)piperazin-1-yl)-2-phenylethan-1-one (26)**. Yellow oil, yield 94% (0.46 g); UPLC (purity > 99%): *t*_R_ = 4.63 min. LC-MS (ESI): *m/z* calcd for C_18_H_20_N_3_OCl (M+H)^+^ 330.13, found 330.2.

**2-Amino-1-(4-(3,4-dichlorophenyl)piperazin-1-yl)-2-phenylethan-1-one (27).** Yellow oil, yield 93% (0.51 g); UPLC (purity > 99%): *t*_R_ = 5.76 min. LC-MS (ESI): *m/z* calcd for C_18_H_19_N_3_OCl_2_ (M+H)^+^ 364.09 found 364.4. 

**2-Amino-1-(4-(3,5-dichlorophenyl)piperazin-1-yl)-2-phenylethan-1-one (28)**. Yellow oil, yield 95% (0.52 g); UPLC (purity > 99%): *t*_R_ = 5.62 min. LC-MS (ESI): *m/z* calcd for C_18_H_19_N_3_OCl_2_ (M+H)^+^ 364.09 found 364.5. 

**2-Amino-1-(4-(3-chloro-5-(trifluoromethyl)phenyl)piperazin-1-yl)-2-phenylethan-1--one (29)**. Yellow oil, yield 94% (0.56 g); UPLC (purity > 99%): *t*_R_ = 5.68 min. LC-MS (ESI): *m/z* calcd for C_19_H_19_N_3_OClF_3_ (M+H)^+^ 398.12 found 398.4. 

**2-Amino-2-phenyl-1-(4-(2-(trifluoromethyl)phenyl)piperazin-1-yl)ethan-1-one (30)**. Yellow oil, yield 93% (0.51 g); UPLC (purity > 99%): *t*_R_ = 4.86 min. LC-MS (ESI): *m/z* calcd for C_19_H_20_N_3_OF_3_ (M+H)^+^ 364.16, found 364.2.

**2-Amino-2-phenyl-1-(4-(3-(trifluoromethyl)phenyl)piperazin-1-yl)ethan-1-one (31)**. Yellow oil, yield 97% (0.53 g); UPLC (purity > 99%): *t*_R_ = 4.89 min. LC-MS (ESI): *m/z* calcd for C_19_H_20_N_3_OF_3_ (M+H)^+^ 364.16, found 364.2.

**2-Amino-2-phenyl-1-(4-(4-(trifluoromethyl)phenyl)piperazin-1-yl)ethan-1-one (32)**. Yellow oil, yield 96% (0.53 g); UPLC (purity > 99%): *t*_R_ = 4.95 min. LC-MS (ESI): *m/z* calcd for C_19_H_20_N_3_OF_3_ (M+H)^+^ 364.16, found 364.2.

**2-Amino-1-(4-(3,5-bis(trifluoromethyl)phenyl)piperazin-1-yl)-2-phenylethan-1-one (33)**. Yellow oil, yield 97% (0.63 g); UPLC (purity > 99%): *t*_R_ = 5.81 min. LC-MS (ESI): *m/z* calcd for C_20_H_19_N_3_OF_6_ (M+H)^+^ 432.15 found 432.0. 

**2-Amino-2-phenyl-1-(4-(m-tolyl)piperazin-1-yl)ethan-1-one (34)**. Yellow oil, yield 94% (0.44 g); UPLC (purity > 99%): *t*_R_ = 4.31 min. LC-MS (ESI): *m/z* calcd for C_19_H_23_N_3_O (M+H)^+^ 310.19 found 310.4. 

**2-Amino-1-(4-(3-(difluoromethyl)phenyl)piperazin-1-yl)-2-phenylethan-1-one (35)**. Yellow oil, yield 96% (0.5 g); UPLC (purity > 99%): *t*_R_ = 4.34 min. LC-MS (ESI): *m/z* calcd for C_19_H_21_N_3_OF_2_ (M+H)^+^ 346.17 found 346.0. 

**1-(4-([1,1’-biphenyl]-3-yl)piperazin-1-yl)-2-amino-2-phenylethan-1-one (36)**. Yellow oil, yield 93% (0.52 g); UPLC (purity > 99%): *t*_R_ = 5.45 min. LC-MS (ESI): *m/z* calcd for C_24_H_25_N_3_O (M+H)^+^ 372.20 found 372.4. 

**2-Amino-1-(4-(3-methoxyphenyl)piperazin-1-yl)-2-phenylethan-1-one (37)**. Yellow oil, yield 95% (0.46 g); UPLC (purity > 99%): *t*_R_ = 3.93 min. LC-MS (ESI): *m/z* calcd for C_19_H_23_N_3_O_2_ (M+H)^+^ 326.18 found 326.2.

**2-Amino-2-phenyl-1-(4-(3-(trifluoromethoxy)phenyl)piperazin-1-yl)ethan-1-one (38)**. Yellow oil, yield 98% (0.59 g); UPLC (purity > 99%): *t*_R_ = 5.53 min. LC-MS (ESI): *m/z* calcd for C_19_H_20_N_3_O_2_F_3_ (M+H)^+^ 380.15 found 380.3. 

**2-Amino-1-(4-(3-phenoxyphenyl)piperazin-1-yl)-2-phenylethan-1-one (39)**. Yellow oil, yield 97% (0.56 g); UPLC (purity > 99%): *t*_R_ = 5.55 min. LC-MS (ESI): *m/z* calcd for C_24_H_25_N_3_O_2_ (M+H)^+^ 388.20 found 388.4. 

**2-Amino-2-phenyl-1-(4-(3-((trifluoromethyl)thio)phenyl)piperazin-1-yl)ethan-1-one (40)**. Yellow oil, yield 97% (0.58 g); UPLC (purity > 99%): *t*_R_ = 5.60 min. LC-MS (ESI): *m/z* calcd for C_19_H_20_N_3_OSF_3_ (M+H)^+^ 396.13 found 396.2. 

**2-Amino-2-phenyl-1-(4-(3-(trifluoromethyl)pyridin-2-yl)piperazin-1-yl)ethan-1-one (41)**. Bronze oil, yield 95% (0.52 g); UPLC (purity > 99%): *t*_R_ = 4.58 min. LC-MS (ESI): *m/z* calcd for C_18_H_19_N_4_OF_3_ (M+H)^+^ 365.15 found 365.4. 

**2-Amino-2-phenyl-1-(4-(4-(trifluoromethyl)pyridin-2-yl)piperazin-1-yl)ethan-1-one (42)**. Bronze oil, yield 94% (0.51g); UPLC (purity > 99%): *t*_R_ = 4.34 min. LC-MS (ESI): *m/z* calcd for C_18_H_19_N_4_OF_3_ (M+H)^+^ 365.15 found 365.2. 

**2-Amino-2-phenyl-1-(4-(5-(trifluoromethyl)pyridin-2-yl)piperazin-1-yl)ethan-1-one (43)**. Bronze oil, yield 96% (0.53 g); UPLC (purity > 99%): *t*_R_ = 4.61 min. LC-MS (ESI): *m/z* calcd for C_18_H_19_N_4_OF_3_ (M+H)^+^ 365.15 found 365.5. 

**2-Amino-2-phenyl-1-(4-(6-(trifluoromethyl)pyridin-2-yl)piperazin-1-yl)ethan-1-one (44)**. Bronze oil, yield 93% (0.51 g); UPLC (purity > 99%): *t*_R_ = 4.39 min. LC-MS (ESI): *m/z* calcd for C_18_H_19_N_4_OF_3_ (M+H)^+^ 365.15 found 365.2. 

#### 2.1.5. General Method for the Preparation of Final Compounds **45–66**

Intermediates **23**–**44** (1.3 mmol, 1 eq) were dissolved in 10 mL of DCM. Afterwards, triethylamine (TEA) (3.9 mmol, 3 eq) was added while stirring at 0 °C. The final compounds **45**–**66** were prepared by dropwise adding of acetyl chloride (2 mmol, 1.5 eq) at 0 °C (ice bath). The reaction mixture was allowed to warm up to room temperature and was stirred for an additional 1.5 h and then evaporated to dryness. The crude products were purified by applying column chromatography using developing system-S_3_. Compounds **45**–**66** were obtained as white solids followed by concentration of organic solvents under reduced pressure and wash-up with diethyl ether. The synthetic pathway is shown in Scheme 2 (see Section 3 Results and Discussion).

***N*-(2-oxo-1-phenyl-2-(4-phenylpiperazin-1-yl)ethyl)acetamide (45)**. White solid. Yield: 92% (0.4 g); mp 132.9–133.4 °C; TLC: R_f_ **=** 0.42 (S_3_); UPLC (purity > 99%): *t*_R_ = 5.59 min. LC-MS (ESI): *m/z* calcd for C_20_H_23_N_3_O_2_ (M+H)^+^ 338.18, found 338.2. ^1^H NMR (500 MHz, CDCl_3_) δ 1.98 (s, 3 H, CH_3_) 2.53 (d, *J* = 1.0 Hz, 1 H, piperazine) 2.88–3.10 (m, 2 H, piperazine) 3.15–3.26 (m, 1 H, piperazine) 3.39–3.50 (m, 1 H, piperazine) 3.54 (dd, *J* = 7.7, 2.9 Hz, 1 H, piperazine) 3.69 (br. S., 1 H, piperazine) 3.91 (dt, *J* = 6.3, 3.2 Hz, 1 H, piperazine) 5.92 (d, *J* = 7.5 Hz, 1 H, CH) 6.82 (d, *J* = 7.9 Hz, 2 H, ArH) 6.87 (t, *J* = 7.3 Hz, 1 H, ArH) 7.09 (d, *J* = 7.3 Hz, 1 H, NH) 7.20–7.26 (m, 2 H, ArH) 7.26–7.37 (m, 3 H, ArH) 7.37–7.43 (m, 2 H, ArH). ^13^C NMR (126 MHz, CDCl_3_) δ 23.4, 42.3, 45.4, 49.2, 49.2, 53.9, 116.7, 120.8, 127.9, 128.5, 129.3, 129.3, 137.8, 150.6, 168.3, 169.2.

***N*-(2-(4-(2-chlorophenyl)piperazin-1-yl)-2-oxo-1-phenylethyl)acetamide (46)**. White solid. Yield: 89% (0.43 g); mp 134.8–135.8 °C; TLC: R_f_ **=** 0.49 (S_3_); UPLC (purity > 99%): *t*_R_ = 6.46 min. LC-MS (ESI): *m/z* calcd for C_20_H_22_N_3_O_2_Cl (M+H)^+^ 372.14, found 372.1. ^1^H NMR (500 MHz, CDCl_3_) δ 1.97 (s, 3 H, CH_3_) 2.33–2.59 (m, 1 H, piperazine) 2.77–2.93 (m, 2 H, piperazine) 2.94–3.13 (m, 1 H, piperazine) 3.41 (dd, *J* = 6.6, 3.2 Hz, 1 H, piperazine) 3.51–3.67 (m, 1 H, piperazine) 3.72–3.99 (m, 2 H, piperazine) 5.92 (d, *J* = 7.4 Hz, 1 H, CH) 6.87 (d, *J* = 7.8 Hz, 1 H, ArH) 6.96 (td, *J* = 7.6, 1.5 Hz, 1 H, ArH) 7.12 (d, *J* = 7.4 Hz, 1 H, NH) 7.16 (td, *J* = 7.7, 1.5 Hz, 1 H, ArH) 7.26–7.36 (m, 4 H, ArH) 7.37–7.43 (m, 2 H, ArH). ^13^C NMR (126 MHz, CDCl_3_) δ 23.4, 42.6, 45.7, 50.9, 53.9, 120.6, 124.5 127.8, 127.9, 128.5, 128.9, 129.2, 130.8, 137.8, 148.3, 168.4, 169.2.

***N*-(2-(4-(3-chlorophenyl)piperazin-1-yl)-2-oxo-1-phenylethyl)acetamide (47)**. White solid. Yield: 93% (0.45 g); mp 145.5–146.7 °C; TLC: R_f_ **=** 0.50 (S_3_); UPLC (purity > 99%): *t*_R_ = 6.45 min. LC-MS (ESI): *m/z* calcd for C_20_H_22_N_3_O_2_Cl (M+H)^+^ 372.14, found 372.2. ^1^H NMR (500 MHz, CDCl_3_) δ 1.97 (s, 3 H, CH_3_) 2.41–2.65 (m, 1 H, piperazine) 2.90–3.13 (m, 2 H, piperazine) 3.14–3.28 (m, 1 H, piperazine) 3.37–3.48 (m, 1 H, piperazine) 3.51–3.61 (m, 1 H, piperazine) 3.60–3.75 (m, 1 H, piperazine) 3.92 (d, *J* = 13.3 Hz, 1 H, piperazine) 5.91 (d, *J* = 7.5 Hz, 1 H, CH) 6.68 (d, *J* = 8.3 Hz, 1 H, ArH) 6.76 (s, 1 H, ArH) 6.79–6.85 (m, 1 H, ArH) 7.07 (d, *J* = 6.3 Hz, 1 H, NH) 7.10–7.17 (m, 1 H, ArH) 7.27–7.36 (m, 3 H, ArH) 7.37–7.42 (m, 2 H, ArH). ^13^C NMR (126 MHz, CDCl_3_) δ 23.4, 42.1, 45.1, 48.7, 48.7, 53.9, 114.5, 116.5, 120.5, 127.9, 128.6, 129.3, 130.3, 135.1, 137.7, 151.6, 168.3, 169.2.

***N*-(2-(4-(4-chlorophenyl)piperazin-1-yl)-2-oxo-1-phenylethyl)acetamide (48)**. White solid. Yield: 88% (0.43 g); mp 133.3–134.6 °C; TLC: R_f_ **=** 0.49 (S_3_); UPLC (purity > 99%): *t*_R_ = 6.32 min. LC-MS (ESI): *m/z* calcd for C_20_H_22_N_3_O_2_Cl (M+H)^+^ 372.14, found 372.2. ^1^H NMR (500 MHz, CDCl_3_) δ 1.97 (s, 3 H, CH_3_) 2.48 (t, *J* = 8.4 Hz, 1 H, piperazine) 2.87–3.05 (m, 2 H, piperazine) 3.11–3.22 (m, 1 H, piperazine) 3.37–3.48 (m, 1 H, piperazine) 3.49–3.60 (m, 1 H, piperazine) 3.62–3.74 (m, 1 H, piperazine) 3.91 (dd, *J* = 6.1, 3.1 Hz, 1 H, piperazine) 5.91 (d, *J* = 7.4 Hz, 1 H, CH) 6.73 (d, *J* = 8.7 Hz, 2 H, ArH) 7.06 (d, *J* = 7.3 Hz, 1 H, NH) 7.16 (d, *J* = 8.9 Hz, 2 H, ArH) 7.26–7.31 (m, 1 H, ArH) 7.32–7.36 (m, 2 H, ArH) 7.36–7.41 (m, 2 H, ArH). ^13^C NMR (126 MHz, CDCl_3_) δ 23.4, 42.1, 45.2, 49.2, 49.3, 53.9, 118.0, 125.8, 127.9, 128.6, 129.2, 129.3, 137.7, 149.2, 168.3, 169.2.

***N*-(2-(4-(3,4-dichlorophenyl)piperazin-1-yl)-2-oxo-1-phenylethyl)acetamide (49)**. White solid. Yield: 93% (0.5 g); mp 147.9–148.7 °C; TLC: R_f_ **=** 0.48 (S_3_); UPLC (purity > 99%): *t*_R_ = 6.97 min. LC-MS (ESI): *m/z* calcd for C_20_H_21_N_3_O_2_Cl_2_ (M+H)^+^ 406.10, found 406.1. ^1^H NMR (500 MHz, CDCl_3_) δ 1.97 (s, 3 H, CH_3_) 2.39–2.57 (m, 1 H, piperazine) 2.87–3.08 (m, 2 H, piperazine) 3.13–3.24 (m, 1 H, piperazine) 3.36–3.46 (m, 1 H, piperazine) 3.48–3.58 (m, 1 H, piperazine) 3.59–3.70 (m, 1 H, piperazine) 3.92 (dd, *J* = 6.0, 3.3 Hz, 1 H, piperazine) 5.90 (d, *J* = 7.4 Hz, 1 H, CH) 6.62 (td, *J* = 8.9, 1.4 Hz, 1 H, ArH) 6.79–6.88 (m, 1 H, ArH) 7.03 (d, *J* = 7.2 Hz, 1 H, NH) 7.19–7.26 (m, 1 H, ArH) 7.27–7.36 (m, 3 H, ArH) 7.36–7.40 (m, 2 H, ArH). ^13^C NMR (126 MHz, CDCl_3_) δ 23.4, 42.0, 45.0, 48.6, 48.6, 53.9, 115.9, 117.9, 123.3, 127.9, 128.6, 129.3, 130.7, 133.0, 137.6, 150.1, 168.3 169.2.

***N*-(2-(4-(3,5-dichlorophenyl)piperazin-1-yl)-2-oxo-1-phenylethyl)acetamide (50)**. White solid. Yield: 90% (0.48 g); mp 185.9–187.3 °C; TLC: R_f_ **=** 0.52 (S_3_); UPLC (purity > 99%): *t*_R_ = 7.27 min. LC-MS (ESI): *m/z* calcd for C_20_H_21_N_3_O_2_Cl_2_ (M+H)^+^ 406.10, found 406.2. ^1^H NMR (500 MHz, CDCl_3_) δ 1.98 (s, 3 H, CH_3_) 2.39–2.61 (m, 1 H, piperazine) 2.86–3.12 (m, 2 H, piperazine) 3.15–3.28 (m, 1 H, piperazine) 3.36–3.48 (m, 1 H, piperazine) 3.48–3.56 (m, 1 H, piperazine) 3.57–3.69 (m, 1 H, piperazine) 3.93 (dd, *J* = 5.9, 3.3 Hz, 1 H, piperazine) 5.90 (d, *J* = 7.4 Hz, 1 H, CH) 6.62 (s, 2 H, ArH) 6.80 (d, *J* = 1.3 Hz, 1 H, ArH) 7.02 (d, *J* = 7.3 Hz, 1 H, NH) 7.27–7.37 (m, 3 H, ArH) 7.38–7.41 (m, 2 H, ArH). ^13^C NMR (126 MHz, CDCl_3_) δ 23.4, 41.9, 44.9, 48.1, 48.2, 53.9, 114.4, 119.9, 127.9, 128.7, 129.4, 135.7, 137.6, 152.0, 168.4, 169.2.

***N*-(2-(4-(3-chloro-5-(trifluoromethyl)phenyl)piperazin-1-yl)-2-oxo-1-phenylethyl) acetamide (51)**. White solid. Yield: 92% (0.53 g); mp 187.1–188.2 °C; TLC: R_f_ **=** 0.45 (S_3_); UPLC (purity > 99%): *t*_R_ = 7.40 min. LC-MS (ESI): *m/z* calcd for C_21_H_21_N_3_O_2_ClF_3_ (M+H)^+^ 440.13, found 440.2. ^1^H NMR (500 MHz, CDCl_3_) δ 1.98 (s, 3 H, CH_3_) 2.57 (br. S., 1 H, piperazine) 3.02–3.09 (m, 1 H, piperazine) 3.11 (br. S., 1 H, piperazine) 3.22–3.31 (m, 1 H, piperazine) 3.42–3.49 (m, 1 H, piperazine) 3.54 (dd, *J* = 7.9, 3.3 Hz, 1 H, piperazine) 3.62–3.71 (m, 1 H, piperazine) 3.94 (br. S., 1 H, piperazine) 5.91 (d, *J* = 7.4 Hz, 1 H, CH) 6.83–6.90 (m, 2 H, ArH) 7.01 (d, *J* = 7.4 Hz, 1 H, NH) 7.04 (s, 1 H, ArH) 7.28–7.37 (m, 3 H, ArH) 7.38–7.41 (m, 2 H, ArH). ^13^C NMR (126 MHz, CDCl_3_)) δ 23.4, 41.9, 44.9, 48.0, 48.1, 53.9, 110.8 (q, *J* = 4.0 Hz), 116.6 (q, *J* = 3.6 Hz), 118.8, 123.4, (q, *J* = 272.8 Hz), 127.9, 128.7, 129.4, 132.8, (q, *J* = 32.6 Hz) 135.8, 137.6, 151.7, 168.4, 169.2.

***N*-(2-oxo-1-phenyl-2-(4-(2-(trifluoromethyl)phenyl)piperazin-1-yl)ethyl)acetamide (52)**. White solid. Yield: 88% (0.46 g); mp 147.2–148.3 °C; TLC: R_f_ **=** 0.52 (S_3_); UPLC (purity > 99%): *t*_R_ = 6.87 min. LC-MS (ESI): *m/z* calcd for C_21_H_22_N_3_O_2_F_3_ (M+H)^+^ 406.17, found 406.3. ^1^H NMR (500 MHz, CDCl_3_) δ 1.98 (s, 3 H, CH_3_) 2.38 (d, *J* = 6.9 Hz, 1 H, piperazine) 2.66–2.98 (m, 3 H, piperazine) 3.23–3.40 (m, 1 H, piperazine) 3.56 (dd, *J* = 6.7, 2.6 Hz, 1 H, piperazine) 3.78 (d, *J* = 10.1 Hz, 2 H, piperazine) 5.92 (d, *J* = 7.4 Hz, 1 H, CH) 7.08–7.17 (m, 2 H, ArH) 7.21 (t, *J* = 7.7 Hz, 1H) 7.27–7.37 (m, 3 H, NH, ArH) 7.40–7.43 (m, 2 H, ArH) 7.44–7.50 (m, 1 H, ArH) 7.58 (dd, *J* = 7.8, 1.04 Hz, 1 H, ArH). ^13^C NMR (126 MHz, CDCl) δ 23.4, 42.9, 46.0, 52.9, 53.0, 53.9, 123.7 (q, *J* = 272.8 Hz), 124.1, 125.6, 127.4 (q, *J* = 32.0 Hz) 127.9, 128.5, 129.2, 129.3, 133.0, 137.8, 151.4, 168.3, 169.2.

***N*-(2-oxo-1-phenyl-2-(4-(3-(trifluoromethyl)phenyl)piperazin-1-yl)ethyl)acetamide (53)**. White solid. Yield: 93% (0.49 g); mp 131.8–132.5 °C; TLC: R_f_ **=** 0.53 (S_3_); UPLC (purity > 99%): *t*_R_ = 6.72 min. LC-MS (ESI): *m/z* calcd for C_21_H_22_N_3_O_2_F_3_ (M+H)^+^ 406.17, found 406.3. ^1^H NMR (500 MHz, CDCl_3_) δ 1.97 (s, 3 H, CH_3_) 2.57 (td, *J* = 7.9, 3.8 Hz, 1 H, piperazine) 2.95–3.15 (m, 2 H, piperazine) 3.19–3.31 (m, 1 H, piperazine) 3.47 (dd, *J* = 6.1, 3.2 Hz, 1 H, piperazine) 3.52–3.61 (m, 1 H, piperazine) 3.62–3.75 (m, 1 H, piperazine) 3.92–3.96 (m, 1 H, piperazine) 5.92 (d, *J* = 7.4 Hz, 1 H, CH) 6.95 (dd, *J* = 8.3, 2.2 Hz, 1 H, NH) 7.00 (s, 1 H, ArH) 7.05–7.12 (m, 2 H, ArH) 7.26–7.37 (m, 4 H, ArH) 7.38–7.42 (m, 2 H, ArH). ^13^C NMR (126 MHz, CDCl_3_) δ 23.3, 42.1, 45.1, 48.6, 48.7, 53.9, 112.9 (d, *J* = 3.62 Hz), 117.0 (d, *J* = 3.62 Hz), 119.4, 124.12 (q, *J* = 272.8 Hz) 127.9, 128.6, 129.3, 129.8, 131.65 (q, *J* = 32.0 Hz) 137.7 150.8, 168.4, 169.2.

***N*-(2-oxo-1-phenyl-2-(4-(4-(trifluoromethyl)phenyl)piperazin-1-yl)ethyl)acetamide (54)**. White solid. Yield: 90% (0.47 g); mp 154.7–155.4 °C; TLC: R_f_ = 0.51 (S_3_); UPLC (purity > 99%): *t*_R_ = 6.74 min. LC-MS (ESI): *m/z* calcd for C_21_H_22_N_3_O_2_F_3_ (M+H)^+^ 406.17, found 406.2. ^1^H NMR (500 MHz, CDCl_3_) δ 1.98 (s, 3 H, CH_3_) 2.62 (td, *J* = 8.0, 3.8 Hz, 1 H, piperazine) 2.99–3.22 (m, 2 H, piperazine) 3.28–3.40 (m, 1 H, piperazine) 3.46 (dd, *J* = 6.2, 3.2 Hz, 1 H, piperazine) 3.54–3.63 (m, 1 H, piperazine) 3.63–3.75 (m, 1 H, piperazine) 3.93 (dd, *J* = 6.5, 3.1 Hz, 1 H, piperazine) 5.92 (d, *J* = 7.5 Hz, 1 H, CH) 6.82 (d, *J* = 8.7 Hz, 2 H, ArH) 7.05 (d, *J* = 7.4 Hz, 1 H, NH) 7.26–7.37 (m, 3 H, ArH) 7.38–7.41 (m, 2 H, ArH) 7.44 (d, *J* = 8.7 Hz, 2 H, ArH). ^13^C NMR (126 MHz, CDCl_3_) δ 23.4, 42.0, 45.0, 47.9, 48.0, 53.9, 115.2, 121.7 (q, *J* = 32.6 Hz), 124.9 (q, *J* = 271.6 Hz), 126.6, 127.9, 128.6, 129.3, 137.6, 152.6, 168.4, 169.2.

***N*-(2-(4-(3,5-bis(trifluoromethyl)phenyl)piperazin-1-yl)-2-oxo-1-phenylethyl)acetamide(55)**. White solid. Yield: 88% (0.54 g); mp 194.2–195.4 °C; TLC: R_f_ = 0.43 (S_3_); UPLC (purity > 99%): *t*_R_ = 7.62 min. LC-MS (ESI): *m/z* calcd for C_22_H_21_N_3_O_2_F_6_ (M+H)^+^ 474.16, found 474.2. ^1^H NMR (500 MHz, CDCl_3_) δ 1.99 (s, 3 H, CH_3_) 2.64 (br. S., 1 H, piperazine) 3.01–3.15 (m, 1 H, piperazine) 3.16–3.22 (m, 1 H, piperazine) 3.28–3.40 (m, 1 H, piperazine) 3.44–3.52 (m, 1 H, piperazine) 3.58 (dd, *J* = 7.8, 3.3 Hz, 1 H, piperazine) 3.64–3.74 (m, 1 H, piperazine) 3.96 (d, *J* = 2.6 Hz, 1 H, piperazine) 5.92 (d, *J* = 7.5 Hz, 1 H, CH) 7.03 (d, *J* = 7.4 Hz, 1 H, NH) 7.13 (s, 2 H, ArH) 7.26–7.43 (m, 6 H, ArH). ^13^C NMR (126 MHz, CDCl_3_) δ 23.3, 41.9, 44.9, 48.0, 53.9, 113.0 (t, *J* = 2.4 Hz), 115.3 (d, *J* = 2.4 Hz), 123.4 (q, *J* = 272.8 Hz), 127.9, 128.7, 129.4, 129.4, 132.6 (q, *J* = 32.6 Hz) 137.49, 151.1, 168.5, 169.3.

***N*-(2-oxo-1-phenyl-2-(4-(m-tolyl)piperazin-1-yl)ethyl)acetamide (56)**. White solid. Yield: 94% (0.43 g); mp 162.9–163.7 °C; TLC: R_f_ = 0.44 (S_3_); UPLC (purity > 99%): *t*_R_ = 6.03 min. LC-MS (ESI): *m/z* calcd for C_21_H_25_N_3_O_2_ (M+H)^+^ 352.20, found 352.1. ^1^H NMR (500 MHz, CDCl_3_) δ 1.98 (s, 3 H, CH_3_) 2.27 (s, 3 H, CH_3_) 2.53 (td, *J* = 7.9, 3.9 Hz, 1 H, piperazine) 2.88–3.11 (m, 2 H, piperazine) 3.13–3.28 (m, 1 H, piperazine) 3.44 (dd, *J* = 6.2, 3.2 Hz, 1 H, piperazine) 3.49–3.59 (m, 1 H, piperazine) 3.67 (dd, *J* = 7.9, 3.3 Hz, 1 H, piperazine) 3.91 (dd, *J* = 6.4, 3.1 Hz, 1 H, piperazine) 5.92 (d, *J* = 7.4 Hz, 1 H, CH) 6.59–6.65 (m, 2 H, ArH) 6.69 (d, *J* = 7.4 Hz, 1 H, NH) 7.05–7.17 (m, 2 H, ArH) 7.26–7.36 (m, 3 H, ArH) 7.38–7.42 (m, 2 H, ArH). ^13^C NMR (126 MHz, CDCl_3_) δ 21.8, 23.4, 42.3, 45.4, 49.2, 53.9, 113.8, 117.6, 121.6, 127.9, 128.5, 129.2, 129.3, 137.8, 139.1, 150.8, 168.3, 169.2.

***N*-(2-(4-(3-(difluoromethyl)phenyl)piperazin-1-yl)-2-oxo-1-phenylethyl)acetamide (57)**. White solid. Yield: 84% (0.42 g); mp 157.7–158.5 °C; TLC: R_f_ = 0.49 (S_3_); UPLC (purity > 99%): *t*_R_ = 5.93 min. LC-MS (ESI): *m/z* calcd for C_21_H_23_N_3_O_2_F_2_ (M+H)^+^ 388.18, found 388.0. ^1^H NMR (500 MHz, CDCl_3_) δ 1.98 (s, 3 H, CH_3_) 2.56 (td, *J* = 7.9, 3.8 Hz, 1 H, piperazine) 2.96–3.15 (m, 2 H, piperazine) 3.18–3.33 (m, 1 H, piperazine) 3.37–3.50 (m, 1 H, piperazine) 3.51–3.60 (m, 1 H, piperazine) 3.68 (dd, *J* = 7.9, 3.3 Hz, 1 H, piperazine) 3.94 (dd, *J* = 6.4, 3.0 Hz, 1 H, piperazine) 5.92 (d, *J* = 7.4 Hz, 1 H, CH) 6.54 (s, 1 H, CHF_2_) 6.83–6.94 (m, 2 H, ArH) 6.97 (d, *J* = 7.5 Hz, 1 H, ArH) 7.04 (d, *J* = 7.4 Hz, 1 H, NH) 7.26–7.37 (m, 4 H, ArH) 7.38–7.44 (m, 2 H, ArH). ^13^C NMR (126 MHz, CDCl_3_) δ 23.4, 42.2, 45.2, 48.7, 48.8, 53.9, 113.1 (t, *J* = 6.0 Hz) 114.8, 117.6 (t, *J* = 6.6 Hz), 118.6, 127.9, 128.6, 129.3 129.7, 135.5 (t, *J* = 21.7 Hz) 137.7, 151.0, 168.3, 169.2.

***N*-(2-(4-([1,1’-biphenyl]-3-yl)piperazin-1-yl)-2-oxo-1-phenylethyl)acetamide (58)**. White solid. Yield: 86% (0.46 g); mp 173.7–174.8 °C; TLC: R_f_ = 0.44 (S_3_); UPLC (purity > 99%): *t*_R_ = 7.09 min. LC-MS (ESI): *m/z* calcd for C_26_H_27_N_3_O_2_ (M+H)^+^ 414.21, found 414.4. ^1^H NMR (500 MHz, CDCl_3_) δ 1.99 (s, 3 H, CH_3_) 2.60 (td, *J* = 7.9, 3.4 Hz, 1 H, piperazine) 2.98–3.17 (m, 2 H, piperazine) 3.20–3.33 (m, 1 H, piperazine) 3.41–3.51 (m, 1 H, piperazine) 3.51–3.63 (m, 1 H, piperazine) 3.70 (dd, *J* = 7.9, 3.2 Hz, 1 H, piperazine) 3.96 (dd, *J* = 6.4, 3.1 Hz, 1 H, piperazine) 5.93 (d, *J* = 7.4 Hz, 1 H, CH) 6.80 (dd, *J* = 2.4, 0.7 Hz, 1 H, ArH) 6.97–7.03 (m, 1 H, ArH) 7.09 (dd, *J* = 7.6, 1.5 Hz, 2 H, NH, ArH) 7.27–7.32 (m, 2 H, ArH) 7.32–7.37 (m, 3 H, ArH) 7.38–7.44 (m, 4 H, ArH) 7.48–7.55 (m, 2 H, ArH). ^13^C NMR (126 MHz, CDCl_3_) δ 23.4, 42.3, 45.4, 49.1, 49.2, 53.9, 115.6, 115.7, 119.8, 127.3 127.5, 127.9, 128.6, 128.8, 129.3, 129.7, 137.8, 141.5, 142.6, 151.1, 168.3, 169.2.

***N*-(2-(4-(3-methoxyphenyl)piperazin-1-yl)-2-oxo-1-phenylethyl)acetamide (59)**. White solid. Yield: 91% (0.43 g); mp 174.9–175.8 °C; TLC: R_f_ = 0.46 (S_3_); UPLC (purity > 99%): *t*_R_ = 5.59 min. LC-MS (ESI): *m/z* calcd for C_21_H_25_N_3_O_3_ (M+H)^+^ 368.19, found 368.0. ^1^H NMR (500 MHz, CDCl_3_) δ 1.97 (s, 3 H, CH_3_) 2.42–2.58 (m, 1 H, piperazine) 2.91–3.08 (m, 2 H, piperazine) 3.14–3.25 (m, 1 H, piperazine) 3.35–3.48 (m, 1 H, piperazine) 3.50–3.57 (m, 1 H, piperazine) 3.59–3.70 (m, 1 H, piperazine) 3.74 (s, 3 H, CH_3_) 3.91 (dd, *J* = 6.2, 3.3 Hz, 1 H, piperazine) 5.91 (d, *J* = 7.4 Hz, 1 H, CH) 6.26–6.53 (m, 3 H, ArH) 7.09 (d, *J* = 7.4 Hz, 1 H, NH) 7.13 (t, *J* = 8.2 Hz, 1 H, ArH) 7.26–7.35 (m, 3 H, ArH) 7.37–7.42 (m, 2 H, ArH). ^13^C NMR (126 MHz, CDCl_3_) δ 23.4, 42.3, 45.3, 49.00, 49.1, 53.9, 55.3, 103.2, 105.3, 109.3, 127.9, 128.5, 129.3, 130.0, 137.8, 152.1, 160.7, 168.3, 169.2.

***N*-(2-oxo-1-phenyl-2-(4-(3-(trifluoromethoxy)phenyl)piperazin-1-yl)ethyl)acetamide (60)**. White solid. Yield: 91% (0.50 g); mp 133.3–135.1 °C; TLC: R_f_ = 0.52 (S_3_); UPLC (purity > 99%): *t*_R_ = 7.18 min. LC-MS (ESI): *m/z* calcd for C_21_H_22_N_3_O_3_F_3_ (M+H)^+^ 422.16, found 422.4. ^1^H NMR (500 MHz, CDCl_3_) δ 1.98 (s, 3 H, CH_3_) 2.50–2.58 (m, 1 H, piperazine) 2.97–3.11 (m, 2 H, piperazine) 3.18–3.26 (m, 1 H, piperazine) 3.40–3.47 (m, 1 H, piperazine) 3.53 (dd, *J* = 7.8, 3.3 Hz, 1 H, piperazine) 3.63–3.70 (m, 1 H, piperazine) 3.91 (d, *J* = 2.9 Hz, 1 H, piperazine) 5.91 (d, *J* = 7.5 Hz, 1 H, CH) 6.59 (s, 1 H, ArH) 6.66–6.73 (m, 2 H, ArH) 7.07 (d, *J* = 7.4 Hz, 1 H, NH) 7.20 (t, *J* = 8.3 Hz, 1 H, ArH) 7.27–7.36 (m, 3 H, ArH) 7.38–7.41 (m, 2 H, ArH). ^13^C NMR (126 MHz,, CDCl_3_) δ 23.4, 42.1, 45.1, 48.5, 48.5, 53.9, 108.9, 112.2, 114.3, 120.5 (q, *J* = 272.8 Hz), 128.2 (q, *J* = 32.8 Hz), 127.9, 128.6, 129.3, 130.3, 137.7, 150.3 (d, *J* = 1.2 Hz), 152.0, 168.4, 169.2.

***N*-(2-oxo-2-(4-(3-phenoxyphenyl)piperazin-1-yl)-1-phenylethyl)acetamide (61)**. White solid. Yield: 92% (0.51 g); mp 144.8–145.9 °C; TLC: R_f_ = 0.53 (S_3_); UPLC (purity > 99%): *t*_R_ = 7.11 min. LC-MS (ESI): *m/z* calcd for C_26_H_27_N_3_O_3_ (M+H)^+^ 430.21, found 430.4. ^1^H NMR (500 MHz, CDCl_3_) δ 1.98 (s, 3 H, CH_3_) 2.47–2.56 (m, 1 H, piperazine) 2.95–3.07 (m, 2 H, piperazine) 3.15–3.24 (m, 1 H, piperazine) 3.42 (dd, *J* = 6.2, 3.2 Hz, 1 H, piperazine) 3.49–3.57 (m, 1 H, piperazine) 3.62–3.70 (m, 1 H, piperazine) 3.86–3.94 (m, 1 H, piperazine) 5.90 (d, *J* = 7.4 Hz, 1 H, CH) 6.43–6.50 (m, 2 H, ArH) 6.54 (dd, *J* = 2.1, 0.9 Hz, 1 H, ArH) 6.93–6.99 (m, 2 H, ArH) 7.03–7.11 (m, 2 H, ArH, NH) 7.12–7.18 (m, 1 H, ArH) 7.26–7.37 (m, 5 H, ArH) 7.37–7.41 (m, 2 H, ArH). ^13^C NMR (126 MHz, CDCl_3_) δ 23.4, 42.2, 45.2, 48.7, 48.8, 53.9, 107.2, 110.6, 111.2, 119.0, 123.3, 127.9, 128.5, 129.3, 129.8, 130.2, 137.8, 152.2, 157.1, 158.3, 168.3, 169.14.

***N*-(2-oxo-1-phenyl-2-(4-(3-((trifluoromethyl)thio)phenyl)piperazin-1-yl)ethyl)acet--amide (62)**. White solid. Yield: 89% (0.51 g); mp 126.3–127.1 °C; TLC: R_f_ = 0.51 (S_3_); UPLC (purity > 99%): *t*_R_ = 7.22 min. LC-MS (ESI): *m/z* calcd for C_21_H_22_N_3_O_2_SF_3_ (M+H)^+^ 438.14, found 438.3.^1^H NMR (500 MHz, CDCl_3_) δ 1.98 (s, 3 H, CH_3_) 2.56 (dd, *J* = 7.9, 4.2 Hz, 1 H, piperazine) 2.95–3.12 (m, 2 H, piperazine) 3.18–3.28 (m, 1 H, piperazine) 3.46 (dd, *J* = 6.6, 3.5 Hz, 1 H, piperazine) 3.51–3.61 (m, 1 H, piperazine) 3.63–3.73 (m, 1 H, piperazine) 3.92 (dd, *J* = 6.2, 3.4 Hz, 1 H, piperazine) 5.91 (d, *J* = 7.4 Hz, 1 H, CH) 6.89 (dd, *J* = 2.5, 0.7 Hz, 1 H, ArH) 7.01–7.06 (m, 2 H, ArH) 7.12 (d, *J* = 7.7 Hz, 1 H, NH) 7.20–7.27 (m, 1 H, ArH) 7.28–7.32 (m, 1 H, ArH) 7.33–7.37 (m, 2 H, ArH) 7.38–7.42 (m, 2 H, ArH). ^13^C NMR (126 MHz, CDCl_3_) δ 23.4, 42.1, 45.1, 48.5, 48.6, 53.9, 118.5, 123.7, 125.3, 129.7 (q, *J* = 308.4 Hz) 127.8, 127.9, 128.6, 129.3, 130.1, 137.7, 151.3, 168.4, 169.2.

***N*-(2-oxo-1-phenyl-2-(4-(3-(trifluoromethyl)pyridine-2-yl)piperazin-1-yl)ethyl)acetamide (63)**. White solid. Yield: 89% (0.47 g); mp 147.8–148.9 °C; TLC: R_f_ = 0.49 (S_3_); UPLC (purity > 99%): *t*_R_ = 6.03 min. LC-MS (ESI): *m/z* calcd for C_20_H_21_N_4_O_2_F_3_ (M+H)^+^ 407.17, found 407.3. ^1^H NMR (500 MHz, CDCl_3_) δ 1.97 (s, 3 H, CH_3_) 2.68–2.76 (m, 1 H, piperazine) 3.12–3.15 (m, 2 H, piperazine) 3.22–3.29 (m, 1 H, piperazine) 3.35–3.42 (m, 1 H, piperazine) 3.51–3.59 (m, 1 H, piperazine) 3.67–3.75 (m, 1 H, piperazine) 3.85 (dd, *J* = 6.5, 3.2 Hz, 1 H, piperazine) 5.91 (d, *J* = 7.4 Hz, 1 H, CH) 7.01 (dd, *J* = 4.8, 0.7 Hz, 1 H, ArH) 7.07 (d, *J* = 7.4 Hz, 1 H, NH) 7.26–7.31 (m, 1 H, ArH) 7.32–7.37 (m, 2 H, ArH) 7.37–7.42 (m, 2 H, ArH) 7.84 (dd, *J* = 7.8, 1.7 Hz, 1 H, ArH) 8.38 (dd, *J* = 4.8, 1.4 Hz, 1 H, ArH). ^13^C NMR (126 MHz, CDCl_3_) δ 23.4, 42.4, 45.5, 50.3, 50.5, 53.8, 117.7 (q, *J* = 32.0 Hz), 118.0, 123.79 (q, *J* = 272.8 Hz) 127.9, 128.5, 129.3, 137.3 (q, *J* = 5.2 Hz), 137.8, 151.2, 159.4, 168.4, 169.1.

***N*-(2-oxo-1-phenyl-2-(4-(4-(trifluoromethyl)pyridine-2-yl)piperazin-1-yl)ethyl)acetamide (64)**. White solid. Yield: 92% (0.49 g); mp 91.4–92.3 °C; TLC: R_f_ = 0.45 (S_3_); UPLC (purity > 99%): *t*_R_ = 6.08 min. LC-MS (ESI): *m/z* calcd for C_20_H_21_N_4_O_2_F_3_ (M+H)^+^ 407.17, found 407.2. ^1^H NMR (500 MHz, CDCl_3_) δ 1.97 (s, 3 H, CH_3_) 3.18 (br. S., 1 H, piperazine) 3.48 (d, *J* = 11.8 Hz, 1 H, piperazine) 3.54–3.67 (m, 2 H, piperazine) 3.68–3.79 (m, 2 H, piperazine) 3.85 (d, *J* = 12.1 Hz, 1 H, piperazine) 3.90–3.97 (m, 1 H, piperazine) 5.89 (d, *J* = 7.4 Hz, 1 H, CH) 6.80–6.95 (m, 2 H, ArH) 7.02 (d, *J* = 7.3 Hz, 1 H, NH) 7.26–7.36 (m, 3 H, ArH) 7.36–7.42 (m, 2 H, ArH) 8.28 (d, *J* = 5.6 Hz, 1 H, ArH). ^13^C NMR (126 MHz, CDCl_3_) δ 23.3 41.6, 44.6, 45.5, 45.7, 54.0, 105.4, 108.9 (d, *J* = 2.4 Hz), 122.3 (q, *J* = 274.0 Hz), 127.9, 128.8, 129.5, 137.2, 142.4 (q, *J* = 31.4 Hz), 155.8, 168.8, 169.3.

***N*-(2-oxo-1-phenyl-2-(4-(5-(trifluoromethyl)pyridine-2-yl)piperazin-1-yl)ethyl)acetamide (65)**. White solid. Yield: 88% (0.46 g); mp 91.2–92.4 °C; TLC: R_f_ = 0.48 (S_3_); UPLC (purity > 99%): *t*_R_ = 6.20 min. LC-MS (ESI): *m/z* calcd for C_20_H_21_N_4_O_2_F_3_ (M+H)^+^ 407.17, found 407.3. ^1^H NMR (500 MHz, CDCl_3_) δ 1.98 (s, 3 H, CH_3_) 2.95–3.04 (m, 1 H, piperazine) 3.37–3.47 (m, 2 H, piperazine) 3.49 (dd, *J* = 7.8, 3.4 Hz, 1 H, piperazine) 3.57–3.66 (m, 2 H, piperazine) 3.70–3.76 (m, 1 H, piperazine) 3.90 (d, *J* = 2.4 Hz, 1 H, piperazine) 5.91 (d, *J* = 7.4 Hz, 1 H, CH) 6.54 (d, *J* = 8.9 Hz, 1 H, ArH) 7.04 (d, *J* = 7.3 Hz, 1 H, NH) 7.27–7.37 (m, 3 H, ArH) 7.37–7.42 (m, 2 H, ArH) 7.56–7.64 (m, 1 H, ArH) 8.34 (td, *J* = 1.6, 0.9 Hz, 1 H, ArH). ^13^C NMR (126 MHz, CDCl_3_) δ 23.4, 41.9, 44.0, 44.3, 44.9, 54.0, 105.7, 116.1 (q, *J* = 33.2 Hz), 126.2 (q, *J* = 231.8 Hz), 127.9, 128.6, 129.4, 134.8 (d, *J* = 3.0 Hz), 137.6, 145.8 (q, *J* = 4.0 Hz), 159.9, 168.6, 169.2.

***N*-(2-oxo-1-phenyl-2-(4-(6-(trifluoromethyl)pyridine-2-yl)piperazin-1-yl)ethyl)acetamide (66)**. White solid. Yield: 91% (0.48 g); mp 151.6–152.3 °C; TLC: R_f_ = 0.51 (S_3_); UPLC (purity > 99%): *t*_R_ = 6.45 min. LC-MS (ESI): *m/z* calcd for C_20_H_21_N_4_O_2_F_3_ (M+H)^+^ 407.17, found 407.2. ^1^H NMR (500 MHz, CDCl_3_) δ 1.98 (s, 3 H, CH_3_) 2.83–3.05 (m, 1 H, piperazine) 3.34–3.44 (m, 2 H, piperazine) 3.49 (dd, *J* = 7.8, 3.4 Hz, 1 H, piperazine) 3.55–3.65 (m, 2 H, piperazine) 3.65–3.73 (m, 1 H, piperazine) 3.89 (d, *J* = 2.4 Hz, 1 H, piperazine) 5.91 (d, *J* = 7.5 Hz, 1 H, CH) 6.68 (d, *J* = 8.7 Hz, 1 H, ArH) 6.94 (d, *J* = 7.3 Hz, 1 H, ArH) 7.06 (d, *J* = 7.4 Hz, 1 H, NH) 7.26–7.36 (m, 3 H, ArH) 7.38–7.42 (m, 2 H, ArH) 7.55 (dd, *J* = 8.0, 0.5 Hz, 1 H, ArH). ^13^C NMR (126 MHz, CDCl_3_) δ 23.4, 41.9, 44.1, 44.4, 44.9, 54.0, 109.6, 109.7 (d, *J* = 2.4 Hz) 121.52 (q, *J* = 274.0), 127.9, 128.6, 129.3, 137.6, 138.7, 146.5 (q, *J* = 33.8 Hz), 158.3, 168.5, 169.2. 

### 2.2. In Silico Studies

Lipinski’s rule of five (RO5) parameters i.e., molecular weight (MW), lipophilicity (log P), number of hydrogen bond donors (NHD), number of hydrogen bond acceptors (NHA), as well as Veber’s rule i.e., number of rotatable bonds (NBR) and polar surface area (TPSA) were calculated using the SwissAdme software [46]. Central Nervous System Multi-Parameter Optimization (CNS MPO) parameters were determined using the Instant JChem 21.4.0 software (ChemAxon, Budapest, Hungary) All parameters calculated are summarized in Table 1 (see Section 3 Results and Discussion).

### 2.3. In Vivo Studies 

#### 2.3.1. Animals

Adult male Albino Swiss mice that weighed between 22 and 26 g were used in the in vivo studies. They were housed under standardized housing conditions in colony cages and had free access to food as well as tap water. The animals were left to adapt under laboratory conditions for 7 days. All procedures involving animals and their care were performed in accordance with the current European Community and Polish legislation on animal experimentation. The studies were carried out under experimental protocols that were approved by the Local Ethical Committee in Lublin (license no. 144/2018, 85/2019, 13/2021, and 25/2021), and in accordance with the European Communities Council Directive of 22 September 2010 (2010/63/EU).

#### 2.3.2. Anticonvulsant Activity and Acute Neurotoxicity

In the initial screening studies, four mice per group were randomly assigned to each experimental group (each mouse was used only once). To evaluate the ED_50_ or TD_50_ values, 3–4 groups that consisted of eight animals were injected with various doses of tested compounds. The protective indexes (PIs) for the compounds that were investigated and reference ASDs were calculated by dividing the TD_50_ value, as determined in the chimney test (or rotarod test), by the respective ED_50_ value, as determined in the MES, *sc*PTZ, or 6 Hz (32 mA or 44 mA) tests (PI = TD_50_/ED_50_). The PIs is a measure of the potential therapeutic window of the tested agent.

All substances were suspended in Tween 80 (1% aqueous solution) and administered *i.p.* as a single injection at a dose of 10 mL/kg. On each day of experimentation, fresh solutions were prepared. The results are summarized in Table S1 (screening data) and Table 2 (ED_50_, TD_50_, and PI values, see Section 3 Results and Discussion).

The detailed in vivo procedures are described elsewhere: the maximal electroshock seizure test (MES) [47], the subcutaneous pentylenetetrazole seizure test (*sc*PTZ) [48], the 6 Hz psychomotor seizure model (32 mA and 44 mA) [49], and the chimney test [50]. 

The reference ASDs were purchased form commercial suppliers: VPA (Sigma-Aldrich, St. Louis, MO, USA), LCS and LEV (UCB Pharma, Braine l’Alleud, Belgium). 

#### 2.3.3. Timed *iv*PTZ Seizure Threshold and Grip Strength Tests

**Figure 2:**
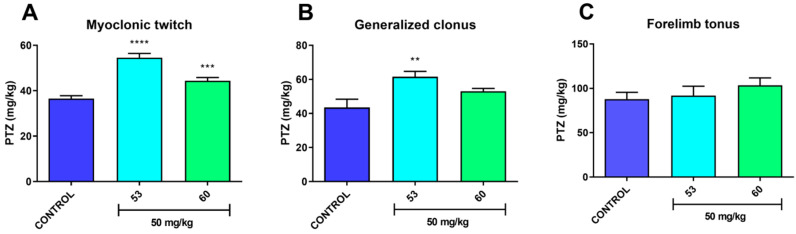
Acute effects of **53** and **60** on the threshold for the first myoclonic twitch (**A**), generalized clonus (**B**), and forelimb tonus (**C**) in the *iv*PTZ seizure threshold test in mice. Compounds **53** and **60** were administered *i.p*. 0.5 h before the seizure test. Control animals received vehicle only. Each experimental group consisted of 9–12 animals. Each bar represents the mean (mg/kg PTZ) + SEM. ** *p* < 0.01, *** *p* < 0.001, **** *p* < 0.0001 vs. the control group (Student’s *t*-test).

**Figure 3:**
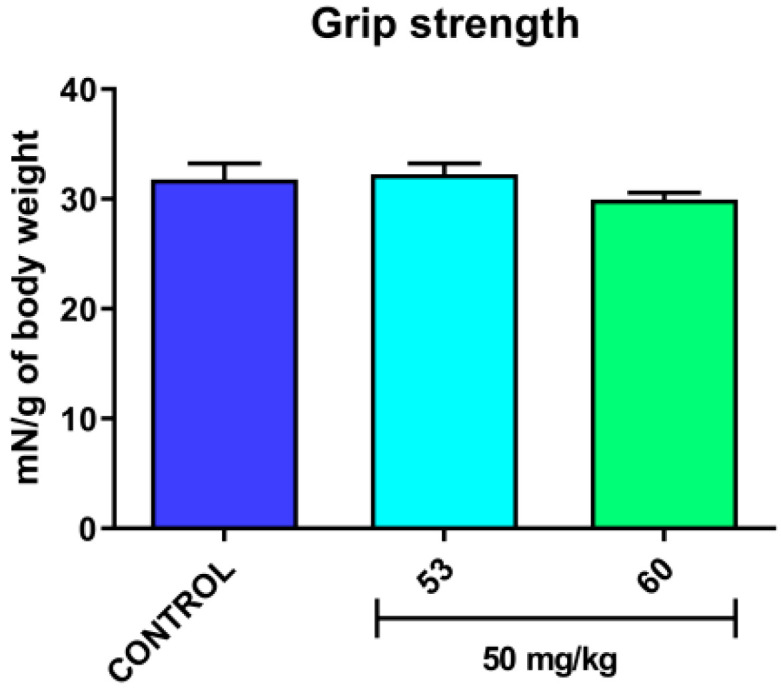
Acute effects of **53** and **60** on the neuromuscular strength in mice. Compounds *53* and **60** were administered *i.p.* 0.5 h before the seizure test. Control animals received vehicle only. Each experimental group consisted of 9–10 animals. Each bar represents the mean + SEM grip strengths in millinewtons per gram of mouse body weight (mN/g).

In studies assessing the acute effect of compounds **53** and **60** on the *ivPTZ* seizure threshold and neuromuscular strength, compounds **53** and **60** was suspended in a 1% solution of Tween 80 and administered *i.p.*, at a dose of 10 mL/kg body weight. Each experimental group consisted of 9–12 animals (*iv*PTZ seizure threshold test) or 9−10 animals (grip strength test). The timed *iv*PTZ test was employed to evaluate the acute effect of compounds **53** and **60** on the seizure thresholds for (1) the first myoclonic twitch, (2) generalized clonic seizure with loss of righting reflex, and (3) forelimb tonus. The experimental procedure of the timed *iv*PTZ test was described in detail elsewhere [50]. The acute effect of compounds **53** and **60** on neuromuscular strength was quantified using the grip-strength apparatus (BIOSEB, Chaville, France) according to the method that is described elsewhere [51]. The results are depicted in Figure 2 (*iv*PTZ seizure threshold test, see Section 3 Results and Discussion) and Figure 3 (grip strength test, see Section 3 Results and Discussion).

#### 2.3.4. Capsaicin-Induced Hypothermia Model in Mice

**Figure 4:**
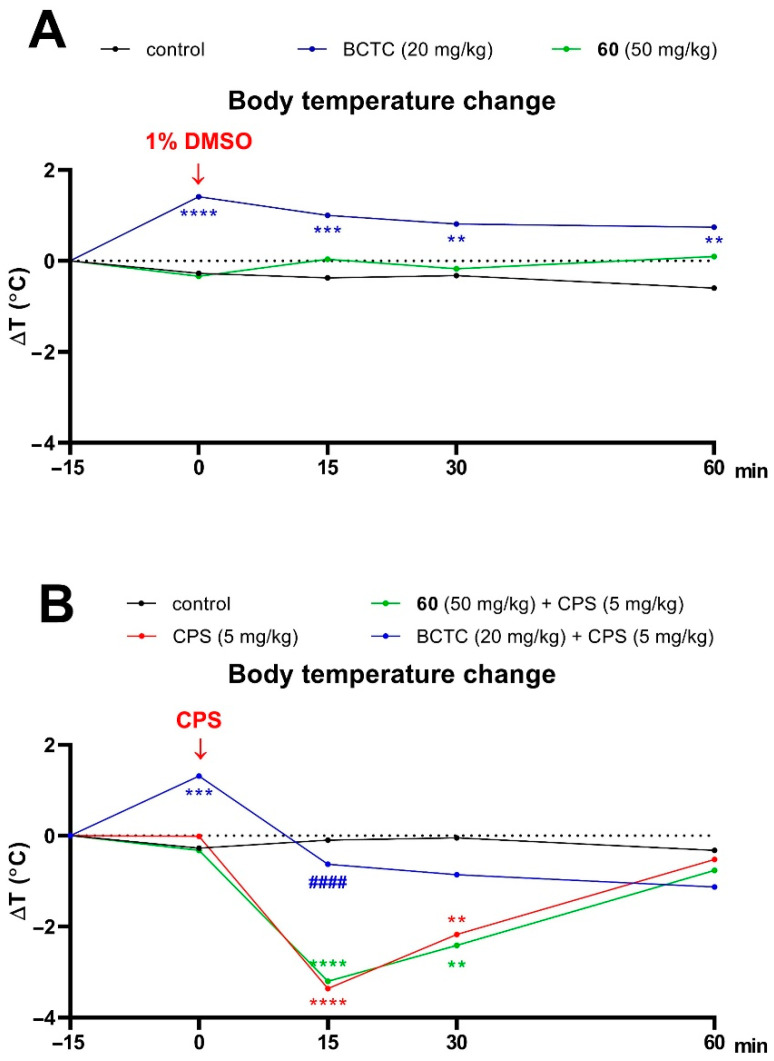
Effects of compound **60** and BCTC alone (**A**) or in combination with capsaicin (**B**) on the core body temperature in mice. Capsaicin (CPS) was injected at 0 min, as indicated by the arrow. Compound **60** or BCTC were administered 15 min prior to CPS injection. The temperature was measured at −15, 0, 15, 30, 60, 90, 120, and 180 min. Since body temperature returned to control values 60 min after CPS injection, subsequent measurements are not shown. Control animals received vehicles (1% DMSO or 1% Tween 80). All the compounds were administered *i.p*. Each experimental group consisted of seven to eight animals. The data are presented as the mean differences (ΔT) in rectal temperature from baseline (time −15 for **A** and time 0 for **B**) to the respective time point. The data were analyzed with one-way ANOVA followed by Tukey’s post hoc test. ** *p* < 0.01, *** *p* < 0.001, **** *p* < 0.0001 vs. the control group; ^####^ *p* < 0.0001 vs. the CPS-treated group.

Capsaicin was dissolved in 1% DMSO and administered at a dose of 5 mg/kg at a time point 0 min. Compound **60** and BCTC (positive control) were suspended in 1% Tween 80 and administered 15 min before capsaicin (at time point -15 min). All compounds were injected *i.p.* at a constant volume of 10 mL/kg. Control animals received the respective vehicles (1% DMSO or 1% Tween 80). Changes in the rectal temperature were measured using an electronic thermometer (ThermoWorks, Alpine, UT, USA) by inserting the rectal probe to a depth of ~2 cm into the rectum of the mouse. The measurements were taken at −15, 0, 15, 30, 60, 90, 120, and 180 min. The differences in the rectal temperature from baseline (time -15 min for groups receiving compound **60** and BCTC in combination with vehicle or time 0 min for groups receiving vehicle and capsaicin or compound **60** and BCTC in combination with capsaicin) to the respective time point ΔT (°C) were then calculated and analyzed. The results are shown in Figure 4 (see Section 3 Results and Discussion).

### 2.4. In Vitro ADME-Tox Studies

All assays and protocols that were used for the evaluation of compounds **53**, **60**, and **62** in the in vitro ADME-Tox studies were described previously [33,34,45,52,53,54]. Pre-coated PAMPA Plate System Gentest™ that was used in permeability testing was provided by Corning, (Tewksbury, MA, USA). The metabolic stability assay was performed on human liver microsomes (HLMs), purchased from Sigma-Aldrich (St. Louis, MO, USA). The most probable sites of compounds metabolism were estimated in silico by MetaSite 6.0.1 provided by Molecular Discovery Ltd. (Hertfordshire, UK). Influence on recombinant human CYP3A4, CYP2D6, and CYP2C9 isoforms of the P450 cytochromes (DDIs prediction) were carried out applying luminescent CYP3A4 P450-Glo™, CYP2D6 P450-Glo™, and CYP2C9 P450-Glo™ kits provided by Promega (Madison, WI, USA). Cell-based safety tests were performed with hepatoma HepG2 (ATCC^®^ HB-8065™) and neuroblastoma SH-SY5Y (ATCC^®^ CRL-2266™) cell lines that were obtained directly from ATCC^®^ (American Type Culture Collection, Manassas, VA, USA). The CellTiter 96^®^ AQueous Non-Radioactive Cell Proliferation Assay (MTS) that was used for the determination of cells viability was purchased from Promega (Madison, WI, USA). The luminescent signal and the absorbances (measured at 490 nm) in DDIs and safety assays were measured by using a microplate reader EnSpire PerkinElmer (Waltham, MA, USA). The LC/MS/MS analyses that were used in PAMPA and metabolic stability assays were obtained on Waters ACQUITY™ TQD system (Waters, Milford, CT, USA). All reference drugs that were used and other substances (i.e., ketoconazole, quinidine, sulfaphenazole, carbonyl cyanide 3-chlorophenyl- hydrazone (CCCP), doxorubicin, and verapamil) were purchased from Sigma-Aldrich (St. Louis, MO, USA).

### 2.5. In Vitro Electrophysiological Studies 

The methodology of slice preparation, slice preincubation, and patch-clamp technique was the same as in our previous studies [55]. Compound **53** was tested at a concentration of 10 µM.

### 2.6. Binding/Functional Studies

Binding/functional studies were carried out commercially in Eurofins Laboratories (Poitiers, France) and Eurofins Panlabs Discovery Services Taiwan, Ltd. (New Taipei City, Taiwan) using testing procedures that were reported previously (for details see Table S2).

## 3. Results and Discussion

### 3.1. In Silico Studies

All compounds were designed in line with physicochemical properties based on Lipinski and Veber rules using the SwissAdme software (Table 1) [46]. It should be emphasized that both the aforementioned rules are often used in medicinal chemistry to evaluate whether the molecule possesses drug-like physicochemical properties that are suitable for oral administration in humans [56].

**Table 1:** Drug-like parameters according to Lipinski rule, Veber rule, and CNS MPO.

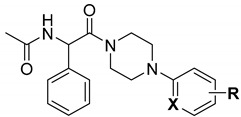										
Cmpd	X	R	Lipinski Rule				Violations of Rules	Veber Rule		CNS MPO ^e^
			MW ≤ 500	Log P ≤ 5	NHD ^a^ ≤ 5	NHA ^b^ ≤ 10		NBR ^c^ ≤ 10	TPSA ^d^ ≤ 140 Å^2^	
**45**	**C**	H	337.42	2.04	1	2	0	6	52.65	5.8
**46**	**C**	2-Cl	371.86	2.60	1	2	0	6	52.65	5.5
**47**	**C**	3-Cl	371.86	2.56	1	2	0	6	52.65	5.5
**48**	**C**	4-Cl	371.86	2.56	1	2	0	6	52.65	5.5
**49**	**C**	3,4-diCl	406.31	3.05	1	2	0	6	52.65	4.8
**50**	**C**	3,5-diCl	406.31	3.08	1	2	0	6	52.65	4.8
**51**	**C**	3-Cl,5- CF_3_	439.86	3.64	1	5	0	7	52.65	4.3
**52**	**C**	2-CF_3_	405.31	3.08	1	5	0	7	52.65	5.1
**53**	**C**	3-CF_3_	405.31	3.10	1	5	0	7	52.65	5.1
**54**	**C**	4-CF_3_	405.31	3.06	1	5	0	7	52.65	5.1
**55**	**C**	3,5-diCF_3_	473.41	4.10	1	8	0	8	52.65	3.8
**56**	**C**	3-CH_3_	351.44	2.36	1	2	0	6	52.65	5.6
**57**	**C**	3-CHF_2_	387.43	2.80	1	4	0	7	52.65	5.5
**58**	**C**	3-C_6_H_5_	413.51	3.35	1	2	0	7	52.65	4.3
**59**	**C**	3-OCH_3_	367.44	2.00	1	3	0	7	61.88	5.8
**60**	**C**	3-OCF_3_	421.41	2.91	1	6	0	8	61.88	4.5
**61**	**C**	3-OC_6_H_5_	429.51	3.18	1	3	0	8	61.88	4.4
**62**	**C**	3-SCF_3_	437.48	3.47	1	5	0	8	77.95	3.8
**63**	**N**	3-CF_3_	406.40	2.58	1	6	0	7	65.54	5.4
**64**	**N**	4-CF_3_	406.40	2.51	1	6	0	7	65.54	5.4
**65**	**N**	5-CF_3_	406.40	2.52	1	6	0	7	65.54	5.4
**66**	**N**	6-CF_3_	406.40	2.54	1	6	0	7	65.54	5.4

^a^ NHD–number of hydrogen bond donors, ^b^ NHA–number of hydrogen bond acceptors, ^c^ NBR–number of rotatable bonds, ^d^ TPSA–total polar surface area, ^e^ CNS MPO–Central Nervous System Multi-Parameter Optimization scores were calculated using the Instant JChem 21.4.0 software (ChemAxon).

Lipinski’s rule of five (RO5) assumes: molecular weight (MW) ≤ 500 Da, lipophilicity (log P) ≤ 5, number of hydrogen bond donors (NHD) ≤ 5, and the number of hydrogen bond acceptors (NHA) ≤ 10. Veber’s rule involves the number of rotatable bonds (NBR) ≤ 10 and polar surface area (TPSA) ≤ 140 Å^2^. As a result, all the designed compounds comply with RO5 and Veber rules. Moreover, all the substances meet the criteria of central nervous system multiparameter optimization (CNS MPO) according to Instant JChem by ChemAxon software version 21.4.0 (for details see Table 1). The CNS MPO score is now a well-recognized algorithm, which consisted of six key physicochemical properties: lipophilicity (ClogP); calculated distribution coefficient at pH 7.4 (ClogD); molecular weight (MW); topological polar surface area (TPSA); number of hydrogen-bond donors (HBDs); and most basic center (pKa). Each parameter has values between 0 and 1, thus the collective score range from 0 to 6 (a higher CNS MPO score is more desirable). The scores ≥ 4.0 are widely used as a cut-off to select compounds for hit finding in CNS therapeutic area drug discovery programs [57]. It should be stressed here that all the designed compounds had scores ≥ 3.8. Moreover, most of the compounds (**45**–**54**, **56**–**61**, and **63**–**66**) in the set had CNS MPO scores that were greater than 4.

### 3.2. Chemistry

The final compounds (**45**–**66**) were obtained applying the multi-step synthetic procedure that also involved the preparation of selected non-commercial amines (**A13**–**A24**). The non-commercial 4-arylpiperazines (**A13**–**A24**) were synthesized in a two-step reaction according to Scheme 1. First, intermediates **A1**–**A12** were obtained by a reaction of aryl bromides with 1-Boc-piperazine in the Buchwald–Hartwig amination reaction in the nitrogen atmosphere [58]. The removal of the Boc group in acid conditions (TFA) followed by neutralization with 25% ammonium hydroxide yielded the desired 4-arylpiperazine derivatives **A13**–**A24** which were used for further reactions without purification.

The final compounds **45**–**66** were synthesized according to Scheme 2. First, the condensation reaction of appropriate 4-arylpiperazine derivatives (commercial or non-commercial, **A13**–**A24**), with Boc-DL-phenylglycine in the presence of CDI yielded intermediates **1**–**22**. In the next step, as a result of removal of the Boc-protecting group by the addition of TFA, the amine derivatives (**23**–**44**) were obtained. The target compounds (**45**–**66**) were obtained in an acylation reaction of **23**–**44** by acetyl chloride. The crude products were purified by applying column chromatography. The desired compounds were obtained as white solids, followed by the concentration of organic solvents under reduced pressure and wash-up with diethyl ether.

Compounds **45**–**66** were obtained in good yields (>84%). The structures of non-commercial 4-phenylpiperazines and final molecules were confirmed by ^1^H NMR and/or ^13^C NMR spectra analyses. Moreover, for all compounds the LC-MS spectra were also obtained. The purity of **45**–**66** that was determined by UPLC method was ≥99%. The physicochemical and spectral data for the intermediates and final compounds are summarized in the Materials and Methods section.

### 3.3. Anticonvulsant Activity

The basic animal seizure models such as the MES, 6 Hz (32 mA or 44 mA), and the *sc*PTZ are still a standard in the discovery and development of new ASDs [59]. In vivo screening approaches enable the identification of compounds with unidentified and potentially novel mechanisms of action besides substances with well-established pharmacodynamics. Bearing in mind the aforementioned facts, compounds **45**–**66** were initially studied in the MES test and in the 6 Hz seizure model (32 mA) after intraperitoneal (*i*.*p*.) administration at a screening dose of 100 mg/kg in mice at a time point of 0.5 h (the screening group consisted of four mice, the results obtained are summarized in Table S1). It should be stressed that the MES test is an experimental model of human tonic–clonic epilepsy as well as partial convulsions with or without secondary generalization, the 6 Hz model (32 mA) corresponds to human focal epilepsy [60], whereas the *sc*PTZ test relates to human generalized absence or myoclonic seizures [61].

According to the MES screening data, 75% protection (three mice of four tested), was demonstrated for compounds **45**, **53**, **60**, **62**, and **65**. Other substances showed weak protection (25%)–**48** or lack of activity at a dose of 100 mg/kg. Thus, the highest activity in this test showed unsubstituted derivative **45** and compounds containing electron-withdrawing substituents in the phenylpiperazine moiety, namely **53** (3-CF_3_), **60** (3-OCF_3_), **62** (3-SCF_3_), or in the 1-(pyridin-2-yl)piperazine moiety–**65** (5-CF_3_).

Notably, distinctly more potent protection was observed in the 6 Hz (32 mA) test as only two compounds **59** and **63** were devoid of activity. Consequently, maximal 100% antiseizure protection was shown for **53** (3-CF_3_), and its bioisosteres, namely **60** (3-OCF_3_), and **62** (3-SCF_3_). Compounds **45**, **47**, **48**, and **65** provided 75% protection, whereas **46**, **52**, **54**, **58**, and **66** protected 50% of the animals. Compounds **49**–**51**, **55**–**57**, **61**, and **64** showed only weak protection (25%). According to the screening data, five compounds (**45**, **53**, **60**, **62**, **65**) were considered as most promising since they afforded at least 75% seizure protection in both MES and 6 Hz (32 mA) tests. With the aim of developing new and broad spectrum antiseizure drugs, all compounds that were effective in the MES and 6 Hz (32 mA) tests, namely **45**, **53**, **60**, **62**, **65** were further tested using the *sc*PTZ test. Unfortunately, none of the aforementioned compounds showed satisfactory activity in this test (only **53** and **60** provided weak 25% protection, Table S1). The lack or very weak activity in the aforementioned chemically-induced seizures proves that the pyrrolidine-2,5-dione ring seems to be crucial for activity in the *sc*PTZ test, as it was observed for structurally-related succinimide analogues that were described by our team in the previous studies [28].

Based on the initial screening data, it may be concluded that the most robust and broad spectrum antiseizure activity was observed with compounds containing electron withdrawing groups at the 3-position of the phenylpiperazine fragment, such as especially -CF_3_, -OCF_3_, and -SCF_3_. The replacement of these groups by bigger substituents (e.g., phenyl, phenoxy) or electron donating group (e.g., methyl) caused a decrease of activity. The introduction of Cl or CF_3_ substituents at the 2- or 4- position of the phenylpiperazine fragment or disubstitution at 3,4 or 3,5- positions of phenylpiperazine moiety also caused decrease of antiseizure activity. Finally, exchange of the benzene ring by pyridine moiety resulted in a strong decrease of antiseizure activity.

Continuing the in vivo screening, we performed similar assays for BCTC (selective TRPV1 antagonist), which was one of the chemical prototypes for compounds that were described in the current studies. BCTC was ineffective in all the tests where it was used, i.e., MES, 6 Hz (32 mA), and *sc*PTZ seizure models (see Table S1). Despite a good penetration of BCTC to the brain [44], it seems unlikely that selective blockade of TRPV1 channel may provide potent antiseizure protection in the aforementioned seizure models, as it was hypothesized previously [24,62]. Nevertheless, it cannot be completely excluded that such a mechanism of action may be beneficial in other seizure models, thus the hypothesis linking epilepsy and TRPV1 requires further and more detailed studies. Consequently, we assume herein that TRPV1 receptors may be interesting targets for new antiseizure drugs, but only as part of a more complex and complementary pharmacodynamic profile (i.e., inhibition of sodium or calcium conductance, etc.), as it was described for CBD [27].

In the next step of pharmacological investigations, we determined the median effective doses (ED_50_) for all the anticonvulsant compounds protecting at least 75% of mice in each seizure model (MES or/and 6 Hz [32 mA]) during the screening assessment. We also established the median toxic doses (TD_50_) in the chimney test 0.5 h post *i.p.* administration of these compounds. Both the aforementioned parameters enabled the calculation of the protective indexes (PIs). The results that were obtained for the compounds that are reported herein, and previously published data for ASDs with well-established clinical utility, such as levetiracetam (LEV, effective in the 6 Hz test [32 mA]); lacosamide (LCS, active in the MES and 6 Hz [32 mA] tests), and valproic acid (VPA), which is widely recognized as a broad-spectrum ASD (active in the MES, 6 Hz [32 mA], and *sc*PTZ seizure models) are summarized in Table 2. Moreover, we also included antiseizure activity results for CBD, which seems to have a similar in vivo and in vitro profile when compared to compounds that were described in the current paper.

**Table 2:** The ED_50_, TD_50_, and PI values in mice after *i.p.* dosing of the newly obtained compounds and reference ASDs in mice.

Cmpd	PT (h) ^a^	ED_50_ MES (mg/kg)	ED_50_ 6 Hz (32 mA) (mg/kg)	TD_50_ (mg/kg)	PI (TD_50_/ED_50_)
**45**	0.5	125.2 (105.3−148.9)	81.6 (61.8−107.8)	268.2 (242.0−297.1) *	2.1 (MES) 3.3 (6 Hz)
**47**	0.5	>150	88.4 (73.2−106.8)	>500 *	>5.6 (6 Hz)
**48**	0.5	>150	60.1 (44.9−80.6)	216.9 * (179.6−261.9)	3.6 (6 Hz)
**53**	**0.5**	**89.7** **(71.4−112.8)**	**29.9** **(20.1−44.4)**	179.7 * **(161.0−200.5)**	**2.0 (MES)** **6.0 (6 Hz, 32 mA)**
**60**	**0.5**	**73.6** **(63.6−85.2)**	**24.6** **(12.2−49.5)**	166.8 * **(109.6−253.8)**	**2.3 (MES)** **6.8 (6 Hz)**
**62**	**0.5**	**76.1** **(61.5−94.3)**	**33.2** **(21.2−52.0)**	156.2 * **(137.7−177.1)**	**2.1 (MES)** **4.7 (6 Hz)**
**65**	0.5	>100	64.9 (50.1−84.1)	177.8 * (166.8−190.4)	2.7 (6 Hz)
**KA-104 ^b^**	0.5	23.7 (18.4−31.2)	22.4 (17.4−28.8)	195.7 ** (132.7−288.6)	8.2 (MES), 8.7 (6 Hz)
**CBD ^c^**	1.0	80.0 (65.5−96.0)	144.0 (102.0−194.0)	272.0 ** (241.0−303.0)	3.4 (MES) 1.9 (6 Hz)
**LEV ^d^**	1.0	>500	15.7 (11.2−18.4)	>500 **	>31.8 (6 Hz)
**LCS ^d^**	0.5	9.2 (8.5−10.0)	5.3 (3.5−7.8)	46.2 ** (44.5−48.0)	5.0 (MES) 8.8 (6 Hz)
**VPA ^d^**	0.5	252.7 (220.1−290.2)	130.6 (117.6−145.2)	430.7 ** (407.9−454.9)	1.7 (MES) 3.3 (6 Hz)

The data for the most potent compounds **53**, **60**, and **62** have been bolded for better visualization. Results are represented as mean ± SEM at 95% confidence limit determined by probit analysis [63]. Acute neurological deficit (TD_50_) determined in the * chimney test or the ** rotarod test. ^a^ Pretreatment time. ^b^ Data for **KA-104**, see compound **22** in ref. [32]. ^c^ Data for cannabidiol (CBD) from [64]. ^d^ Reference ASDs: Levetiracetam (LEV), Lacosamide (LCS), and Valproic acid (VPA) tested in the same conditions data from [32].

As expected, on the basis of screening data, compounds **53** (3-CF_3_), **60** (3-OCF_3_), and **62** (3-SCF_3_) displayed the most potent protection in the MES and 6 Hz (32 mA) seizure tests, whereas weaker activity in both the mentioned seizure models was observed for **45**. Other substances (**47**, **48**, and **65**) were effective exclusively in the 6 Hz (32 mA) seizure model. Among the aforementioned compounds, the most promising anticonvulsant and safety profile revealed compounds **53** and **60** which showed a slightly better therapeutic window (expressed as PI values) than their close analogue **62** (3-SCF_3_). The chemical prototype compound–**KA-104** showed higher protection in the MES test, whereas activity in the 6 Hz (32 mA) seizure model remained at a similar level. Notably, **53** and **60** demonstrated distinctly more potent anticonvulsant activity in the MES and 6 Hz (32 mA) tests and also showed better PIs in each seizure model than that of VPA, which is still recognized as one of the most frequently prescribed first-line ASD in different types of epilepsies [65]. Unfortunately, compounds **53** and **60** exhibited lower potency and safety margins compared to LCS, and especially LEV, which is recognized as the reference ASD effective in the 6 Hz (32 mA) seizure model. Importantly, compounds **53** and **60** showed similar activity in the MES test and revealed distinctly more potent protection in the 6 Hz (32 mA) seizure model in comparison to CBD, which is one of the newest ASDs with unique and multi-target mechanism of action as described above. 

Taking into consideration the potent protection of **53** and **60** in the 6 Hz (32 mA) seizure model, these compounds were also evaluated by applying a higher current intensity of 44 mA. It should be emphasized that the 6 Hz (44 mA) seizure model is recognized as the basic animal model of human DRE, utilized in early stage of new ASDs development [66]. 

The data that were obtained (Table 3) revealed relatively potent protection for both tested compounds (**53** and **60**), and slightly more potent activity was noted for **60**, which also demonstrated the best antiseizure properties in the 6 Hz (32 mA) test. Additionally, the chemical imide prototype for these compounds, **KA-104**, showed a slightly weaker protection. It should be stressed herein that both **53** and **60** were more effective and possessed more beneficial PI values than VPA and CBD. It is noteworthy that in this seizure model LEV, acting on SV2A protein located in the presynaptic vesicle membranes, was distinctly less potent. Importantly, LCS which increases the slow inactivation of sodium channels, revealed an excellent efficacy in this seizure model.

In summary, the most potent antiseizure compounds, namely **53** and **60** showed a wide spectrum of protection and were effective in the MES, 6 Hz (32 mA) models, and notably the 6 Hz (44 mA) seizure model of DRE. The applied structural modification that relied on the exchange of the succinimide ring, which is present in the structure of compounds that were described previously (represented by chemical predecessor **KA-104 [32,34]**), to acetyl fragment slightly decreased the activity of compounds that were reported herein, especially in the MES test and caused a loss of protection in the *sc*PTZ test. Importantly, the activity in the 6 Hz (32 mA) seizure model and 6 Hz (44 mA) model of DRE remained at a similar level. Ultimately, it should be emphasized that both **53** and **60** showed distinctly more potent protection in the 6 Hz (32/44 mA) models, as well as similar or more potent effects in the MES test when compared to VPA and CBD.

### 3.4. Effect on the Seizure Threshold in the ivPTZ Test in Mice

The timed *iv*PTZ test was employed to further evaluate the effects of compounds **53** and **60** on seizure susceptibility in mice. The *iv*PTZ seizure test is an extremely sensitive method for assessing seizure thresholds in rodents. In this method, the threshold PTZ dose for several seizure endpoints was determined [67]. The obtained results showed that **53** administered at the dose of 50 mg/kg significantly increased the threshold for the first myoclonic twitch (*p* < 0.0001) and generalized clonus with loss of righting reflex (*p* < 0.01), by ~50% and ~40%, respectively. The compound did not, however, produce any significant effect on the threshold for the forelimb tonus. Compound **60** at the dose of 50 mg/kg raised the threshold for the first myoclonic twitch by ~20% (*p* < 0.001), but it was devoid of any significant effects on the PTZ-induced seizure susceptibility for both generalized clonic seizure and forelimb tonic extension. Altogether, **53** was more active in the *iv*PTZ test than **60** (Figure 2).

**Figure 5:**
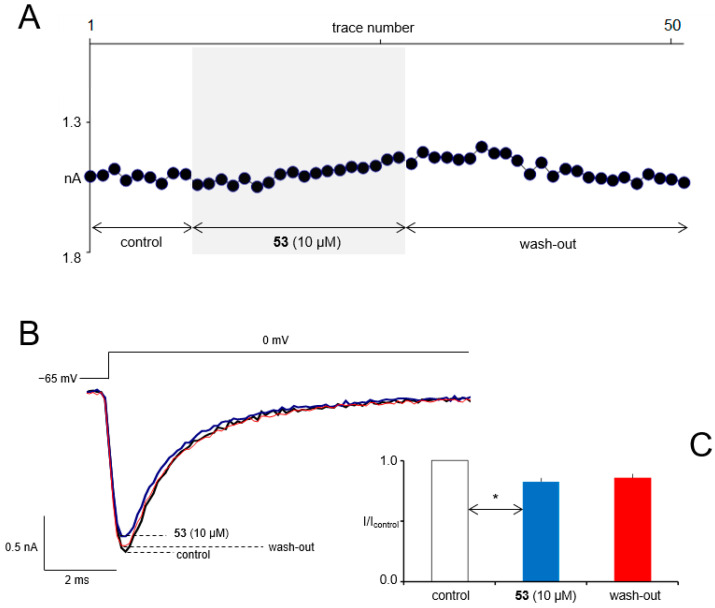
(**A**) The influence of compound **53** on sodium current is shown on an example neuron. The current was evoked once every ten seconds by a rectangular voltage-step. Vertical axis shows the maximal current amplitudes (black circles) in the control, in the presence of **53,** and after wash-out. The horizontal axis shows the trace number. (**B**) Example of the sodium current recordings in the control (black trace), after the application of the tested compound (blue trace), and after wash-out (red trace). (**C**) The averaged normalized maximal current amplitudes in the control, in the presence of **53,** and after wash-out. The data were analyzed with a nonparametric repeated measures ANOVA followed by Dunn’s post hoc test. * *p* < 0.01 (*n* = 7).

Although both **53** and **60** were effective against MES-induced tonic seizures, they did not produce any significant effects on forelimb tonus in the *iv*PTZ. The differential effect of **53** and **60** on tonic seizures could be related to distinct mechanisms underlying seizure activity in the *iv*PTZ and MES tests. The proconvulsant activity of PTZ is, at least partially, mediated by its ability to act as a blocker of the picrotoxin site of the chloride ionophore of the GABA_A_ receptor complex. Consequently, drugs affecting the seizure threshold in the *iv*PTZ test are generally considered to act through GABA-mediated mechanisms, whereas the MES test is thought to be useful for detecting agents that block sodium channels [67,68]. Thus, **53** and **60** appear to inhibit tonic seizures rather by blocking sodium channels than by GABA-ergic mechanisms. Indeed, **53** and **60** were shown to interact with sodium channels, but not with the GABA_A_ receptor, in in vitro binding studies (Table 4 and Table 5). In addition, **53** inhibited the maximal amplitude of sodium currents rat prefrontal cortex pyramidal neurons (Figure 5). On the other hand, **53** and **60** elevated the threshold for the first myoclonic twitch in the *iv*PTZ test. Myoclonic seizure is generally considered to result from alterations in GABA_A_ receptor activity along the neural axis [69], which suggests that **53** and **60** may interact with GABA-mediated neurotransmission. However, the mechanism underlying myoclonus is not fully understood. Likewise, the exact mechanism of proconvulsant activity of PTZ remains to be established.

### 3.5. Acute Effect on the Neuromuscular Strength in Mice

Compounds **53** and **60** that were injected at the dose of 50 mg/kg did not significantly affect the neuromuscular strength as assessed in the grip strength test (Figure 3).

### 3.6. Effect on the Capsaicin-Induced Hypothermia in Mice

The pharmacological blockade of TRPV1 often elicits marked hyperthermia and this is one of the most common adverse effects that is encountered in preclinical and clinical studies using first-generation TRPV1 antagonists. The first generation TRPV1 antagonists are polymodal and block all three TRPV1 activation modes (i.e., by capsaicin, low pH (protons), and heat), whereas second generation TRPV1 antagonists are mode-specific–they block channel activation by capsaicin but exert differential effects on other modes of TRPV1 activation (potentiation, lack of effect, or low-potency inhibition) [70,71]. It appears that the influence of TRPV1 antagonists on body temperature depends on the TRPV1 activation mode. Compounds that inhibit all three modes of TRPV1 channel activation are generally expected to increase body temperature, while second generation TRPV1 antagonists that act mode-specifically may be devoid of the hyperthermia-inducing effects [72]. In rodents, only activation by protons is involved in the thermoregulatory response to TRPV1 antagonists and this response is completely insensitive to the antagonist’s potency to block either capsaicin or heat activation of TRPV1 [71].

In view of the above, we aimed to gain more insight into the TRPV1-mediated effects of compound **60** by evaluating its influence on the capsaicin-induced hypothermia in mice (Figure 4). In this experiment, capsaicin that was administered at a single dose of 5 mg/kg produced a significant drop in the rectal temperature at 15 min and 30 min following administration (*p* < 0.0001 and *p* < 0.01 vs. control group, respectively). The body temperature returned to control values 60 min after capsaicin injection. BCTC (a positive control) that was administered alone at the dose of 20 mg/kg significantly increased the temperature at time points: 0 min (*p* < 0.0001); 15 min (*p* < 0.001); 30, 60, 120 min (*p* < 0.001); and 180 min (*p* < 0.05). When co-injected with capsaicin, BCTC prevented the capsaicin-evoked decrease in body temperature. Compound **60** given alone at the dose of 50 mg/kg did not cause any significant changes in the body temperature. Also, the co-administration of compound **60** with capsaicin did not affect the capsaicin-induced hypothermia. These findings suggest that compound **60** may be a hyperthermia-free TRPV1 antagonist that does not work by the proton mode. However, the lack of ability of compound **60** to reverse the capsaicin-induced hypothermia was more likely related to its moderate TRPV1 antagonistic activity. 

### 3.7. In Vitro Radioligand Binding Studies and Functional Assays

Despite a plethora of advanced in vitro assays (binding, functional, biochemical, etc.) which enable the elucidation of mechanism of action for drug candidates, the development of new ASDs is still based on predictable animal seizure models as the first line of the discovery process [73]. Moreover, it should be also emphasized that the precise mechanism of action for the majority of ASDs that are currently used in treatment (e.g., LEV or LCS) was elucidated years after their market authorization.

It is widely recognized that both sodium and calcium channels are important molecular targets for several structurally diverse ASDs such as LCS, lamotrigine, carbamazepine, oxcarbazepine, etc. [74]. Consequently, for the most active compounds that were identified in the in vivo studies, namely **53**, **60**, and **62,** their binding profile toward sodium channel (site 2) and calcium Cav_1.2_ channel at concentrations of 10 μM was assessed in vitro. It should be emphasized here that numerous neurobiological studies which have been performed in recent years that have proven that dysfunction of Cav_1.2_ calcium ion channels may be involved in the pathogenesis of epilepsy [75,76] and neuropathic pain [37,38]. As it is shown in Table 4, the tested compounds, despite strong structural similarities, revealed different affinity for sodium channels, i.e., the most potent binding at a concentration of 10 µM was observed for 3-SCF_3_ derivative **62**, the 3-OCF_3_ analogue (**60**), while the weakest effect was displayed by the 3-CF_3_ congener (**53**). Moreover, none of the compounds that were tested showed a significant effect on Cav_1.2_ channel (dihydropyridine site) in the binding assays. However, it should be emphasized that in the functional assays, two compounds (**53** and **62**) revealed potent inhibition of calcium currents that were mediated by the aforementioned calcium channels. This may indicate that **53** and **62** bind to the Cav_1.2_ channel in a different site than that of dihydropyridine derivatives. The binding assays that were performed with CBD and **53** at the concentration of 100 μM revealed a significant interaction with the sodium channels. Moreover, CBD also exerted a significant effect on the Cav_1.2_ channel (at 100 μM). These in vitro studies may indicate that modulation of sodium and calcium currents by compounds **53**, **60**, and **62** may potentially contribute to their broad antiseizure activity in vivo.

**Table 4:** In vitro binding and functional assays for **53**, **60**, **62**, and **CBD**.

Binding Studies	Source	% Inhibition of Control Specific Binding (Concentration [µM]) ^a^			
		53	60	62	CBD
Na^+^ channel (site 2)	Rat cerebral cortex	75.5 (100) 17.4 (10)	37.0 (10)	57.0 (10)	94.8 (100)
Calcium Cav_1.2_ channels (dihydropyridine site antagonist radioligand)	Human recombinant HEK-293 cell	4.0 (10)	7.0 (10)	12.0 (10)	58.0 (100)
**Functional studies**	Source	% Inhibition of control agonist response (concentration [µM]) ^a^			
		**53**	**60**	**62**	**CDB**
Cav_1.2_ (h) calcium ion channel cell-based antagonist calcium flux assay	Human recombinant HEK-293 cell	55.0 (10)	31.0 (10)	65.0 (10)	NT
TRPV1 (VR1) (h) (antagonist effect)	Human recombinant CHO cells	128.5 (100) IC_50_ = 13 μM, K_B_ = 1.7 μM	109.7 (100) IC_50_ = 11 μM, K_B_ = 1.5 μM	135.1 (100) IC_50_ = 10 μM, K_B_ = 1.4 μM	48.0 (100)

^a^**Results showing activity higher than 50% are considered to represent significant effects of the test compounds**; results showing an inhibition between 25% and 50% are indicative of moderate effect; and results showing an inhibition lower than 25% are not considered significant and mostly attributable to variability of the sign al around the control level. NT–not tested. Cannabidiol (CBD).

According to the concept of structural hybridization, compounds that were described in the current paper were designed as analogues of known TRPV1 antagonists, e.g., BCTC and JNJ-17203212 (Figure 1). Therefore, in the next step of the in vitro studies we determined the TRPV1 receptor antagonist activity for compounds **53**, **60**, and **62** that were characterized by the most potent antiseizure activity. The results of functional assays (Table 4) confirmed the TRPV1 antagonist activity for compound **53** (IC_50_ = 13 μM, K_B_ = 1.7 μM), **60** (IC_50_ = 11 μM, K_B_ = 1.5 μM), and **62** (IC_50_ = 10 μM, K_B_ = 1.4 μM). Interestingly, CBD showed weaker, nevertheless significant, TRPV1 channel antagonism compared to compounds **53**, **60**, and **62**. Therefore, it is justified to postulate that the compounds that were reported herein seem to have a similar and multi-target pharmacodynamic profile as CBD, at least when it comes to the modulation of the above-mentioned ion channels. Furthermore, this assumption is additionally supported by a similar anticonvulsant profile that was obtained in the in vivo studies (see Table 2 and Table 3).

With the aim of confirming the influence of the TRPV1 blockade on the antiseizure activity of the compounds that were described herein, we compared the interaction of most potent anticonvulsants (**45**, **47**, **48**, **53**, **60**, **62**, and **65**) vs. weakly active and inactive (**46**, **49**−**52**, **54**−**59**, **61**, **63**, **64**, and **66**) compounds with the mentioned molecular target (Table S3). As a result, there was no clear correlation between the TRPV1 antagonist activity and antiseizure efficacy. Thus, we suggest that TRPV1 may be a promising molecular target for new ASD-candidates but rather as a part of more complex, complementary, and multimodal pharmacodynamics. Furthermore, as TRPV1 channels are involved especially in nociception, such a mechanistic component could be beneficial in the designing of new ASDs with pronounced analgesic activity and additional pain indications, as it was proven for CBD which is known to possess potent analgesic activity in various animal neuropathic pain models [77,78]. Notably, we are going to confirm these assumptions in further ‘proof of concept’ studies. Nevertheless, as stated above, it is possible that selective TRPV1 antagonists may be effective in particular seizure models that were not utilized in the current studies.

In order to confirm or exclude additional molecular targets for substances that were described herein, **53** which was characterized by potent antiseizure activity was tested for interaction with other ion channels and GABA transporter which are known as the most common molecular targets for currently available ASDs (Table 5). Additionally, we also assessed the influence of **53** on the potassium channel (hERG) which is known to be one of the most crucial ‘off-targets’ that is responsible for harmful proarrhythmic activity of drugs and drug-candidates [79]. As a result, **53** did not interact with the NMDA receptor, N-type calcium channel and GABA_A_ receptor, GABA transporter, and notably hERG channel at concentration of 100 μM.

**Table 5:** Additional in vitro binding assays for **53**.

Binding Studies	Source	% Inhibition of Control Specific Binding (Concentration [µM]) ^a^
NMDA (antagonist radioligand)	Rat cerebral cortex	**9.0** (100)
N-type Ca^2+^ (antagonist radioligand)	Rat cerebral cortex	**1.5** (100)
GABA transporter (antagonist radioligand)	Rat cerebral cortex	**2.8** (100)
GABA_A_ ion channel [^3^H]GABA (agonist radioligand)	Rat cerebral cortex	**−1.1** (100)
Potassium channel (hERG)	Human recombinant HEK-293 cell	**24.0** (100)

^a^ Results showing activity higher than 50% are considered to represent significant effects of the test compounds; results showing an inhibition between 25% and 50% are indicative of weak effect; and results showing an inhibition lower than 25% are not considered significant and mostly attributable to variability of the signal around the control level. Binding studies were performed commercially in Eurofins Laboratories (Poitiers, France). All assays were performed in duplicate.

In summary, on the basis of functional data, it may be concluded that the hybrid compounds that are described herein are characterized by a multimodal mechanism of action which involves interaction with voltage-gated sodium (site 2), Cav_1.2_, and TRPV1 channels. It is suggested that the influence on the aforementioned and complementary molecular targets may decide about potent and broad-spectrum anticonvulsant activity that was observed for **53**, **60**, and **62**. Furthermore, the in vitro binding/functional profile of these molecules may indicate their potential antinociceptive properties. Notably, the antinociceptive studies are currently in progress and the results will be published soon.

### 3.8. In Vitro Electrophysiological Studies

The activity of compound **53** in the electrically-induced seizure models (e.g., MES and 6 Hz [32 mA and 44 mA]), as well as the results of binding studies suggest its influence on neuronal sodium conductance. Thus, we determined the influence of **53** on fast voltage-gated sodium channels in rat prefrontal cortex pyramidal neurons (at a concentration of 10 μM) using the patch-clamp technique [55]. Maximal currents were evoked by rectangular voltage steps to 0 mV. We found that the tested compound inhibited the maximal amplitude of sodium currents. The effect was not strong, but statistically significant (1.0 in the control and 0.83 ± 0.03 after the application of **53**, *p* < 0.01). It was possible to obtain partial wash-out (0.86 ± 0.03 and the current were normalized to the control level, *n* = 7, Figure 5).

### 3.9. In Vitro ADME-Tox Assays

Several ADME-Tox in vitro tests were done to assess the drug-like properties of compounds **53**, **60**, and **62**. The performed assays included PAMPA permeability, metabolic stability with use of human liver microsomes (HLMs) that were supported by the in silico studies, investigation of potential drug-drug interactions (DDIs), and safety cell-based tests. All the used protocols were described previously [33,34,45,52,53,54].

Pre-coated PAMPA Plate System Gentest™ (Corning, Tewksbury, MA, USA) was applied for the determination of the ability of the promising compound **60** to passive diffusion through cellular membranes. Caffeine (CFN) and norfloxacin (NFX) were used as the high- and low-permeable references, respectively. According to calculated permeability coefficient *Pe*, the tested compound showed very good permeability, close to that of CFN and the previously published compound **KA-104** [34] (Table 6).

In order to determine their metabolic stability, compounds **53**, **60**, and **62** were incubated for 120 min with HLMs. The obtained results were summarized in Table 7 and compared to the metabolically unstable drug verapamil. In general, all the tested compounds were found to be stable, as their calculated % on the basis of UPLC results remaining in the reaction mixture were higher than 80%, whereas this value determined for verapamil was only around 30% [81,82,83] (Table 7, Figures S2, S4, S6, and S8). Moreover, the performed MS/MS spectra that was supported by in silico results allowed for the determination of the metabolic pathways and the most probable structures of metabolites (Table 7, Figures S1, S3, S5, and S7).

**Figure 6:**
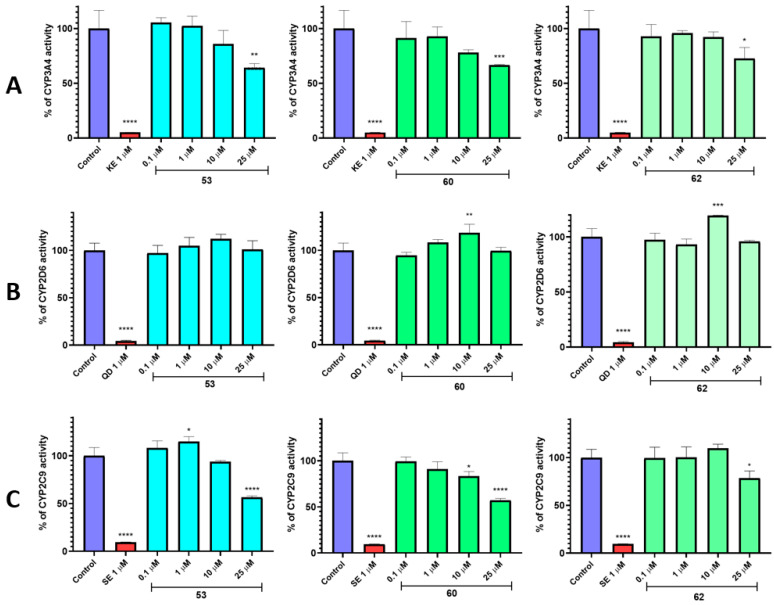
The influence of **53**, **60**, and **62** on: CYP3A4 activity (**A**) and the reference inhibitor ketoconazole (KE); CYP2D6 activity (**B**) and the reference inhibitor quinidine (QD); CYP2C9 activity (**C**) and the reference inhibitor sulfaphenazole (SE). The data were analyzed with one-way ANOVA, followed by Bonferroni’s Multiple Comparison Post Test (Graph Pad Prism 8.0.1 software, San Diego, CA, USA)): * *p* < 0.05, ** *p* < 0.01, *** *p* < 0.001 **** *p* < 0.0001. The compounds were examined in triplicate.

The risk of potential DDIs was predicted with use of luminescent CYP3A4, 2D6, and 2C9 P450-Glo assays (Figure 6). All the tested compounds statistically significantly inhibited the most important CYP3A4 isoform only at the highest used concentration of 25 µM, whereas the selective inhibitor ketoconazole decreased its activity almost completely at 1 µM (Figure 6A). Regarding CYP2D6, weak activation of that isoform was observed at 10 µM (Figure 6B). However, such an effect was also observed in our previous studies for other pyrrolidine-2,5-dione derivatives [32,34,52,84]. In the case of CYP2C9, similar to CYP3A4, a weak inhibition effect was visible for **53** and **62**, whereas a little stronger influence, observed also at 10 µM, was found for **60**. However, the potential risk of DDI of **60** was still assessed as very low, and was compared to the selective CYP2C9 inhibitor sulfaphenazole (Figure 6C).

**Figure 7:**
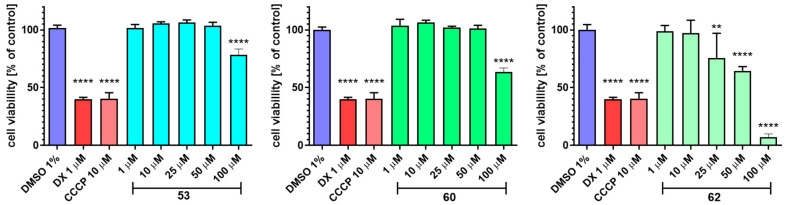
The effect of **53**, **60**, and **62**, cytostatic drug doxorubicin (DX) and the mitochondrial toxin CCCP on the hepatoma HepG2 cell line viability after 72 h of incubation at 37 °C, 5% CO_2_. The data were analyzed with one-way ANOVA, followed by Bonferroni’s Comparison Test (Graph Pad Prism 8.0.1 software, San Diego, CA, USA)): ** *p* < 0.01, **** *p* < 0.0001 vs. negative control (DMSO 1% in growth media).

The hepatotoxicity testing with the use of the HepG2 cell line showed compounds **53** and **60** as generally safe. The statistically significant effect on the cell viability after 72 h treatment was observed only at 100 µM. These results are in agreement with those that were obtained for **KA-104 [34]**. The highest hepatotoxic risk was determined for the sulfur-containing compound **62**. However, the toxic effect was still much weaker than that which was observed for the reference toxins i.e., doxorubicin (DX), and mitochondrial toxin-CCCP (Figure 7).

**Figure 8:**
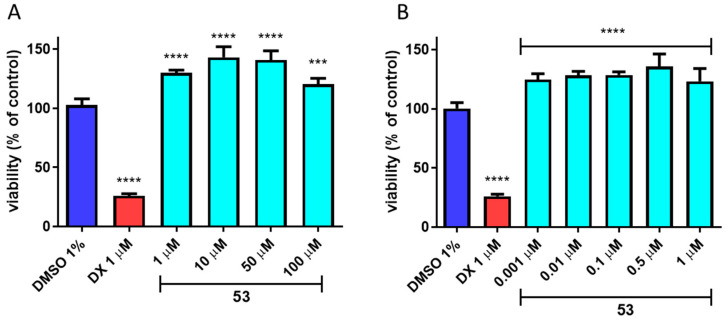
The effect of **53** and the cytostatic drug doxorubicin (DX) on neuroblastoma SH-SY5Y cell line viability after 72 h of incubation at 37 °C, 5% CO_2_ in the concentration range 1–100 µM (**A**) and 0.001–1 µM (**B**). The data were analyzed with one-way ANOVA, followed by Bonferroni’s Comparison Test (Graph Pad Prism 8.0.1 software, San Diego, CA, USA)): *** *p* < 0.001, **** *p* < 0.0001 vs. negative control (DMSO 1% in growth media).

The preliminary neurotoxicity in vitro tests that were performed for the selected compound **53** showed its stimulating effect on neuroblastoma SH-SY5Y cells in all the tested concentrations (Figure 8A). Moreover, the dose-dependent increase in the cells viability was found during the repeated experiment in lower concentrations (Figure 8B). The observed induction effect was even up to 140% of the control at the doses 10 and 50 µM. Interestingly, similar results were also found previously for another pyrrolidine-2,5-dione derivative from our library after incubation with astrocytes [85]. Thus, the observed influence of this chemical group for neuronal cells is worth further detailed exploration.

## 4. Conclusions

In the present study, utilizing focused combinatorial chemistry, we obtained a series of 22 chemically original compounds which possess a wide spectrum of activity in the preclinical in vitro and in vivo tests. They were effective in the most widely employed animal seizure models, i.e., the maximal electroshock (MES) test, the psychomotor 6 Hz (32 mA) seizure model, and importantly also in the 6 Hz (44 mA) model of drug-resistant epilepsy. The most potent compounds, **53** and **60,** were also effective in the *iv*PTZ seizure threshold test and did not affect the neuromuscular strength and body temperature in mice. The mechanism of action of the aforementioned molecules is likely multimodal and involves TRPV1 antagonism as well as inhibition of sodium and calcium currents. Furthermore, in vitro studies indicated beneficial ADME-Tox properties, making them interesting candidates for further preclinical development.

In the next steps of pharmacological characterization, we will determine the effect of **53** and/or **60** in a chronic PTZ-kindling model as well as animal models of pain (including models of neuropathy).

In summary, we postulate that the data that are described herein, and more detailed pharmacological characterization that we plan to perform in the future, will provide support for the development of **53** and **60** as novel epilepsy therapeutics with potential for neuropathic pain indications.

## Figures and Tables

**Table 3:** Effect of the synthesized compounds and reference ASDs that were administered *i.p.* in the 6 Hz (44 mA) seizures in mice.

Cmpd	PT (h) ^a^	ED_50_ 6 Hz (44 mA) (mg/kg)	TD_50_ (mg/kg)	PI (TD_50_/ED_50_)
**53**	0.5	68.0 (57.2–80.9)	179.7 * (161.0−200.5)	2.6
**60**	0.5	56.3 (46.8–67.7)	156.2 * (137.7−177.1)	2.8
**KA-104 ^b^**	0.5	73.2 (57.4–93.4)	195.7 ** (132.7–288.6)	2.7
**CBD ^c^**	1	173.0 (136–213)	272.0 ** (241.0−303.0)	1.6
**LEV ^d^**	0.5	204.0 (154.5–269.5)	>500 **	>2.5
**LCS ^d^**	0.5	6.9 (5.4–8.6)	46.2 ** (44.5–48.0)	6.7
**VPA ^d^**	0.5	183.1 (143.5–233.7)	430.7 ** (407.9−454.9)	2.3

The results are represented as the mean ± SEM at 95% confidence limit determined by probit analysis [63]. Acute neurological deficit (TD_50_) that were determined in the * chimney test or the ** rotarod test. ^a^ Pretreatment time. ^b^ Data for **KA-104** in ref. [34]. ^c^ Data for cannabidiol (CBD) from [64]. ^d^ Reference ASDs: Levetiracetam (LEV), Lacosamide (LCS), and Valproic acid (VPA) tested in the same conditions, data from [34].

**Table 6:** The results that were obtained in PAMPA test.

Comp	*Pe* * (10^−6^ cm/s) ± SD
CFN	12.22 ± 0.94
NFX	0.056 ± 0.01
**60**	**9.15 ± 0.78**

* The permeability coefficient (*Pe*) values of the compounds with high permeability are larger than 1.5 × 10^−6^ cm/s [80].

**Table 7:** Metabolic stability/biotransformation pathways after incubation with HLMs.

Substrate	Molecular Mass (*m/z*)	% Remaining	Molecular Mass of the Metabolite (*m/z*)	Metabolic Pathway *
**53**	406.28	95.59	422.30 (M1)	***hydroxylation***
**60**	422.30	88.80	438.25 (M1)	***hydroxylation***
			420.24 (M2)	*dehydrogenation*
**62**	438.25	84.01	454.26 (M1)	***hydroxylation***
			454.26 (M2)	*hydroxylation*
Verapamil **	455.31	30.84	441.35 (M1)	***demethylation***
			291.328 (M2)	*defragmentation*
			165.09 (M3)	*defragmentation*
			441.29 (M4)	*demethylation*
			427.33 (M5)	*double-demethylation*
			277.26 (M6)	*defragmentation*

* Main metabolic pathways have been bolded. ** Data for verapamil were previously reported [83].

## Data Availability

Not applicable.

## Supplementary Materials

The following are available online at https://www.mdpi.com/article/10.3390/cells11121862/s1. References [86,87,88,89,90,91,92,93,94,95] are cited in the Supplementary Materials. Figure S1: The MetaSite 6.0.1. software prediction of the most probably sites of tested compounds metabolism; Figure S2: UPLC spectra after 120 min incubation of compound **53** with human liver microsomes; Figure S3: MS ion fragment analyses and the most prob-able structure of 53 metabolite M1; Figure S4: UPLC spectra after 120 min incubation of compound **60** with human liver microsomes; Figure S5: MS ion fragment analyses and the most probable structure of 60 metabolites M1–M2; Figure S6: UPLC spectra after 120 min incubation of com-pound 62 with human liver microsomes; Figure S7: MS ion fragment analyses and the most prob-able structure of 62 metabolites M1–M2; Figure S8: UPLC spectra after 120 min incubation of the reference drug verapamil with human liver microsomes; Table S1: Anticonvulsant activity screening data for compounds **45**–**66** and BCTC in mice *i.p.*; Table S2: Radioligand binding and functional assays; Table S3: In vitro functional assays of compounds **45**–**66** for TRPV1 channel (concentration 100 μM).

## Abbreviations

ADME-Tox: absorption, distribution, metabolism, excretion, toxicity; ASDs, antiseizure drugs; BINAP, (±)-2,2′-Bis(diphenylphosphino)-1,1′-binaphthalene; CBD, cannabidiol; CDI, carbonyldi- -imidazole; CNS, central nervous system; DCM, dichloromethane; DDIs, drug-drug interactions; DRE, drug resistant epilepsy; DX, doxorubicin; GABA, gamma-aminobutyric acid; HLMs, human liver microsomes; 6 Hz, six-Hertz seizure test; LCS, Lacosamide; LEV, Levetiracetam; MeCN, acetonitrile; MES, maximal electroshock seizure test; MeOH, methanol; Pd_2_dba_3_, Tris(dibenzylideneacetone)dipalladium(0); PI, protective index (TD_50_/ED_50_); PTZ, pentylene- -tetrazole; *sc*PTZ, subcutaneous pentylenetetrazole seizure test; TFA, trifluoroacetic acid; TRPV1, transient receptor potential cation channel vanilloid type 1; VGSCs, voltage-gated sodium channels; VPA, Valproic acid.
