# Supplementary material for: New Phenylglycinamide Derivatives with Hybrid Structure as Candidates for New Broad-Spectrum Anticonvulsants
Source: Cells. 2022 Jun 7;11(12):1862. doi: 10.3390/cells11121862 (PMC9221546; doi:10.3390/cells11121862)

Supporting Information

New phenyl-glycinamide derivatives with hybrid structure as candidates on new effective anticonvulsants

Marcin Jakubiec [1], Michał Abram [1], Mirosław Zagaja [2], Marta Andres-Mach [2], Aleksandra Szewczyk [2], Gniewomir Latacz [3], Bartłomiej Szulczyk [4], Katarzyna Socała [5], Dorota Nieoczym [5], Piotr Wlaź [5], Cameron Metcalf [5], Karen Wilcox [5], Rafał M. Kamiński [1] and Krzysztof Kamiński [1]

[1] Department of Medicinal Chemistry, Faculty of Pharmacy, Jagiellonian University Medical College, Medyczna 9, 30-688 Krakow, Poland; marcin.jakubiec@doctoral.uj.edu.pl (M.J.); michal.abram@uj.edu.pl (M.A.); rafa1.kaminski@uj.edu.pl (R.M.K.)

[2] Isobolographic Analysis Laboratory, Institute of Rural Health, Jaczewskiego 2, 20-950 Lublin, Poland; zagaja.miroslaw@imw.lublin.pl (M.Z.); andres.marta@imw.lublin.pl (M.A-M.); szewczyk.aleksandra@imw.lublin.pl (A.S.)

[3] Department of Technology and Biotechnology of Drugs, Faculty of Pharmacy, Jagiellonian University Medical College, Medyczna 9, 30-688 Krakow, Poland; gniewomir.latacz@uj.edu.pl

[4] Department of Pharmacodynamics, Centre for Preclinical Research and Technology, Medical University of Warsaw, Banacha 1B, 02-097 Warsaw, Poland; bartlomiej.szulczyk@wum.edu.pl

[5] Department of Animal Physiology and Pharmacology, Institute of Biological Sciences, Faculty of Biology and Biotechnology, Maria Curie-Skłodowska University, Akademicka 19, 20-033 Lublin, Poland; katarzyna.socala@mail.umcs.pl (K.S.); dorota.nieoczym@mail.umcs.pl (D.N.); piotr.wlaz@mail.umcs.pl (P.W.)

[6] Department of Pharmacology and Toxicology, University of Utah, Salt Lake City, UT 84112, USA

* Correspondence: k.kaminski@uj.edu.pl; Tel.: +48-12-620-54-5

Table of contents

Table S1. Anticonvulsant activity screening data for compounds **45–66** and **BCTC** in mice *i.p.*............. 2

Table S2. Radioligand binding and functional assays.. 3

Table S3. *In vitro* functional assays of compounds **45–66** for TRPV1 channel (concentration 100 μM). . 3

Figure S1. The MetaSite 6.0.1. software prediction of the most probably sites of tested compounds metabolism.. 4

Figure S2. UPLC spectra after 120 min incubation of compound **53** with human liver microsomes 5

Figure S3. MS ion fragment analyses and the most probable structure of **53** metabolite **M1**.................. 5

Figure S4. UPLC spectra after 120 min incubation of compound **60** with human liver microsomes 6

Figure S5. MS ion fragment analyses and the most probable structure of **60** metabolites **M1–M2**. 6

Figure S6. UPLC spectra after 120 min incubation of compound **62** with human liver microsomes 7

Figure S7. MS ion fragment analyses and the most probable structure of **62** metabolites **M1–M2**. 7

Figure S8. UPLC spectra after 120 min incubation of the reference drug verapamil with human liver microsomes 8

References:.. 9

^{1}H NMR and ^{13}C NMR spectra for the final compounds (**45–66**).. 10

Supporting Information

Table S1. Anticonvulsant activity screening data for compounds **45–66** and **BCTC** in mice *i.p.*

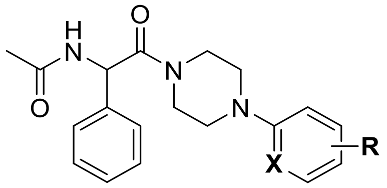

Cmpd	X	R	MES[a]	6 Hz (32 mA)[b]	*sc*PTZ[c]
**45**	**C**	H	3/4	3/4	0/4
**46**	**C**	2-Cl	0/4	2/4	-
**47**	**C**	3-Cl	0/4	3/4	-
**48**	**C**	4-Cl	1/4	3/4	-
**49**	**C**	3,4-diCl	0/4	1/4	-
**50**	**C**	3,5-diCl	0/1	1/4	-
**51**	**C**	3-Cl,5-CF_3	0/4	1/4	-
**52**	**C**	2-CF_3	0/4	2/4	-
**53**	**C**	3-CF_3	3/4	4/4	1/4
**54**	**C**	4-CF_3	0/4	2/4	-
**55**	**C**	3,5-diCF_3	0/4	1/4	-
**56**	**C**	3-CH_3	0/4	1/4	-
**57**	**C**	3-CHF_2	0/4	1/4	-
**58**	**C**	3-C_6H_5	0/4	2/4	-
**59**	**C**	3-OCH_3	0/4	-	-
**60**	**C**	3-OCF_3	3/4	4/4	1/4
**61**	**C**	3-OC_6H_5	0/4	1/4	-
**62**	**C**	3-SCF_3	3/4	4/4	0/4
**63**	**N**	3-CF_3	0/4	-	-
**64**	**N**	4-CF_3	0/4	1/4	-
**65**	**N**	5-CF_3	3/4	3/4	0/4
**66**	**N**	6-CF_3	0/4	2/4	-
**BCTC**[d]	**-**	-	0/4	0/4	0/4

Data indicate number of mice protected/number of mice tested. Dose of 100 mg/kg was administered *i.p.* The animals were examined at 0.5 h. The most potent compounds are marked in blue. A dash indicates not tested. [a] MES – maximal electroshock seizure test; [b] 6 Hz – psychomotor seizure test, 32 mA; [c] *sc*PTZ – subcutaneous pentylenetetrazole seizure test. [d] BCTC – model TRPV1 antagonist

Supporting Information

Table S2. Radioligand binding and functional assays.

**Binding studies**	
Na^+ channel (site 2)	[1]
Calcium $Ca_{v1.2}$ channels (dihydropyridine site, antagonist radioligand)	[2]
NMDA (antagonist radioligand)	[3]
N-type Ca^{2+} (antagonist radioligand)	[4]
GABA transporter (antagonist radioligand)	[5]
$GABA_A$ ion channel [^{3}H]GABA (agonist radioligand)	[6]
Potassium channel (hERG)	[7]
**Functional studies**	
TRPV1 (VR1) (h) (antagonist effect)	[8]
$Ca_{v1.2}$ (L-type) (h) calcium ion channel cell-based antagonist calcium flux assay	[9,10]

Assays were performed commercially in Eurofins Laboratories (Poitiers, France) or Eurofins Panlabs Discovery Services Taiwan, Ltd. (New Taipei City, Taiwan).

Table S3. *In vitro* TRPV1 channel antagonist activity for compounds **45–66** (concentration of 100 μM).

TRPV1 (VR1) (*h*) (antagonist effect)*			
Cmpd	% Inhibition of control agonist response[a]	Cmpd	% Inhibition of control agonist response[a]
**45**	36.1	**56**	57.4
**46**	116.3	**57**	43.7
**47**	125.2	**58**	92.0
**48**	126.9	**59**	20.1
**49**	135.1	**60**	109.7
**50**	128.0	**61**	52.5
**51**	-2.5	**62**	135.1
**52**	90.9	**63**	-2.8
**53**	128.5	**64**	39.7
**54**	128.5	**65**	8.5
**55**	-14.0	**66**	3.7

*Source: human recombinant CHO cells. [a] **Results showing activity higher than 50% are considered to represent significant effects of the test compounds**; results showing an inhibition between 25% and 50% are indicative of weak effect; results showing an inhibition lower than 25% are not considered significant and mostly attributable to variability of the sign al around the control level. Assays were performed commercially in Eurofins Laboratories (Poitiers, France).

Supporting Information

ADME-Tox in vitro - metabolic stability of compounds **53**, **60**, and **62**.

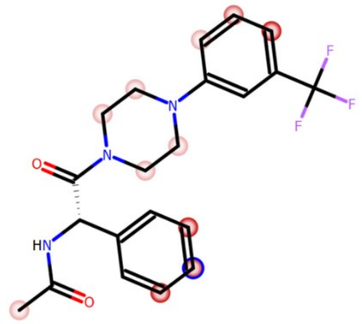

53

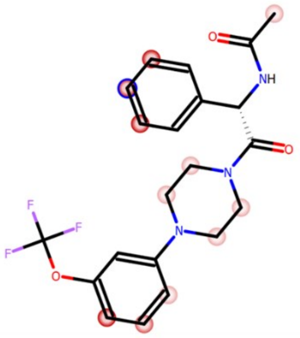

60

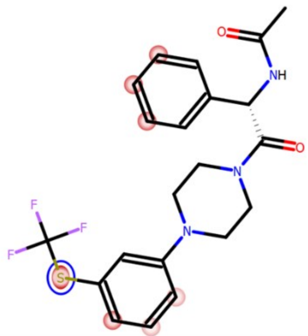

62

Figure S1. The MetaSite 6.0.1. software prediction of the most probably sites of tested compounds metabolism. The darker red color - the higher probability to be involved in the metabolism pathway. The blue circle marked the site of compound with the highest probability of metabolic bioconversion.

Supporting Information

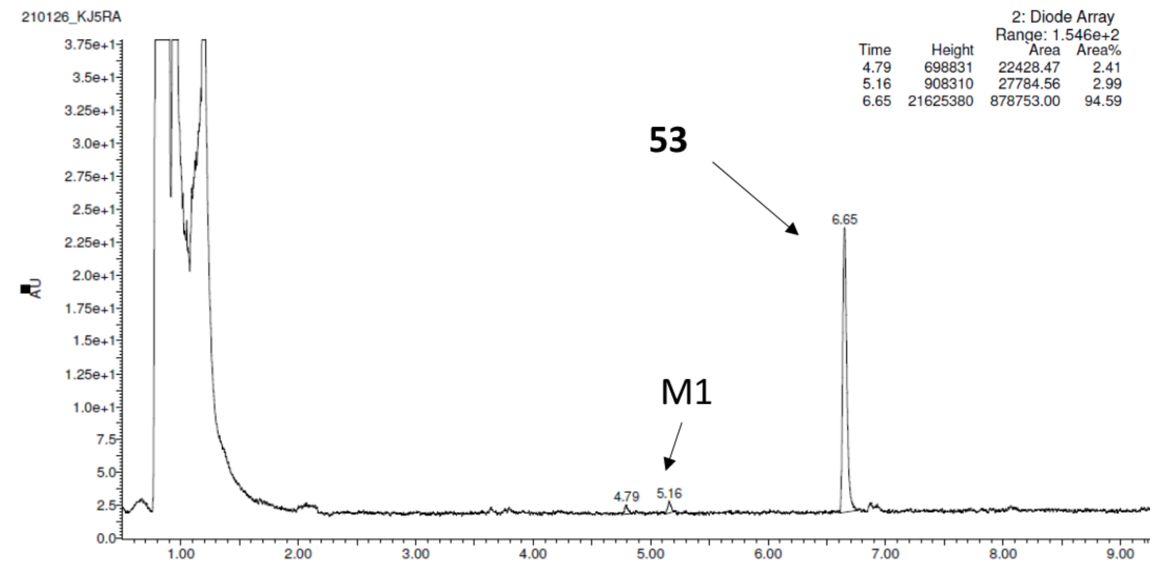

Figure S2. UPLC spectra after 120 min incubation of compound **53** with human liver microsomes **i**n TRIS buffer pH=7.4 at 37ºC.

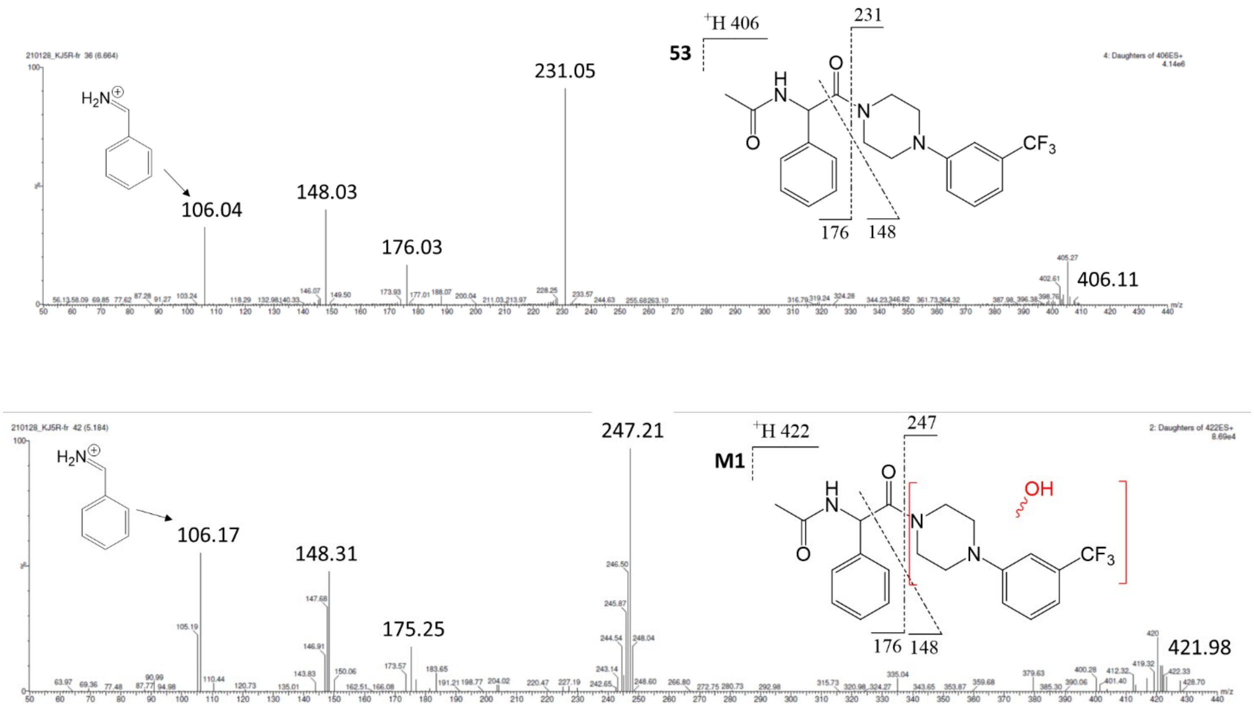

Figure S3. MS ion fragment analyses and the most probable structure of **53** metabolite **M1**.

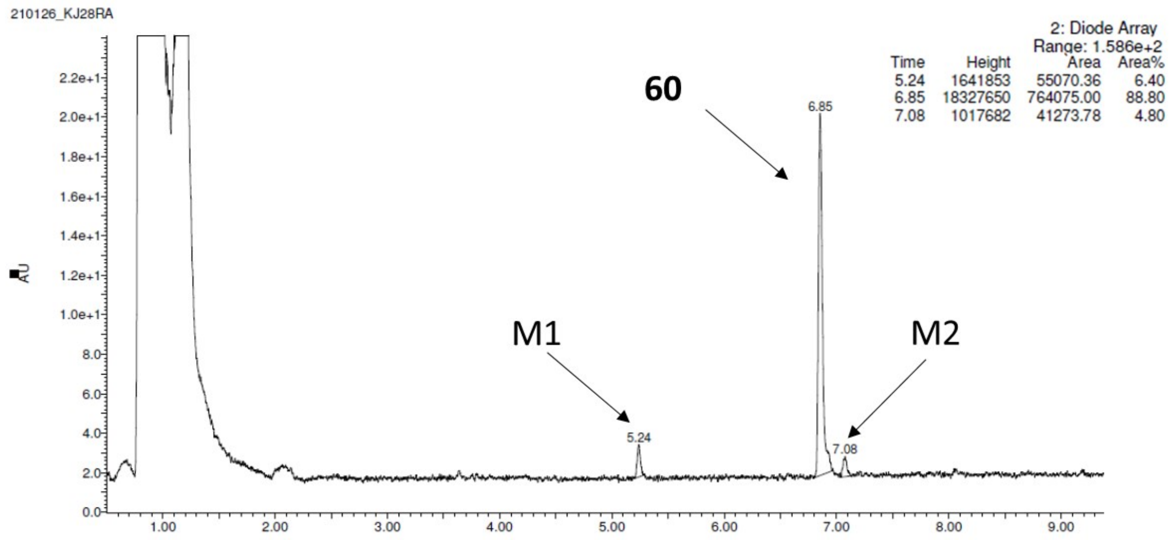

Figure S4. UPLC spectra after 120 min incubation of compound **60** with human liver microsomes in TRIS buffer pH=7.4 at 37ºC.

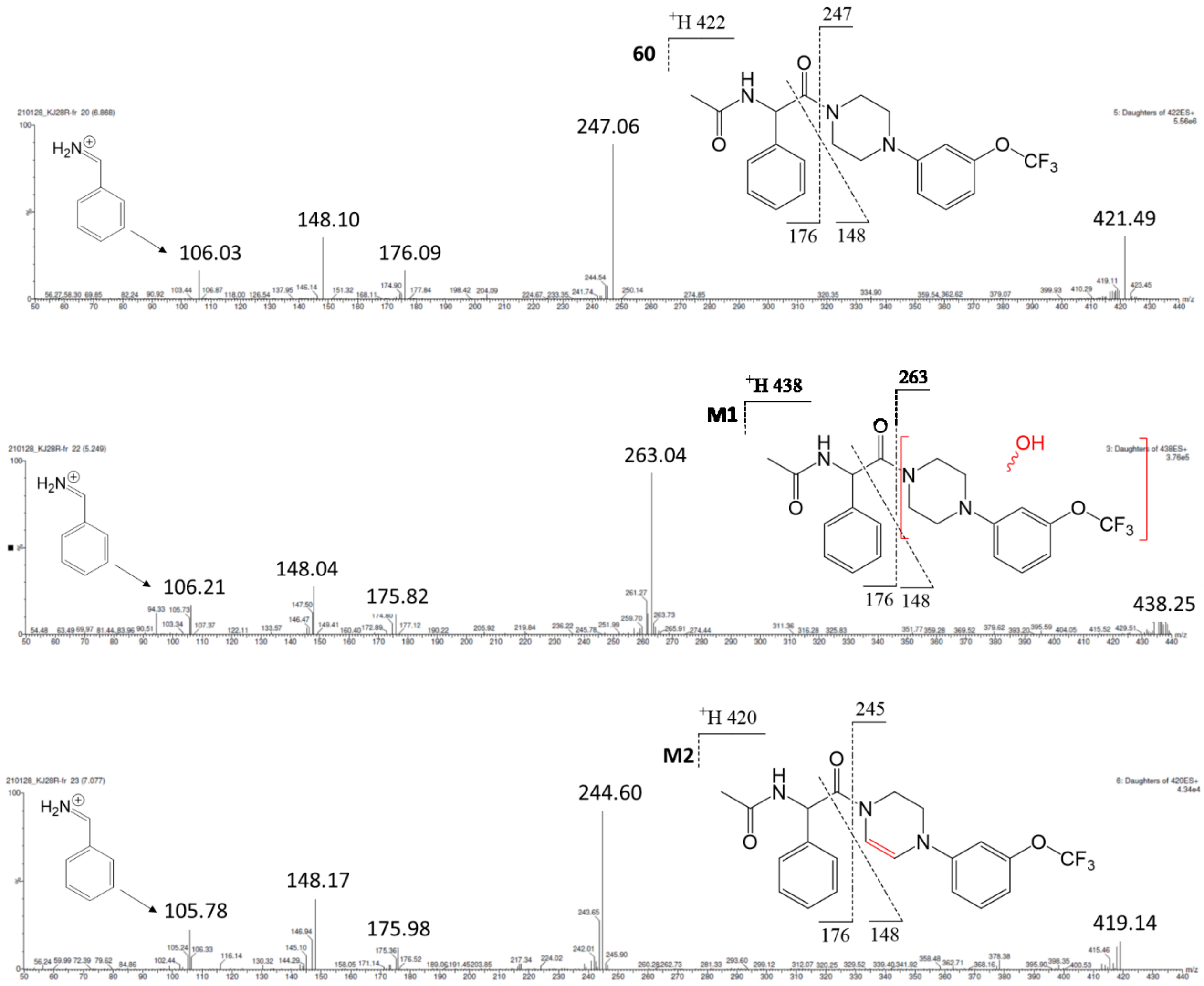

Figure S5. MS ion fragment analyses and the most probable structure of **60** metabolites **M1**–**M2**.

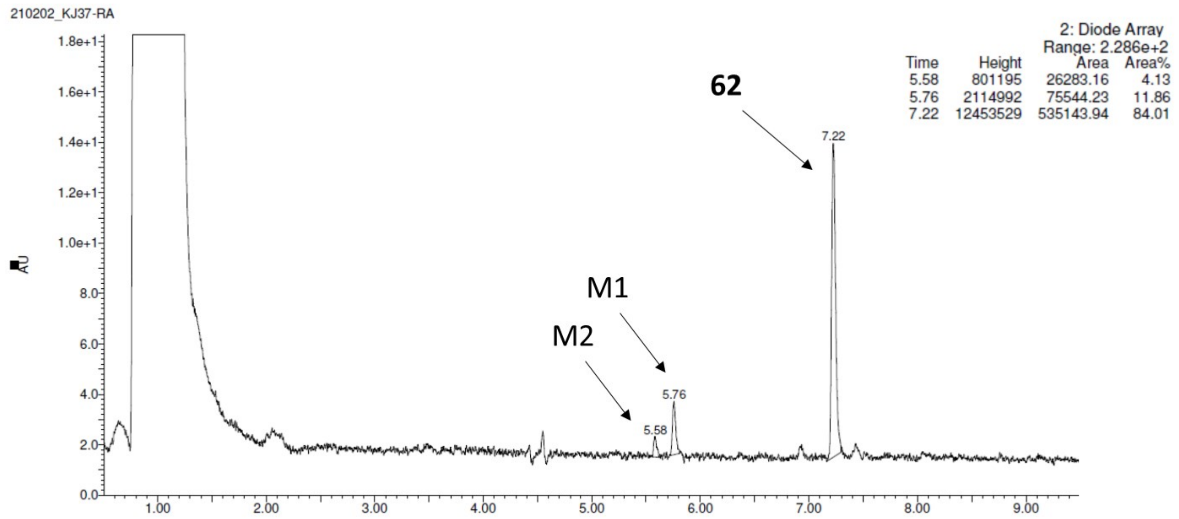

Figure S6. UPLC spectra after 120 min incubation of compound **62** with human liver microsomes in TRIS buffer pH=7.4 at 37ºC.

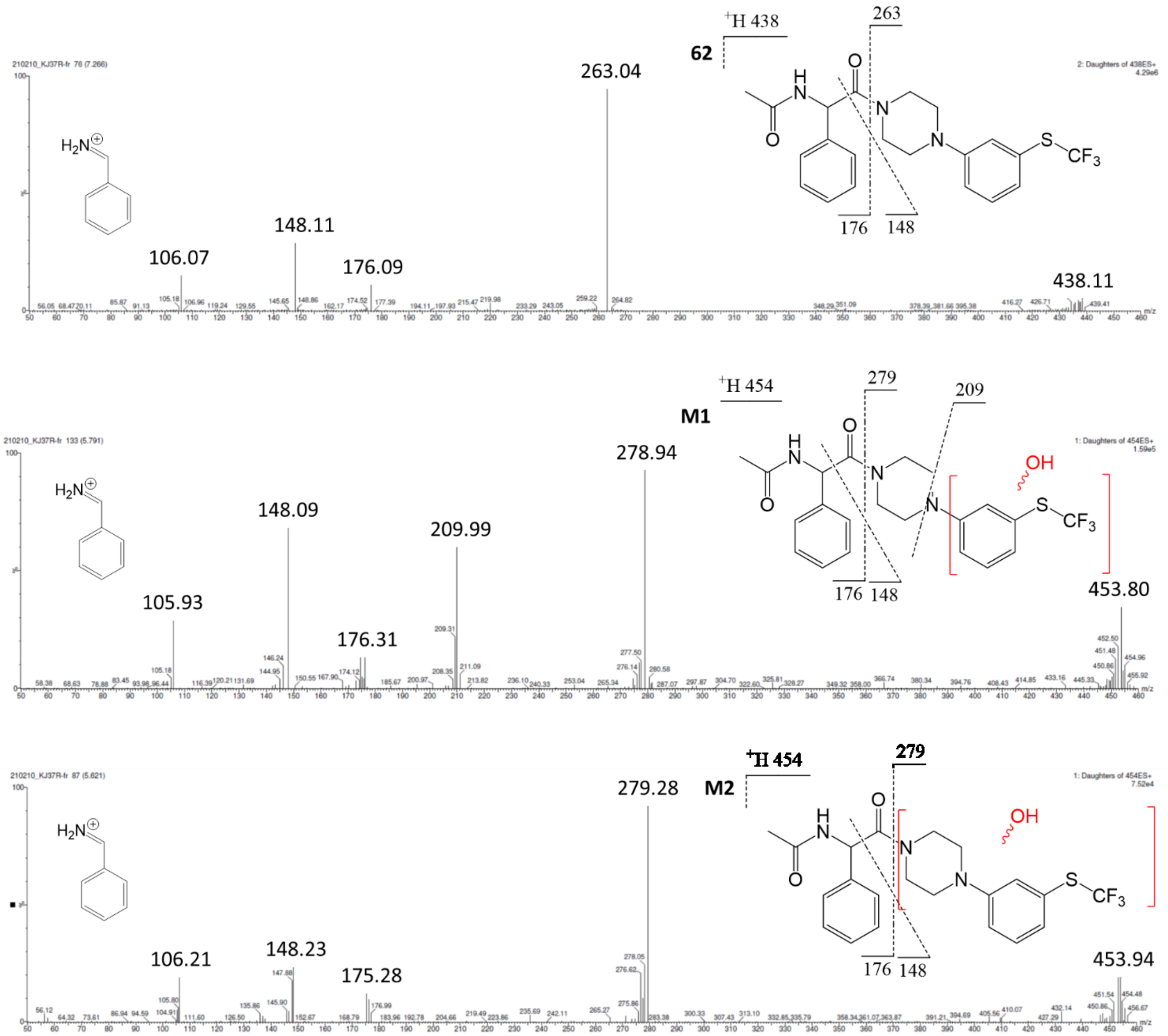

Figure S7. MS ion fragment analyses and the most probable structure of **62** metabolites **M1**–**M2**.

Supporting Information

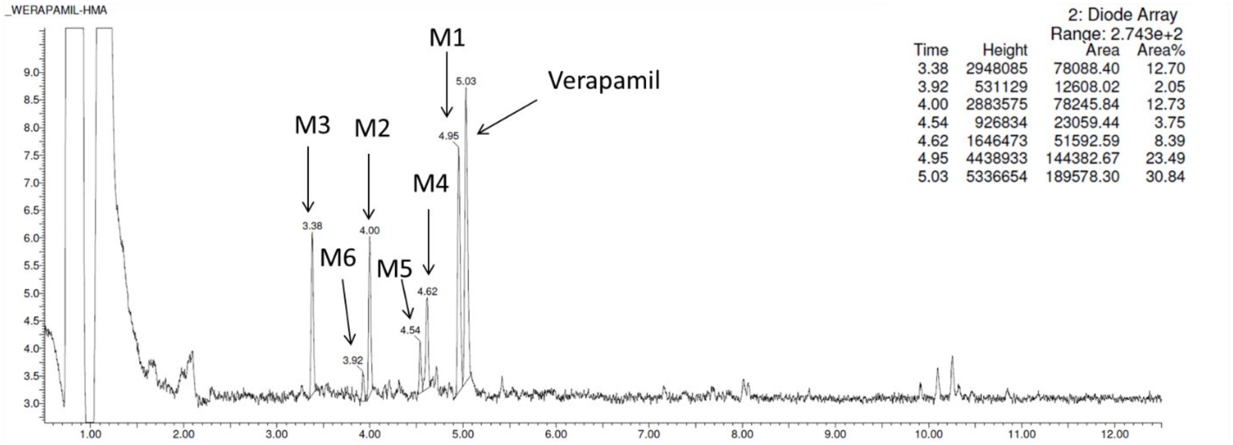

Figure S8. UPLC spectra after 120 min incubation of the reference drug verapamil with human liver microsomes in TRIS buffer pH=7.4 at 37ºC [11–13]

Supporting Information

References:

1. Brown, G.B. 3H-Batrachotoxinin-A Benzoate Binding to Voltage-Sensitive Sodium Channels: Inhibition by the Channel Blockers Tetrodotoxin and Saxitoxin. *J. Neurosci.* **1986**, *6*, 2064–2070, doi:10.1523/JNEUROSCI.06-07-02064.1986.

2. Gould, R.J.; Murphy, K.M.; Snyder, S.H. [3H]Nitrendipine-Labeled Calcium Channels Discriminate Inorganic Calcium Agonists and Antagonists. *Proc. Natl. Acad. Sci. U.S.A.* **1982**, *79*, 3656–3660, doi:10.1073/pnas.79.11.3656.

3. Sills, M.A.; Fagg, G.; Pozza, M.; Angst, C.; Brundish, D.E.; Hurt, S.D.; Jay Wilusz, E.; Williams, M. [3H]CGP 39653: A New N-Methyl-D-Aspartate Antagonist Radioligand with Low Nanomolar Affinity in Rat Brain. *Eur. J. Pharmacol.* **1991**, *192*, 19–24, doi:10.1016/0014-2999(91)90063-V.

4. Wagner, J.A.; Snowman, A.M.; Biswas, A.; Olivera, B.M.; Snyder, S.H. Omega-Conotoxin GVIA Binding to a High-Affinity Receptor in Brain: Characterization, Calcium Sensitivity, and Solubilization. *J. Neurosci.* **1988**, *8*, 3354–3359, doi:10.1523/JNEUROSCI.08-09-03354.1988.

5. Shank, R.P.; Baldy, W.J.; Mattucci, L.C.; Villani Jr., F.J. Ion and Temperature Effects on the Binding of γ-Aminobutyrate to Its Receptors and the High-Affinity Transport System. *J. Neurochem.* **1990**, *54*, 2007–2015, doi:10.1111/j.1471-4159.1990.tb04905.x.

6.Eurofins Discovery Https://Www.Eurofinsdiscoveryservices.Com/Catalogmanagement/ViewItem/Non-Selective-Rat GABAA-Ion-Channel-3H-Muscimol-Binding-Agonist-Radioligand-Assay-Panlabs/226500 (Accessed 2021 -12 -15).

7. Huang, X.-P.; Mangano, T.; Hufeisen, S.; Setola, V.; Roth, B.L. Identification of Human Ether-à-Go-Go Related Gene Modulators by Three Screening Platforms in an Academic Drug-Discovery Setting. *ASSAY and Drug Dev. Technol.* **2010**, *8*, 727–742, doi:10.1089/adt.2010.0331.

8. Phelps, P.T.; Anthes, J.C.; Correll, C.C. Cloning and Functional Characterization of Dog Transient Receptor Potential Vanilloid Receptor-1 (TRPV1). *Eur. J. Pharmacol.* **2005**, *513*, 57–66, doi:10.1016/j.ejphar.2005.02.045.

9. Sirenko, O.; Crittenden, C.; Callamaras, N.; Hesley, J.; Chen, Y.-W.; Funes, C.; Rusyn, I.; Anson, B.; Cromwell, E.F. Multiparameter In Vitro Assessment of Compound Effects on Cardiomyocyte Physiology Using IPSC Cells. *J. Biomol. Screen.* **2013**, *18*, 39–53, doi:10.1177/1087057112457590.

10. Xia, M.; Imredy, J.P.; Koblan, K.S.; Bennett, P.; Connolly, T.M. State-Dependent Inhibition of L-Type Calcium Channels: Cell-Based Assay in High-Throughput Format. *Anal. Biochem.* **2004**, *327*, 74–81, doi:10.1016/j.ab.2004.01.003.

11. Obach, R.S. Prediction of Human Clearance of Twenty-Nine Drugs from Hepatic Microsomal Intrinsic Clearance Data: An Examination of In Vitro Half-Life Approach and Nonspecific Binding to Microsomes. *Drug. Metab. Dispos.* **1999**, *27*, 1350–1359.

12. Pauli-Magnus, C.; Richter, O. von; Burk, O.; Ziegler, A.; Mettang, T.; Eichelbaum, M.; Fromm, M.F. Characterization of the Major Metabolites of Verapamil as Substrates and Inhibitors of P-Glycoprotein. *J. Pharmacol. Exp. Ther.* **2000**, *293*, 376–382.

13. Więckowska, A.; Wichur, T.; Godyń, J.; Bucki, A.; Marcinkowska, M.; Siwek, A.; Więckowski, K.; Zaręba, P.; Knez, D.; Głuch-Lutwin, M.; et al. Novel Multitarget-Directed Ligands Aiming at Symptoms and Causes of Alzheimer's Disease. *ACS Chem. Neurosci.* **2018**, *9*, 1195–1214, doi:10.1021/acschemneuro.8b00024.

^{1}H NMR and ^{13}C NMR spectra for the final compounds (45–66)

N-(2-oxo-1-phenyl-2-(4-phenylpiperazin-1-yl)ethyl)acetamide (45) – ^{1}H NMR

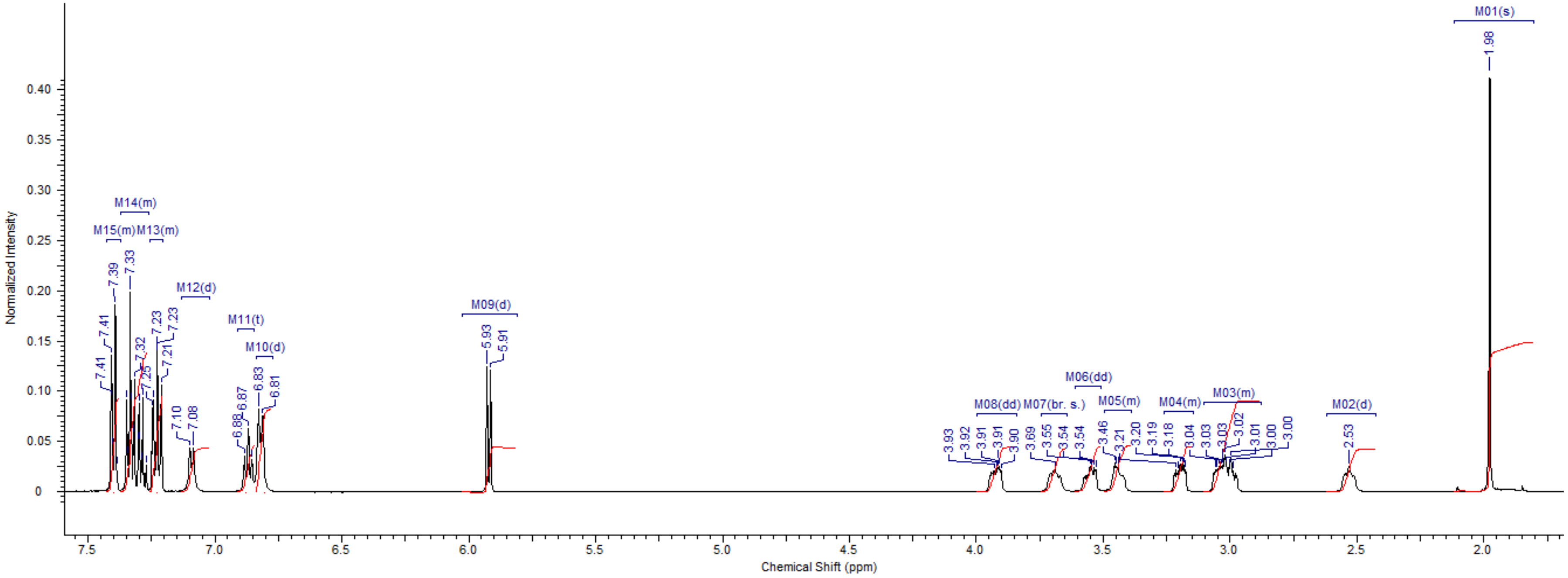

***N*-(2-oxo-1-phenyl-2-(4-phenylpiperazin-1-yl)ethyl)acetamide (45)** – ^{13}C NMR

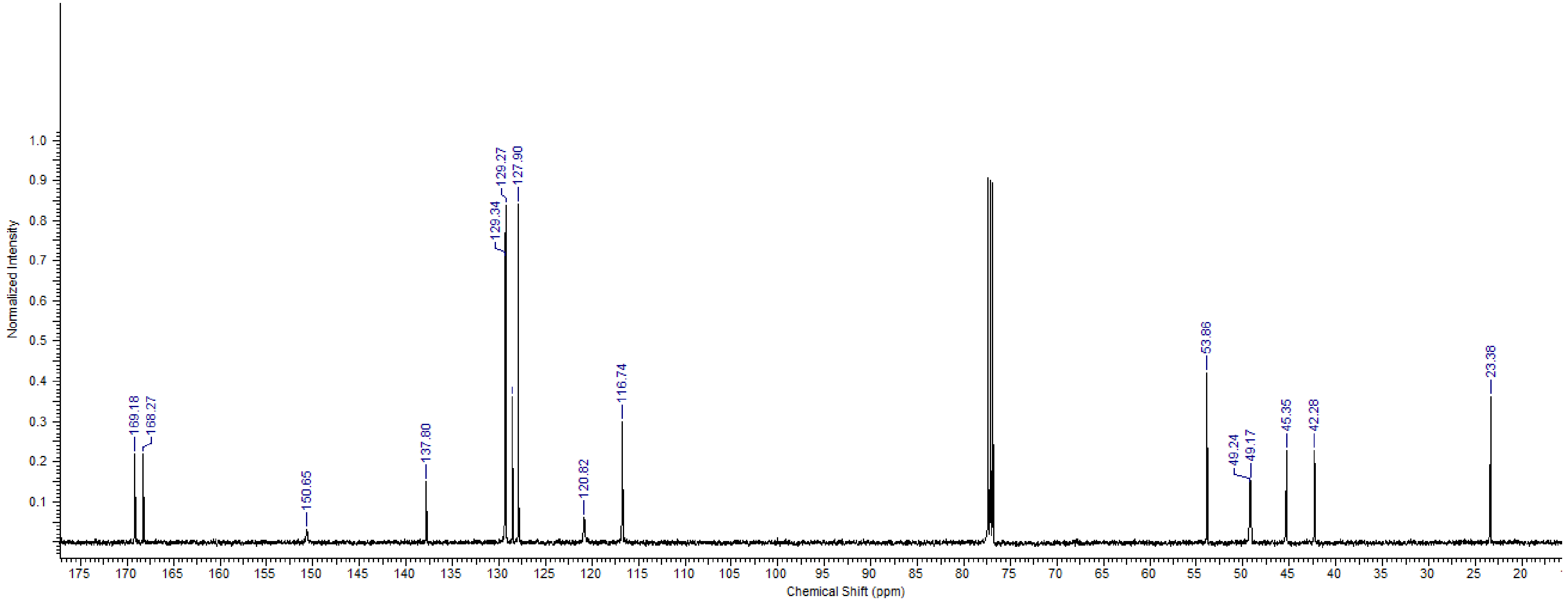

Supporting Information

N-(2-(4-(2-chlorophenyl)piperazin-1-yl)-2-oxo-1-phenylethyl)acetamide (46) – ^{1}H NMR

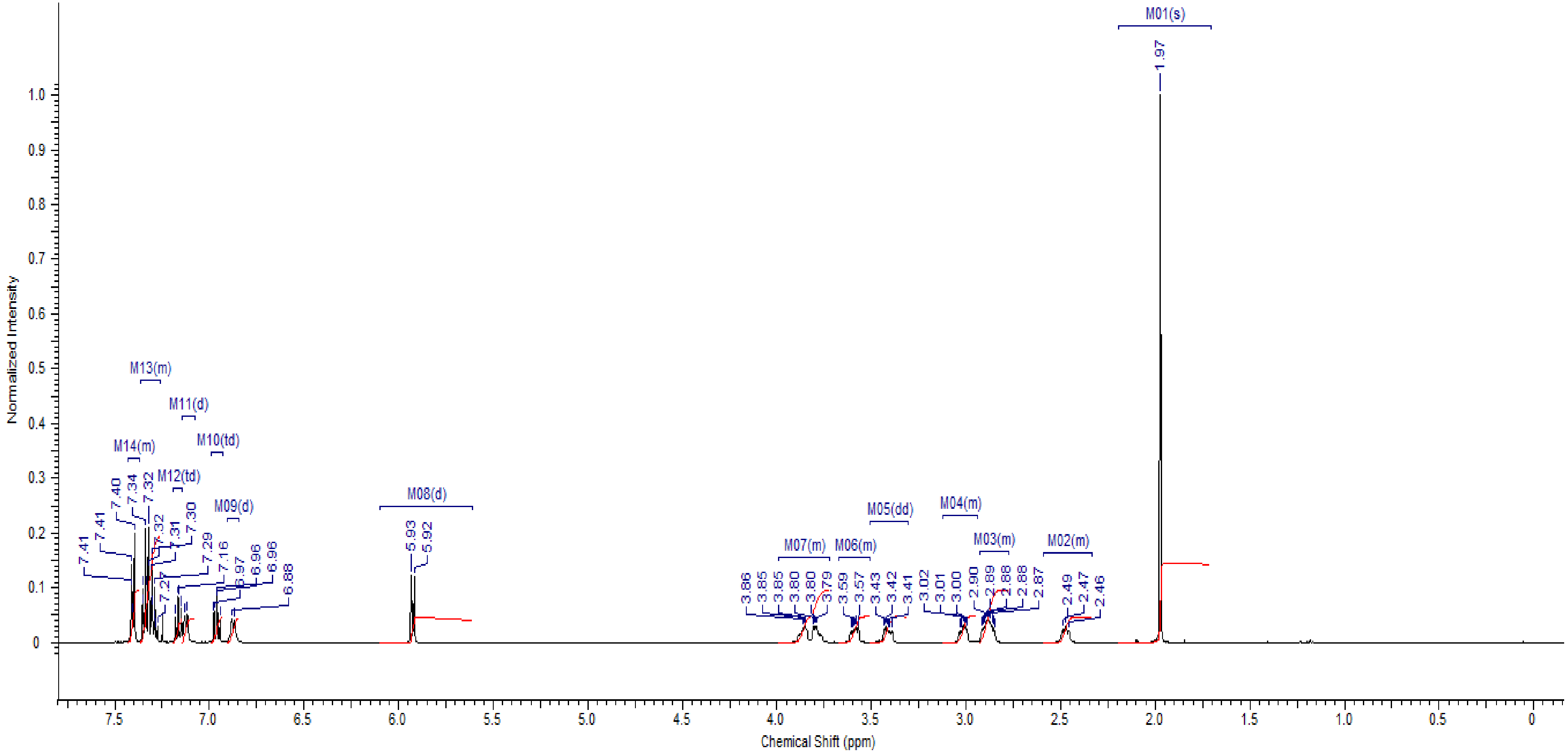

***N*-(2-(4-(2-chlorophenyl)piperazin-1-yl)-2-oxo-1-phenylethyl)acetamide (46) –** ^{13}C NMR

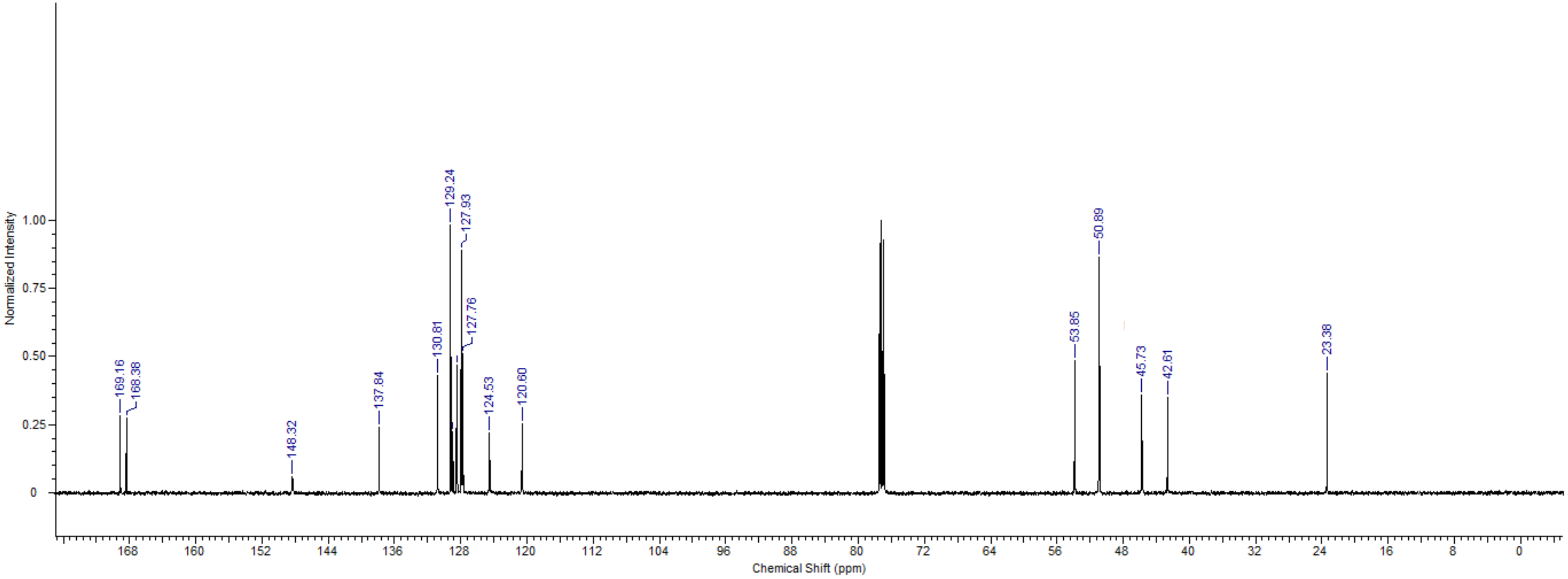

Supporting Information

***N*-(2-(4-(3-chlorophenyl)piperazin-1-yl)-2-oxo-1-phenylethyl)acetamide (47)** – ^{1}H NMR

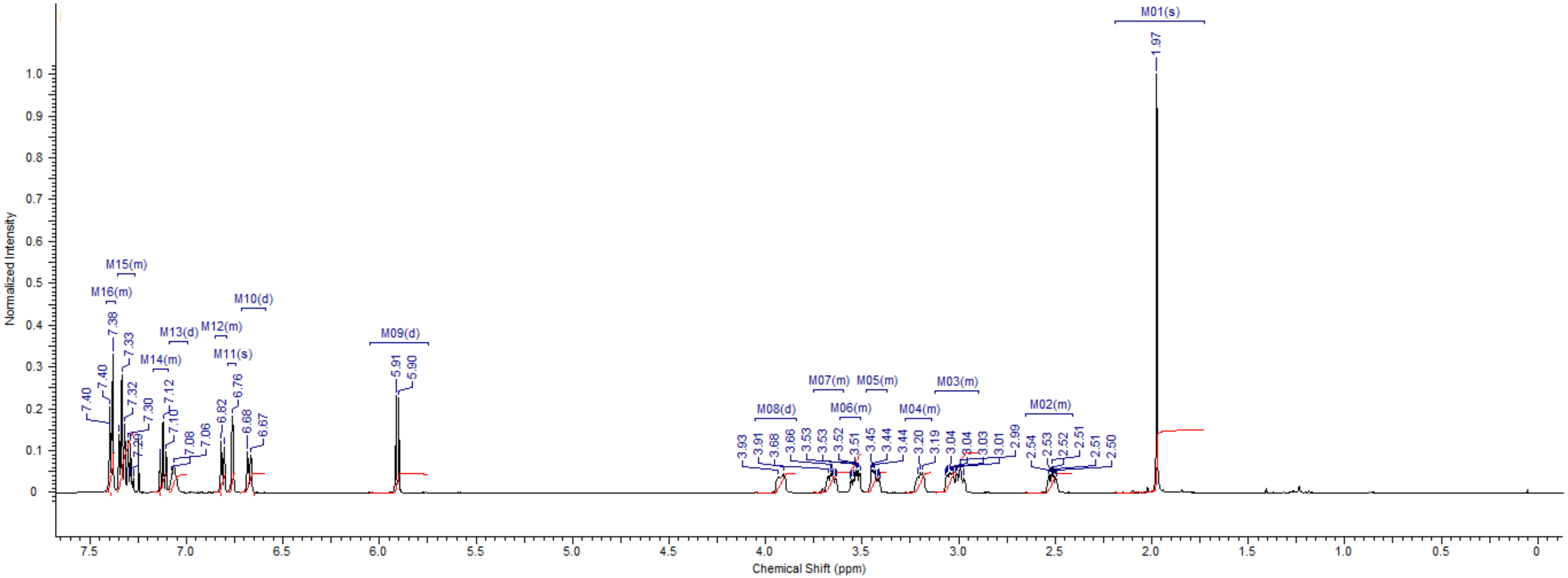

Supporting Information

N-(2-(4-(3-chlorophenyl)piperazin-1-yl)-2-oxo-1-phenylethyl)acetamide (47) – ^{13}C NMR

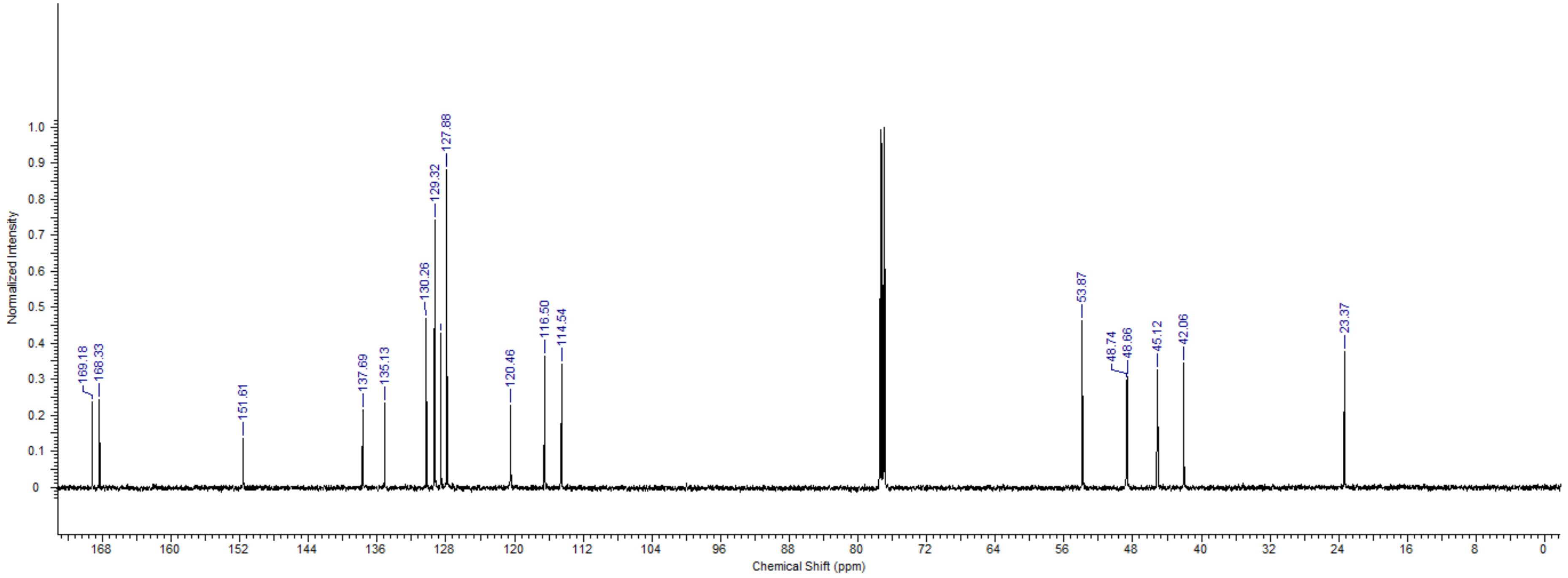

Supporting Information

N-(2-(4-(4-chlorophenyl)piperazin-1-yl)-2-oxo-1-phenylethyl)acetamide (48) – ^{1}H NMR

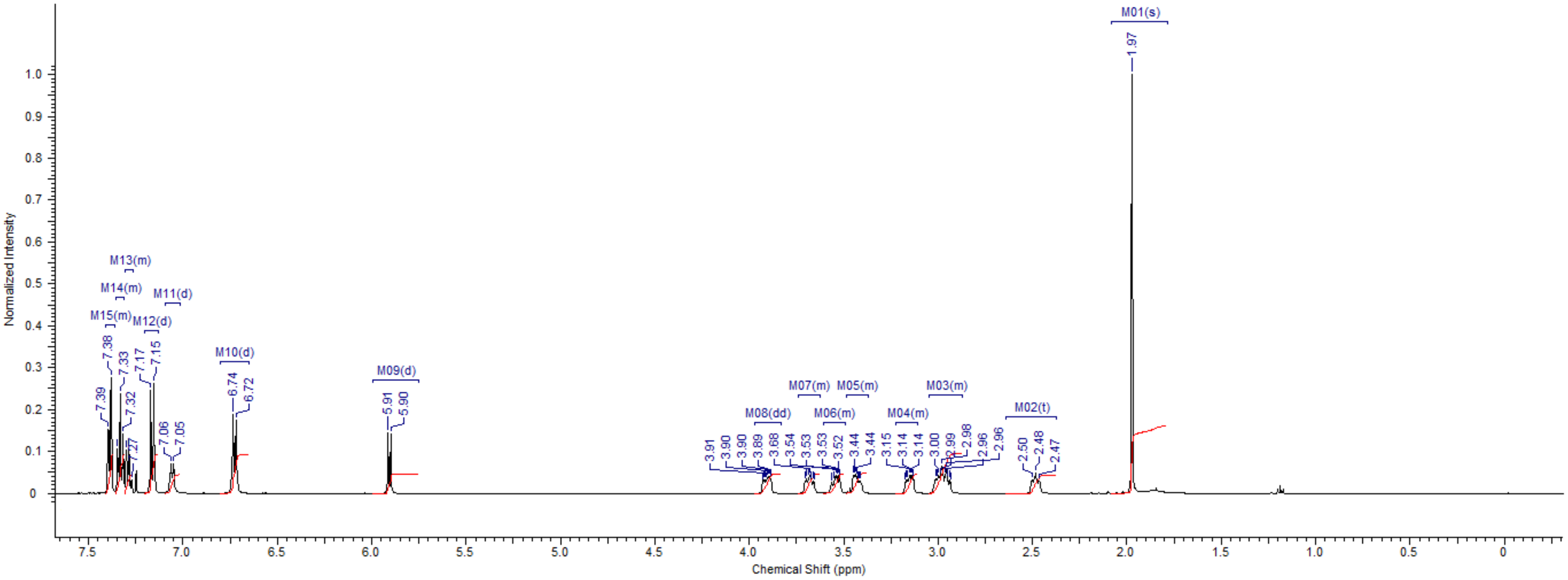

***N*-(2-(4-(4-chlorophenyl)piperazin-1-yl)-2-oxo-1-phenylethyl)acetamide (48)** – ^{13}C NMR

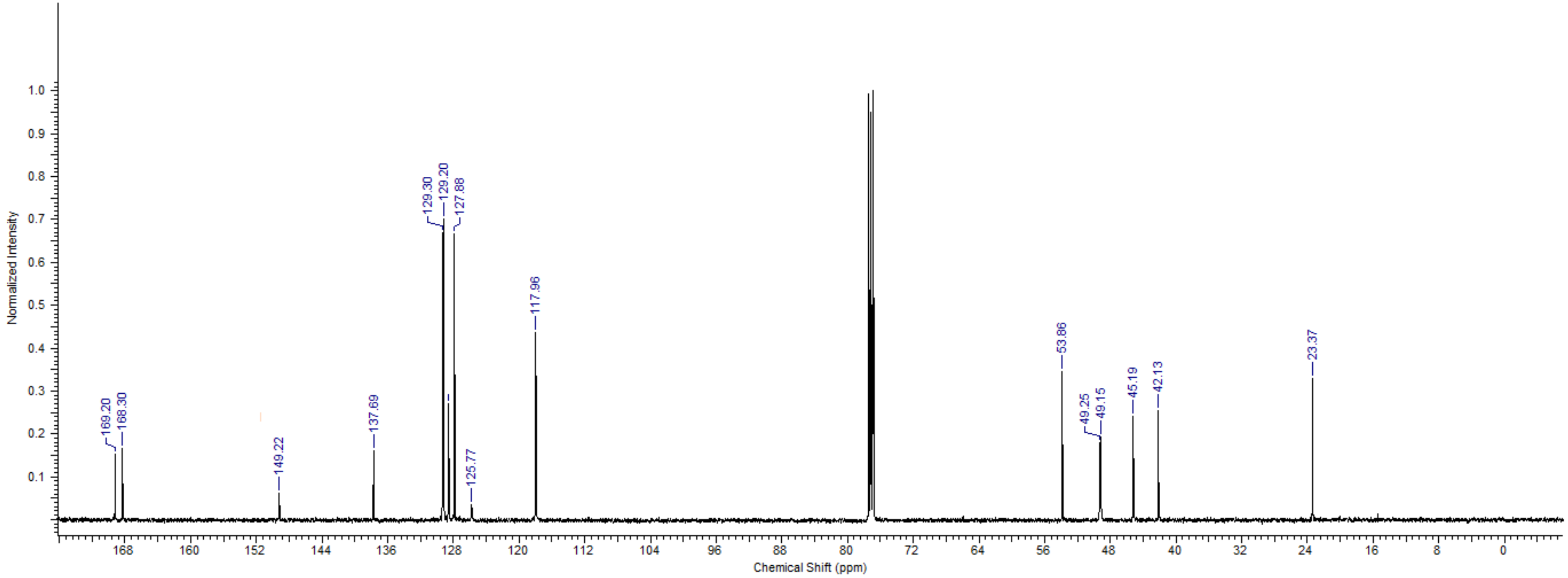

N-(2-(4-(3,4-dichlorophenyl)piperazin-1-yl)-2-oxo-1-phenylethyl)acetamide (49) – ^{1}H NMR

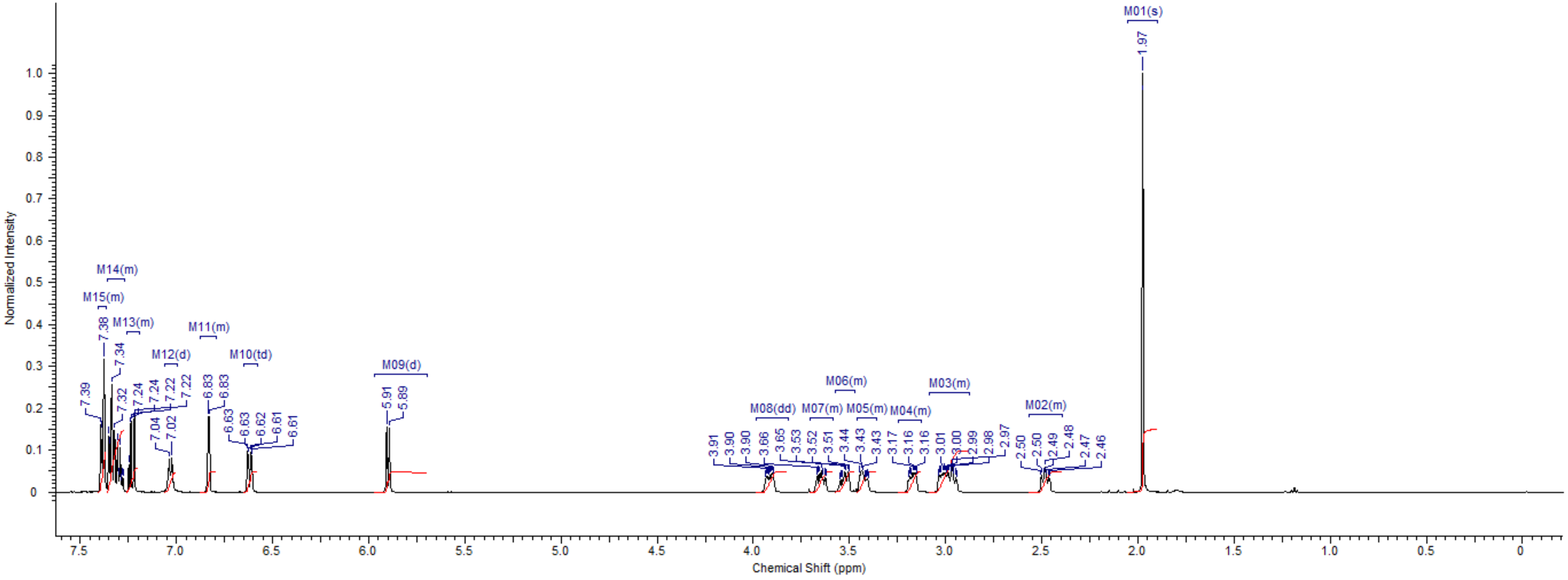

***N*-(2-(4-(3,4-dichlorophenyl)piperazin-1-yl)-2-oxo-1-phenylethyl)acetamide (49)** – ^{13}C NMR

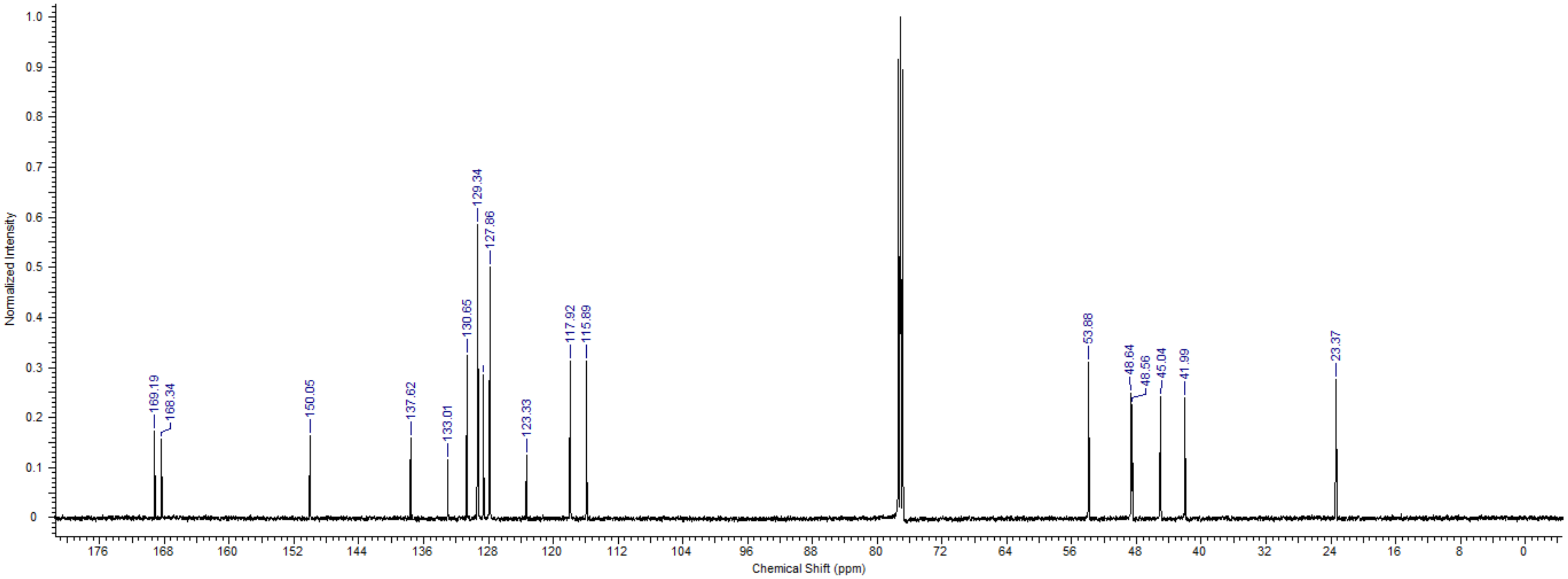

Supporting Information

N-(2-(4-(3,5-dichlorophenyl)piperazin-1-yl)-2-oxo-1-phenylethyl)acetamide (50) – ^{1}H NMR

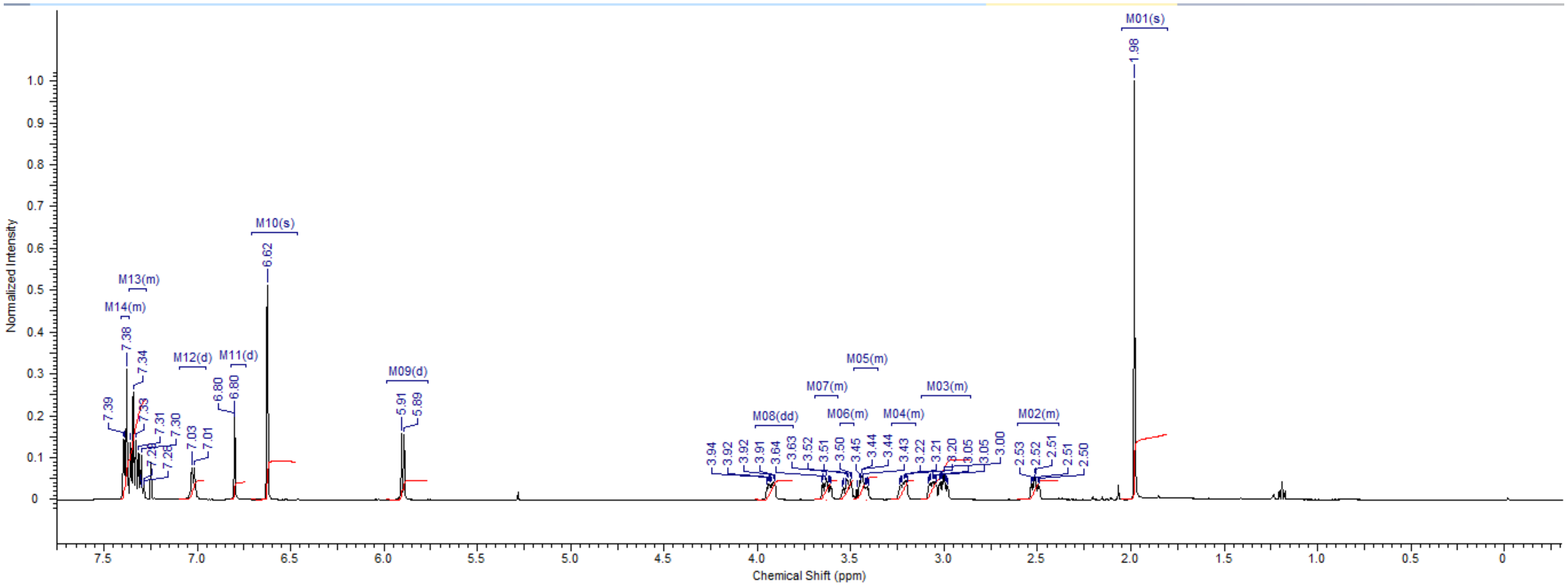

***N*-(2-(4-(3,5-dichlorophenyl)piperazin-1-yl)-2-oxo-1-phenylethyl)acetamide (50) –** ^{13}C NMR

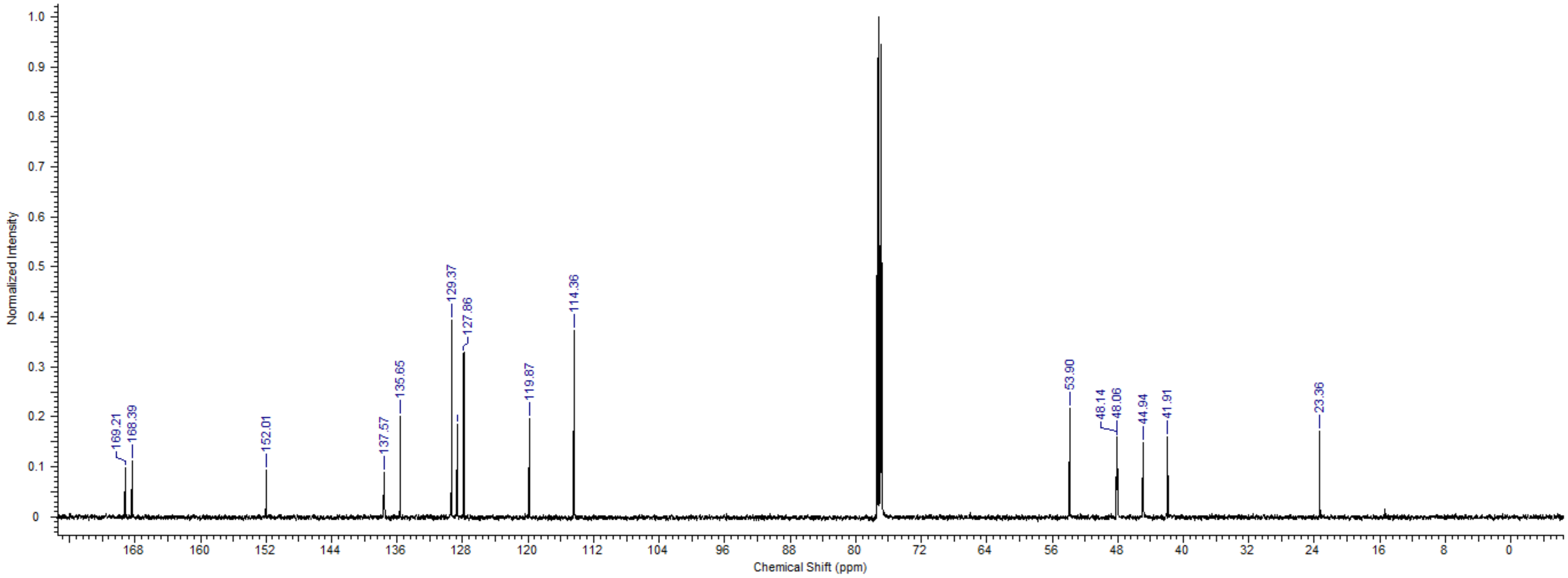

***N*-(2-(4-(3-chloro-5-(trifluoromethyl)phenyl)piperazin-1-yl)-2-oxo-1-phenylethyl) acetamide (51)** – ^{1}H NMR

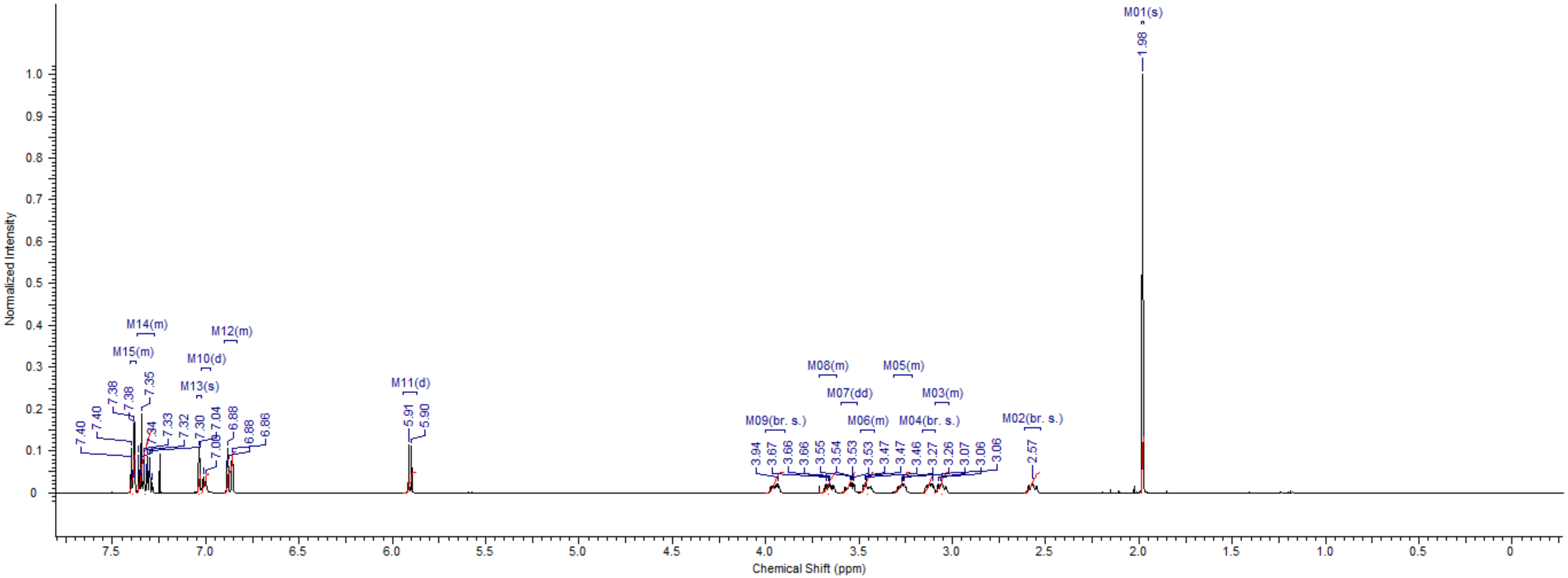

***N*-(2-(4-(3-chloro-5-(trifluoromethyl)phenyl)piperazin-1-yl)-2-oxo-1-phenylethyl) acetamide (51) –** ^{13}C NMR

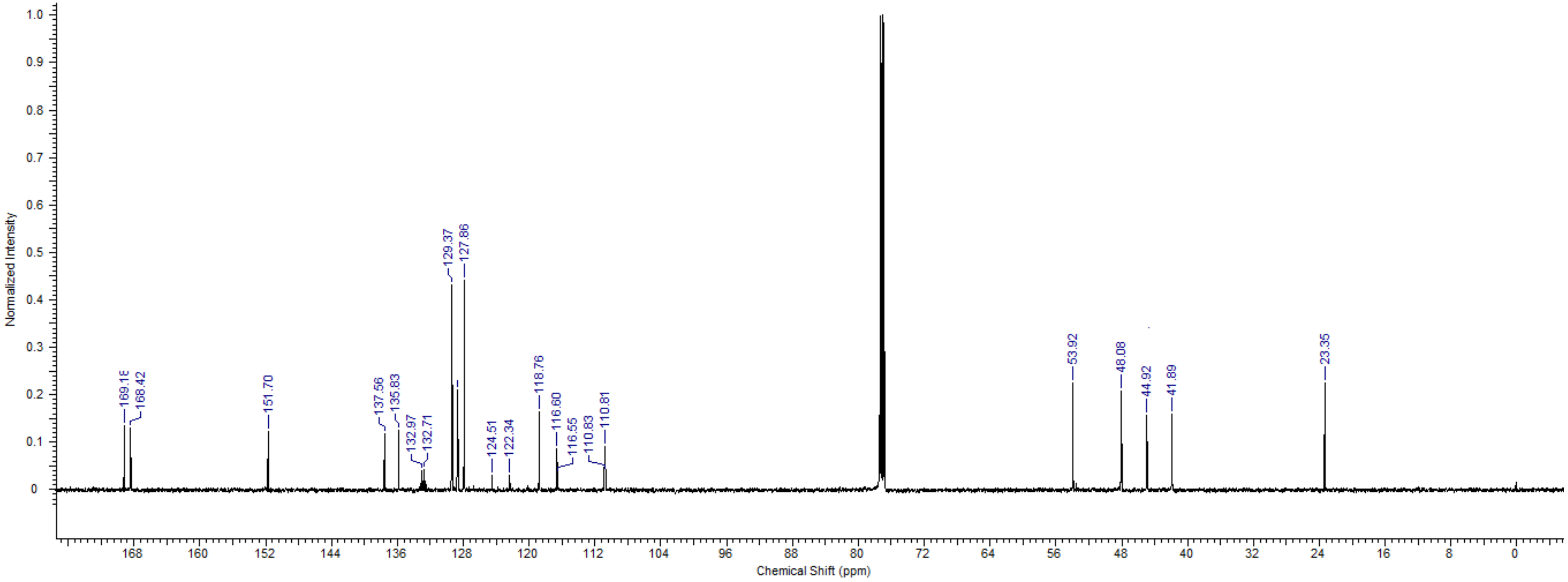

***N*-(2-oxo-1-phenyl-2-(4-(2-(trifluoromethyl)phenyl)piperazin-1-yl)ethyl)acetamide (52)** – ^{1}H NMR

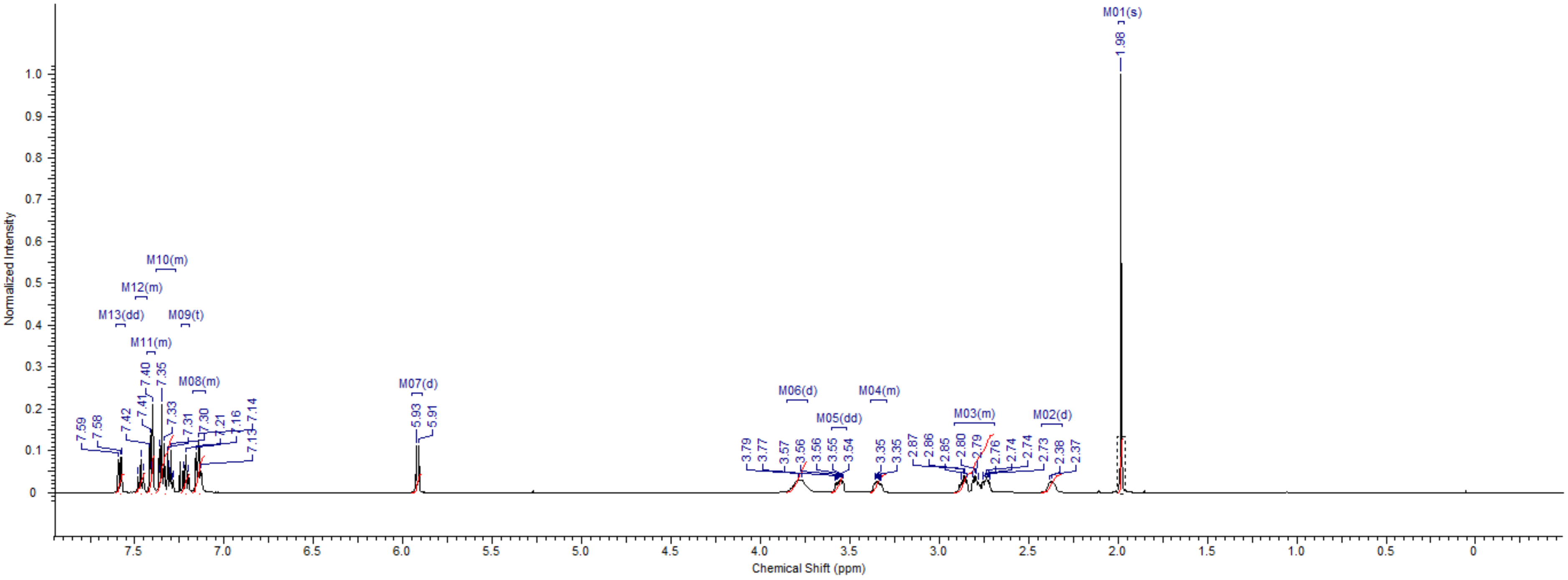

N-(2-oxo-1-phenyl-2-(4-(2-(trifluoromethyl)phenyl)piperazin-1-yl)ethyl)acetamide (52) – ^{13}C NMR

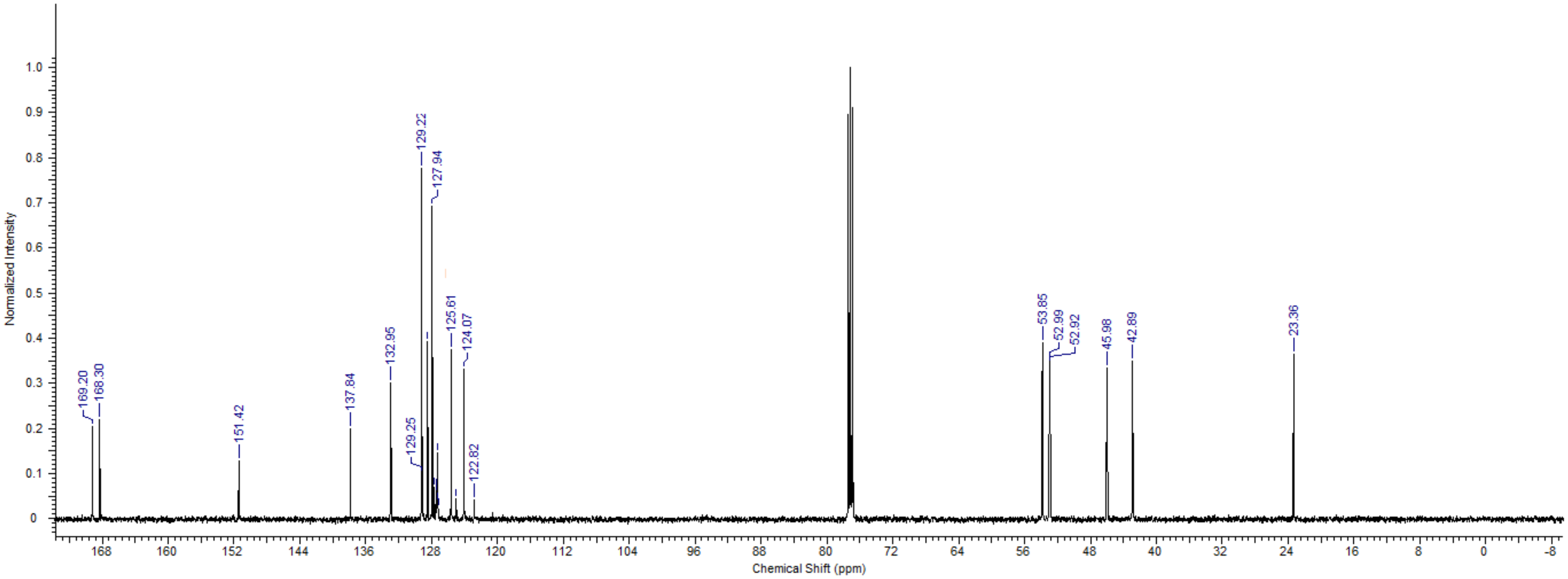

***N*-(2-oxo-1-phenyl-2-(4-(3-(trifluoromethyl)phenyl)piperazin-1-yl)ethyl)acetamide (53)** – ^{1}H NMR

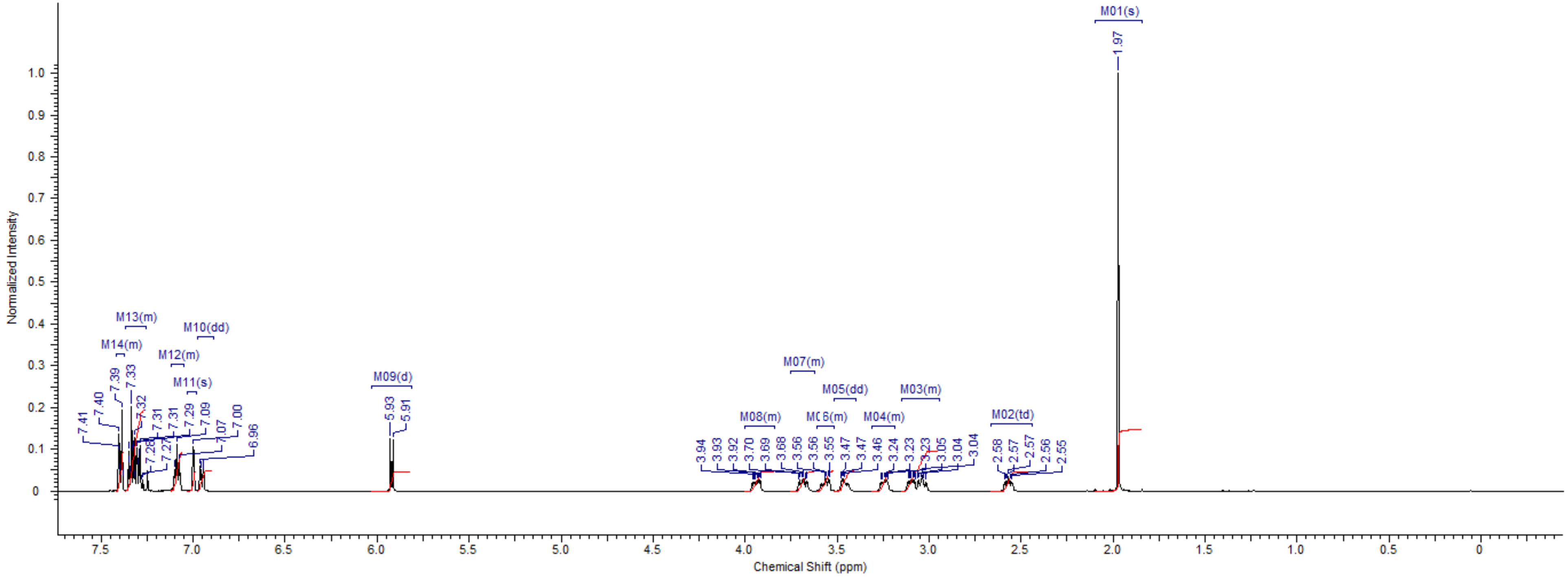

Supporting Information

***N*-(2-oxo-1-phenyl-2-(4-(3-(trifluoromethyl)phenyl)piperazin-1-yl)ethyl)acetamide (53) –** ^{13}C NMR

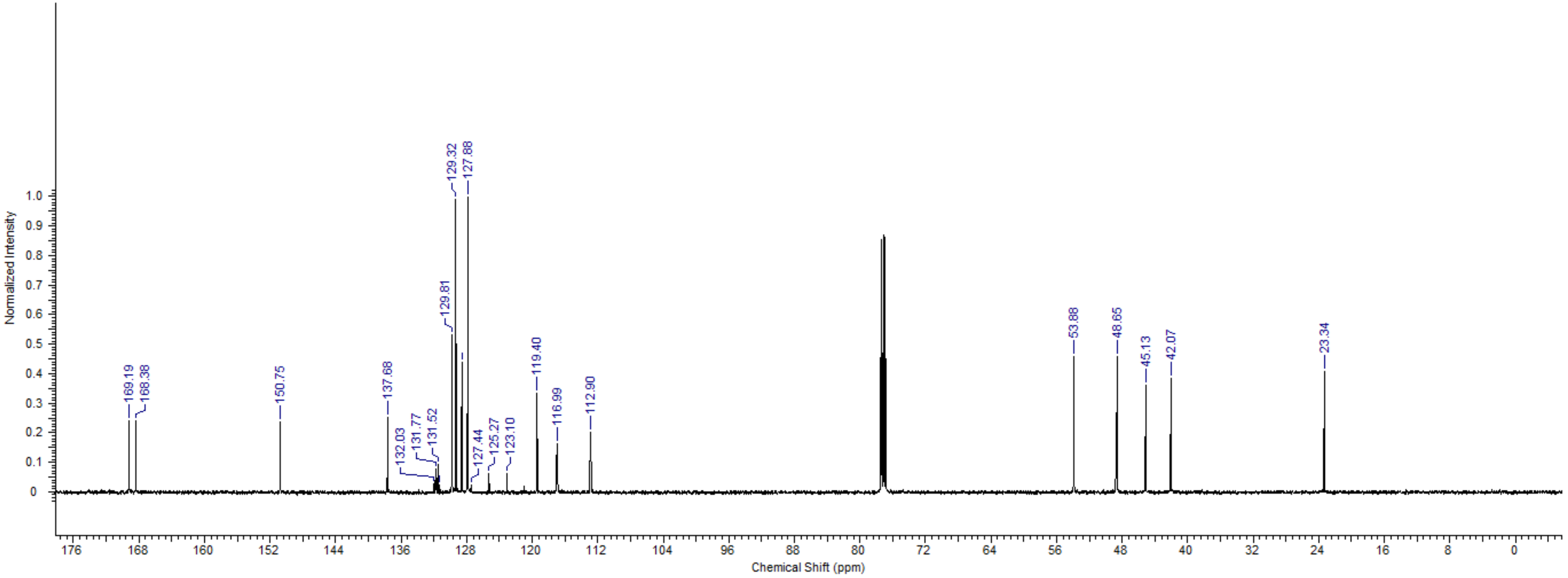

Supporting Information

N-(2-oxo-1-phenyl-2-(4-(4-(trifluoromethyl)phenyl)piperazin-1-yl)ethyl)acetamide (54) – ^{1}H NMR

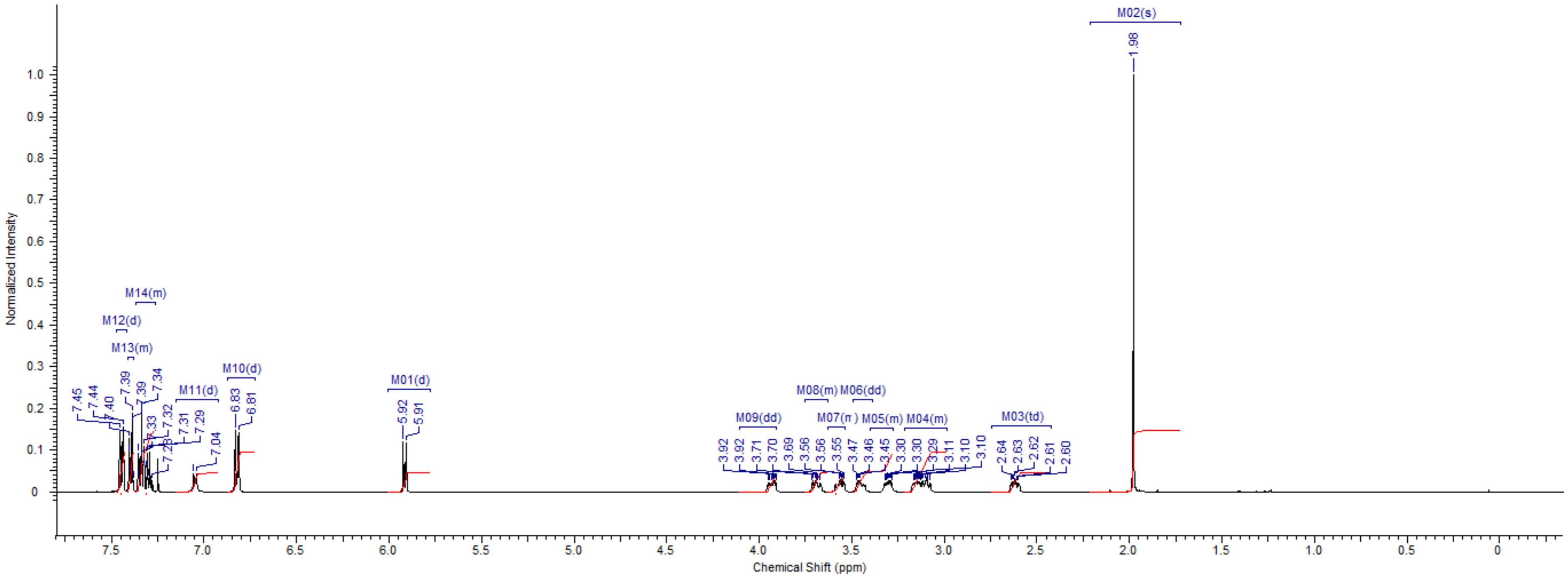

***N*-(2-oxo-1-phenyl-2-(4-(4-(trifluoromethyl)phenyl)piperazin-1-yl)ethyl)acetamide (54) –** ^{13}C NMR

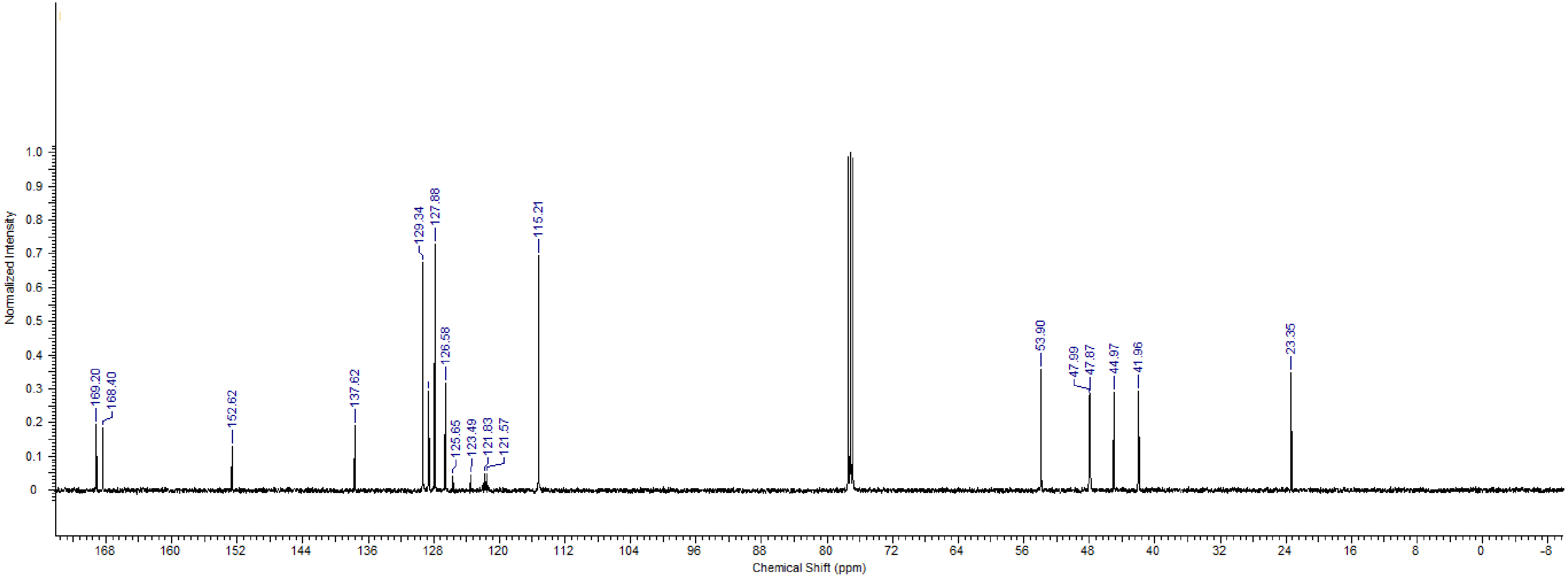

***N*-(2-(4-(3,5-bis(trifluoromethyl)phenyl)piperazin-1-yl)-2-oxo-1-phenylethyl)acetamide (55) –** ^{1}H NMR

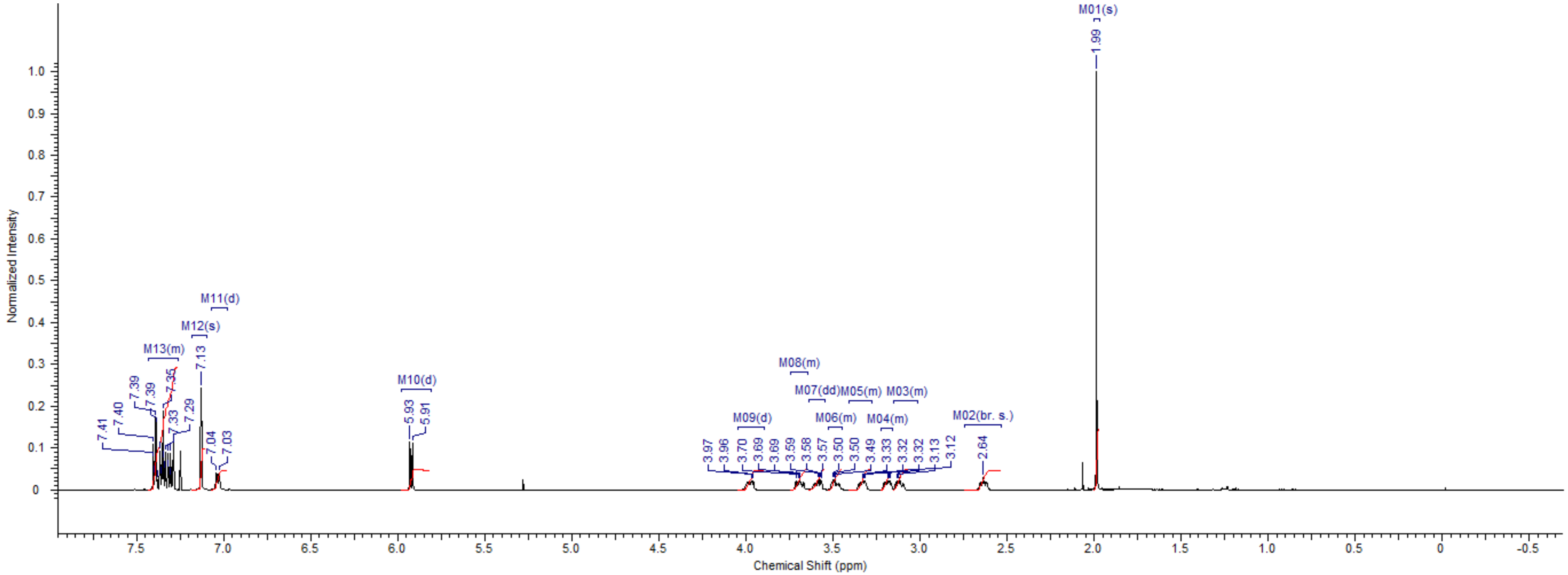

***N*-(2-(4-(3,5-bis(trifluoromethyl)phenyl)piperazin-1-yl)-2-oxo-1-phenylethyl)acetamide (55) –** ^{13}C NMR

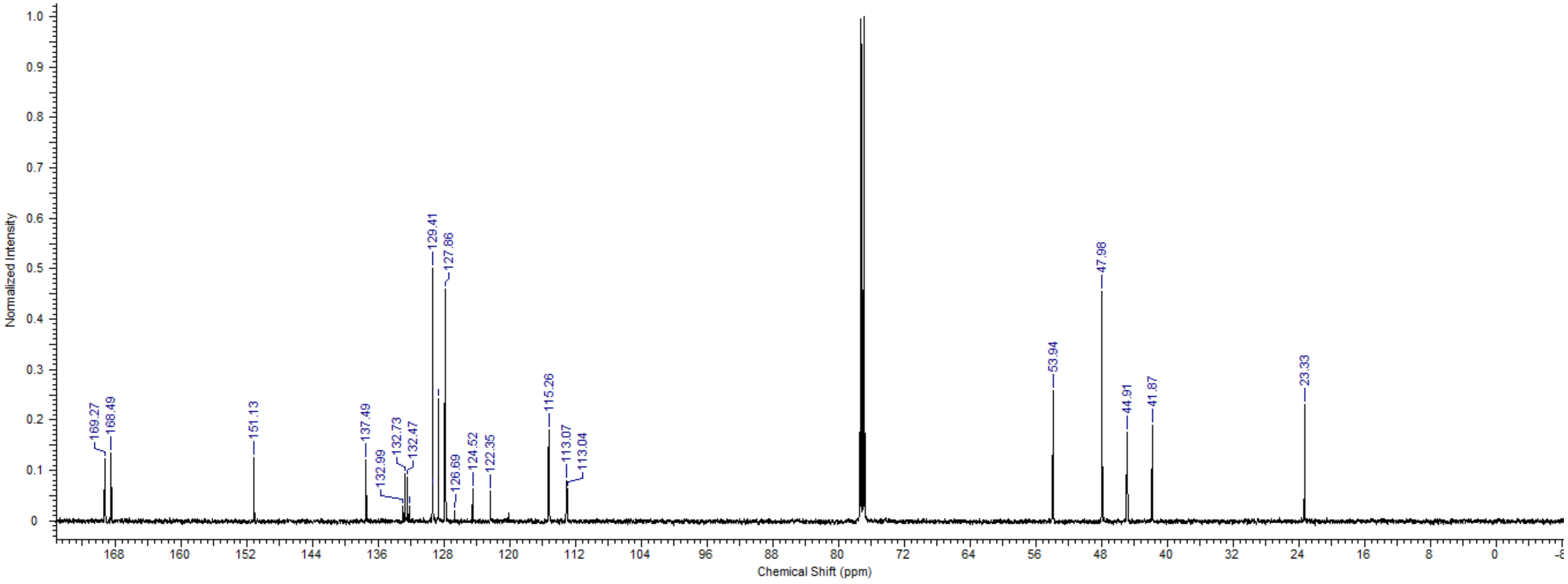

Supporting Information

***N*-(2-oxo-1-phenyl-2-(4-(m-tolyl)piperazin-1-yl)ethyl)acetamide (56) –** ^{1}H NMR

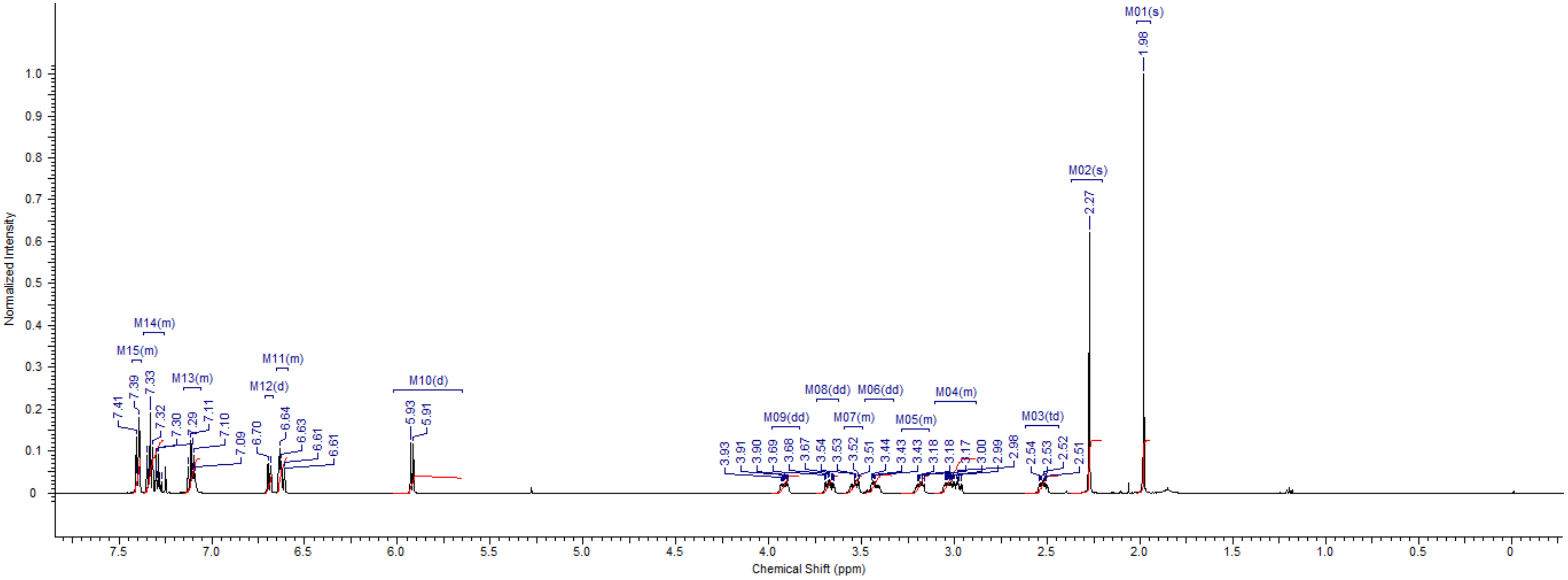

***N*-(2-oxo-1-phenyl-2-(4-(m-tolyl)piperazin-1-yl)ethyl)acetamide (56)** – ^{13}C NMR

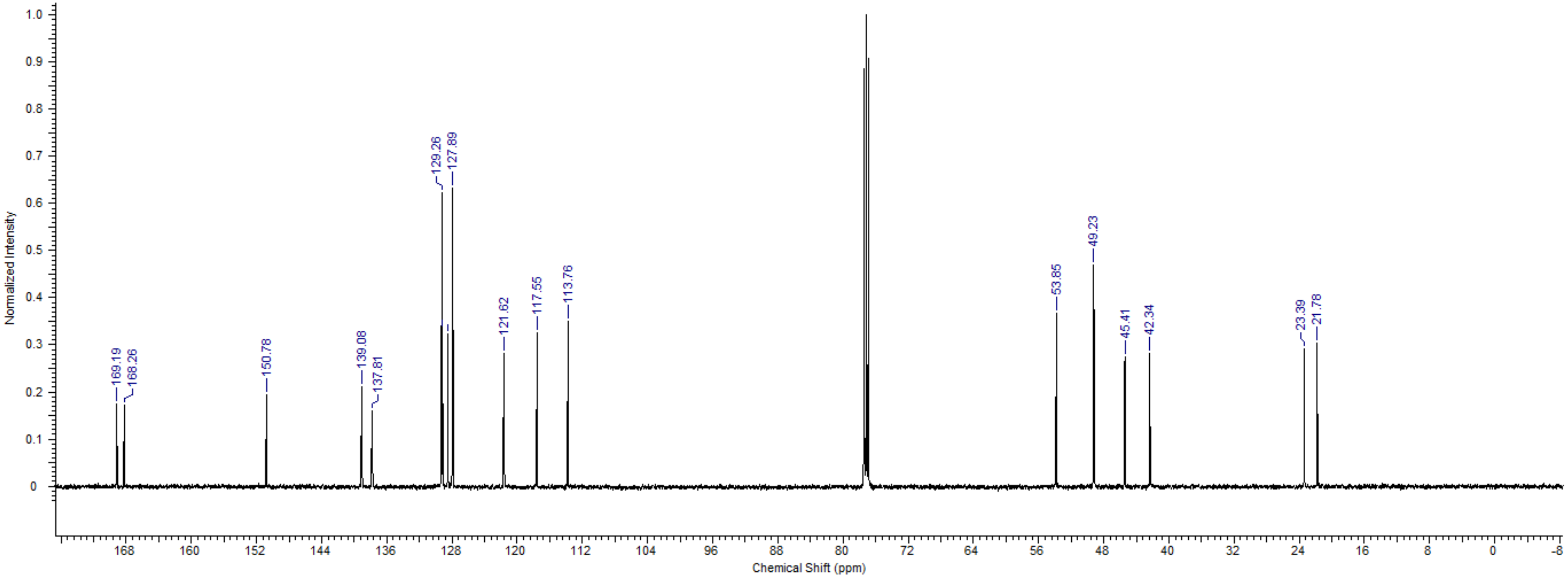

***N*-(2-(4-(3-(difluoromethyl)phenyl)piperazin-1-yl)-2-oxo-1-phenylethyl)acetamide (57)** – ^{1}H NMR

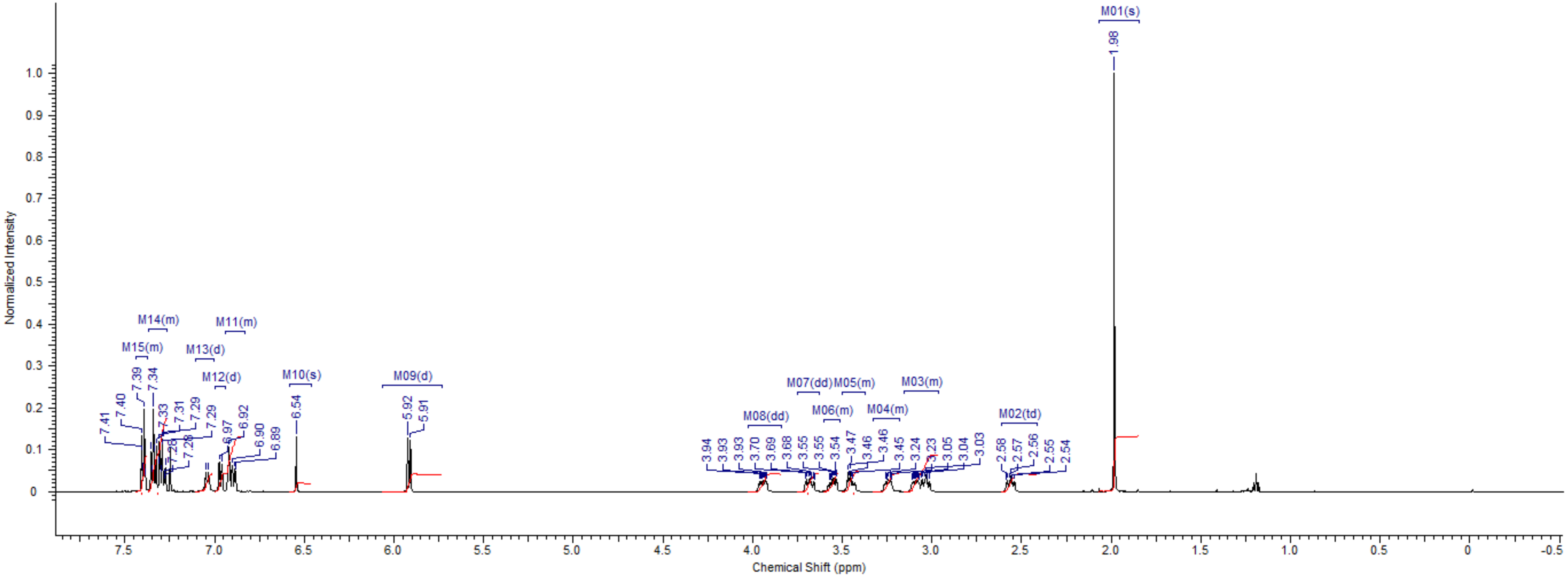

***N*-(2-(4-(3-(difluoromethyl)phenyl)piperazin-1-yl)-2-oxo-1-phenylethyl)acetamide (57) –** ^{13}C NMR

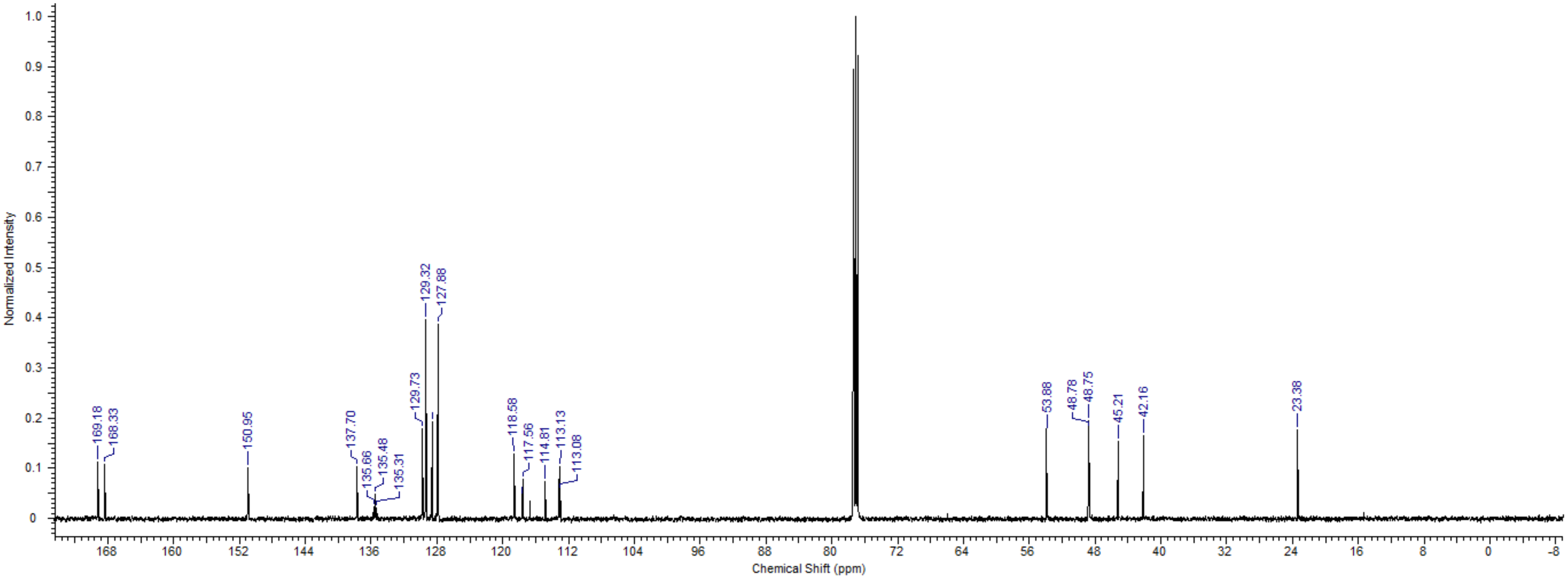

***N*-(2-(4-([1,1'-biphenyl]-3-yl)piperazin-1-yl)-2-oxo-1-phenylethyl)acetamide (58) –** ^{1}H NMR

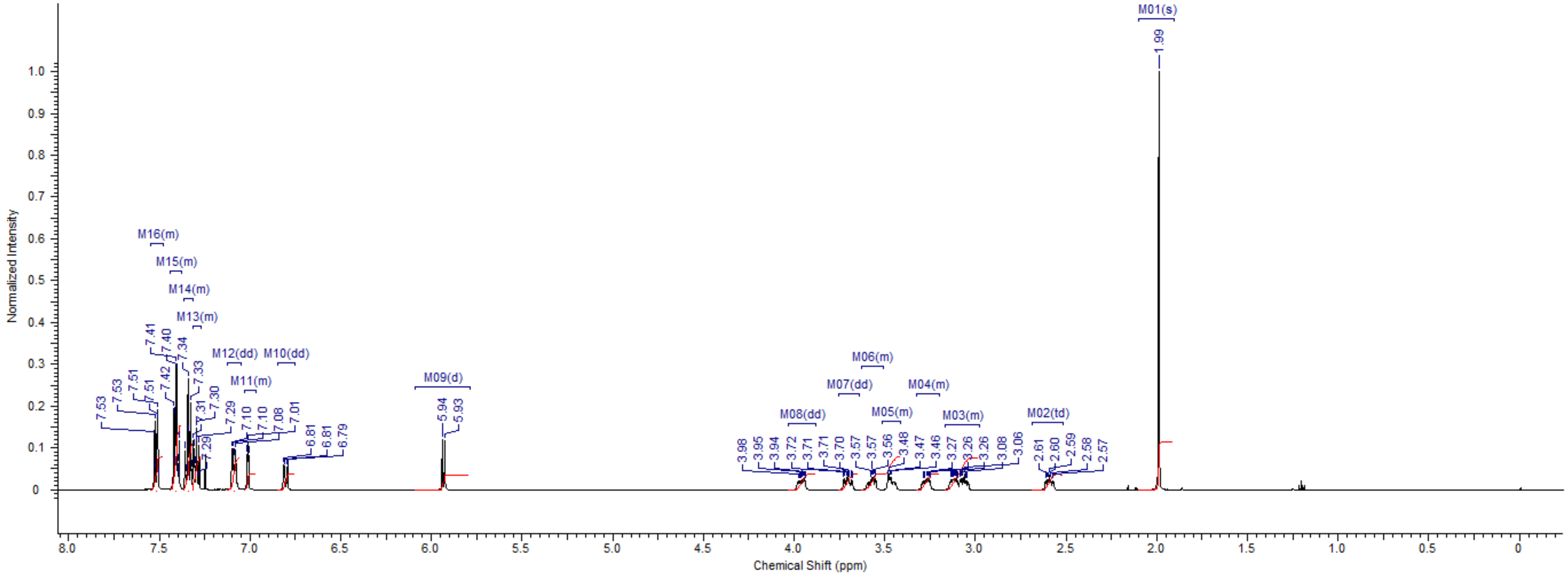

***N*-(2-(4-([1,1'-biphenyl]-3-yl)piperazin-1-yl)-2-oxo-1-phenylethyl)acetamide (58) –** ^{13}C NMR

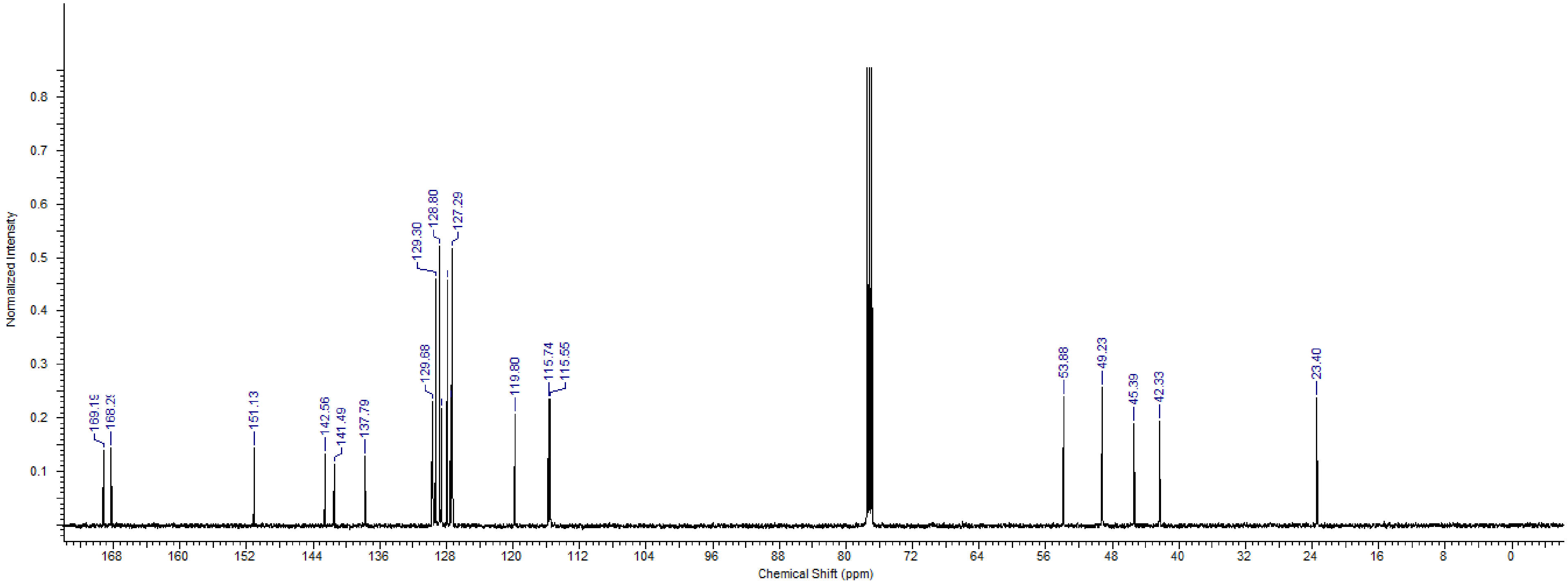

***N*-(2-(4-(3-methoxyphenyl)piperazin-1-yl)-2-oxo-1-phenylethyl)acetamide (59)** – ^{1}H NMR

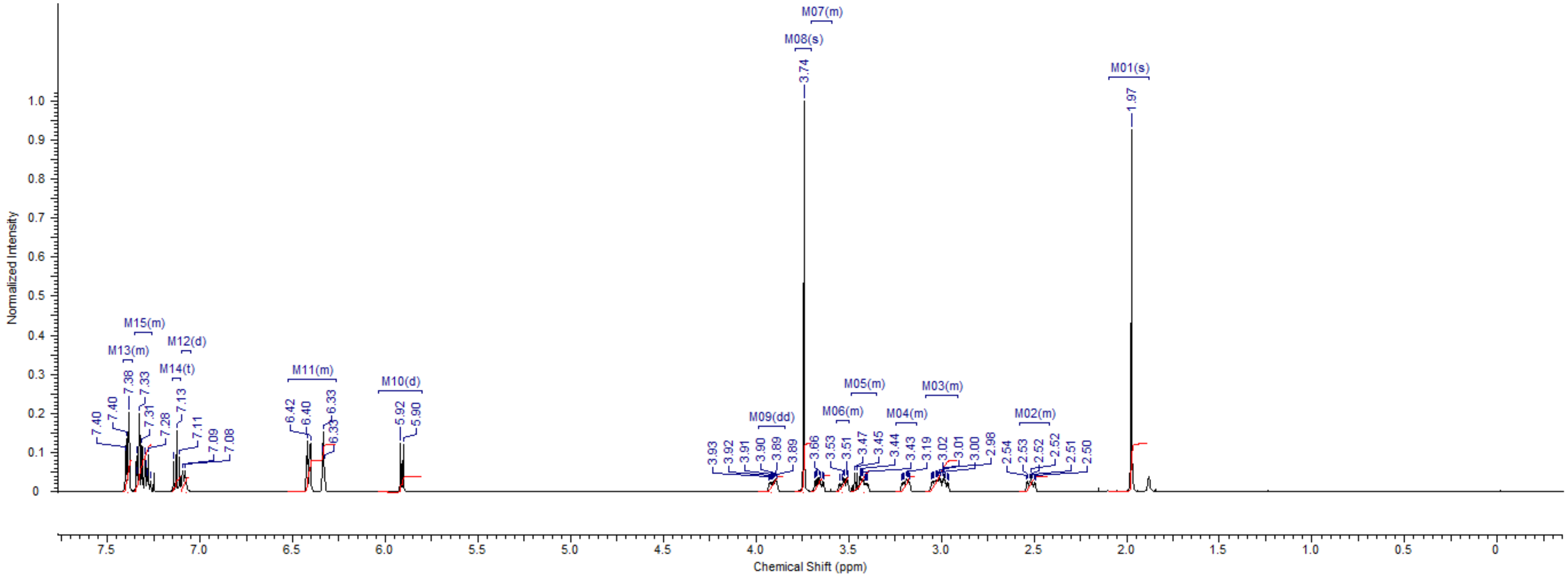

***N*-(2-(4-(3-methoxyphenyl)piperazin-1-yl)-2-oxo-1-phenylethyl)acetamide (59)** – ^{13}C NMR

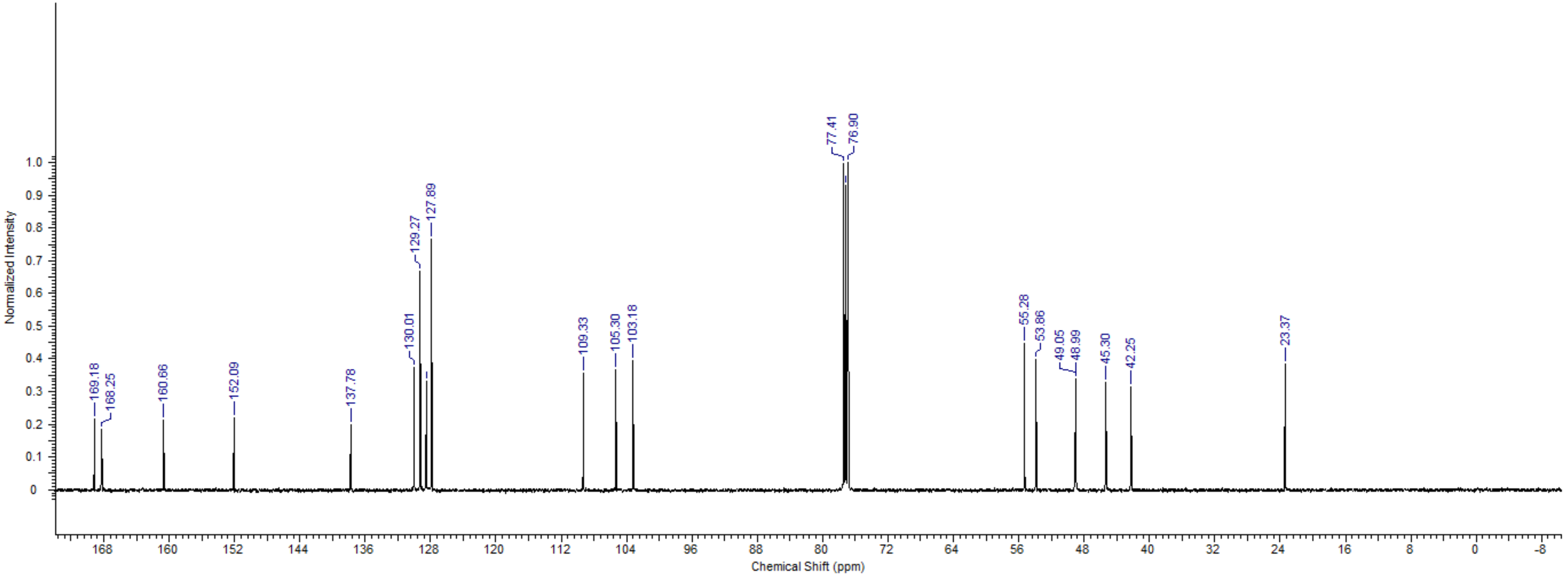

N-(2-oxo-1-phenyl-2-(4-(3-(trifluoromethoxy)phenyl)piperazin-1-yl)ethyl)acetamide (60) – ^{1}H NMR

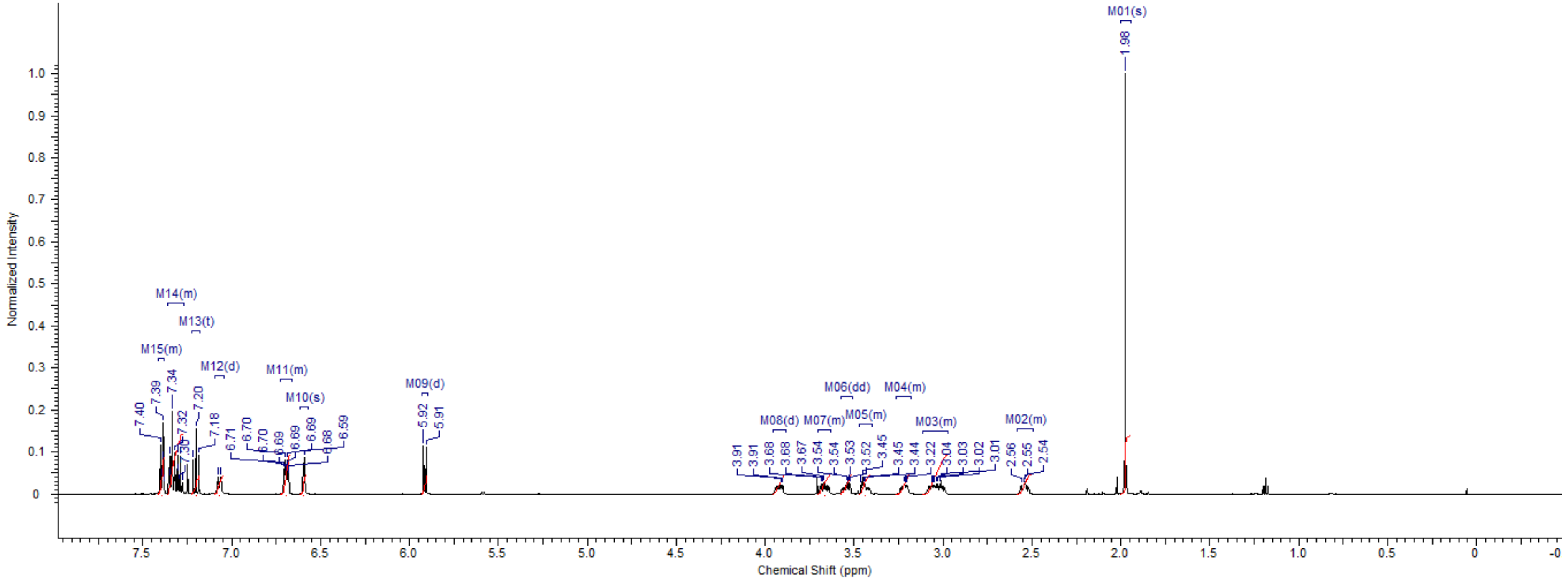

***N*-(2-oxo-1-phenyl-2-(4-(3-(trifluoromethoxy)phenyl)piperazin-1-yl)ethyl)acetamide (60) –** ^{13}C NMR

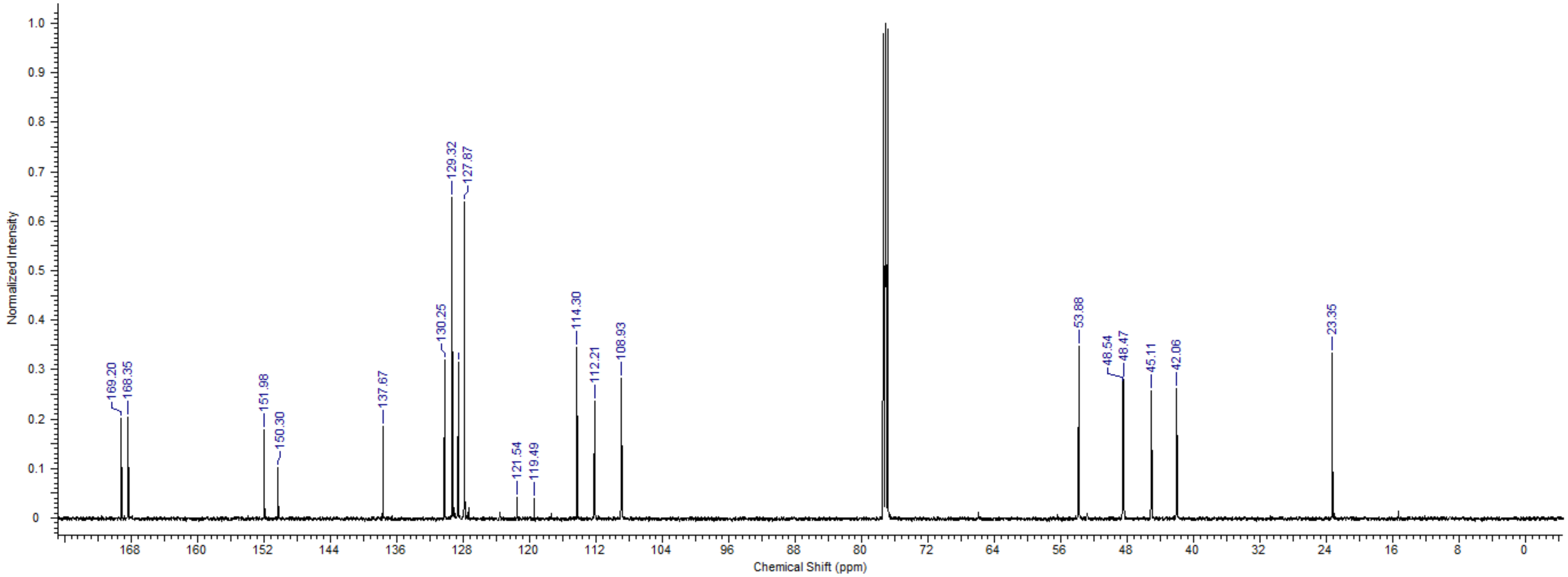

***N*-(2-oxo-2-(4-(3-phenoxyphenyl)piperazin-1-yl)-1-phenylethyl)acetamide (61) –** ^{1}H NMR

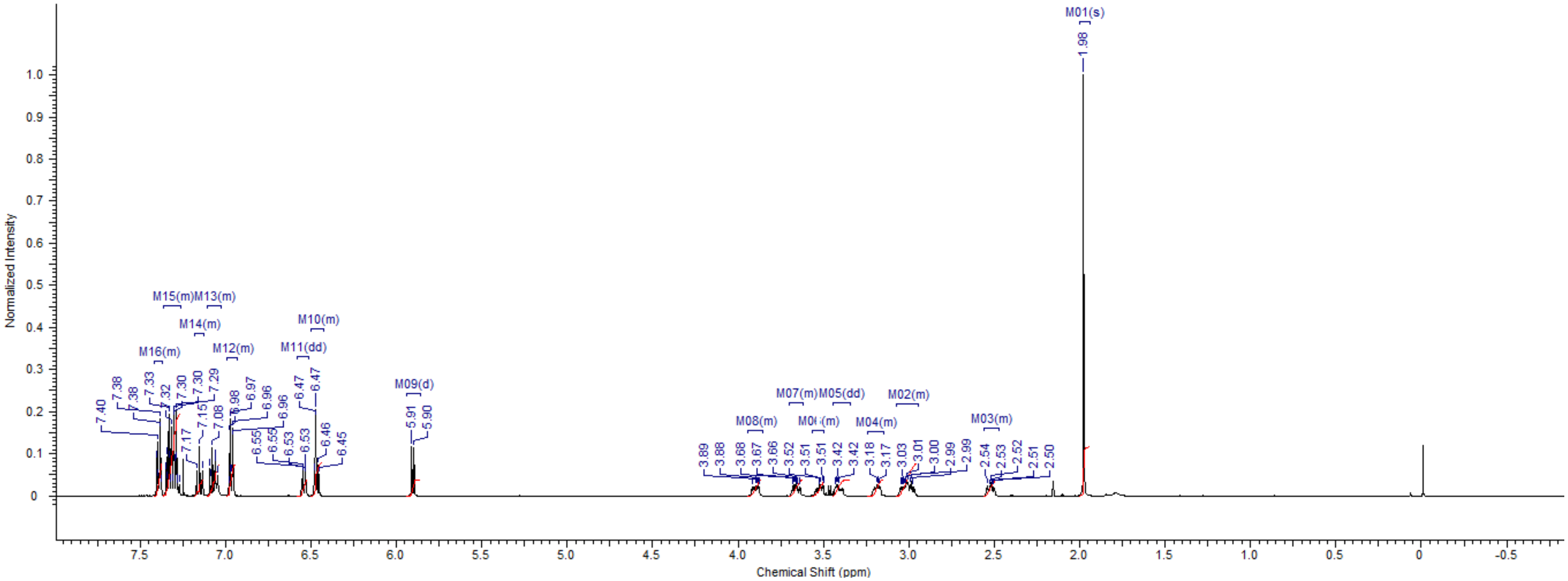

***N*-(2-oxo-2-(4-(3-phenoxyphenyl)piperazin-1-yl)-1-phenylethyl)acetamide (61) –** ^{13}C NMR

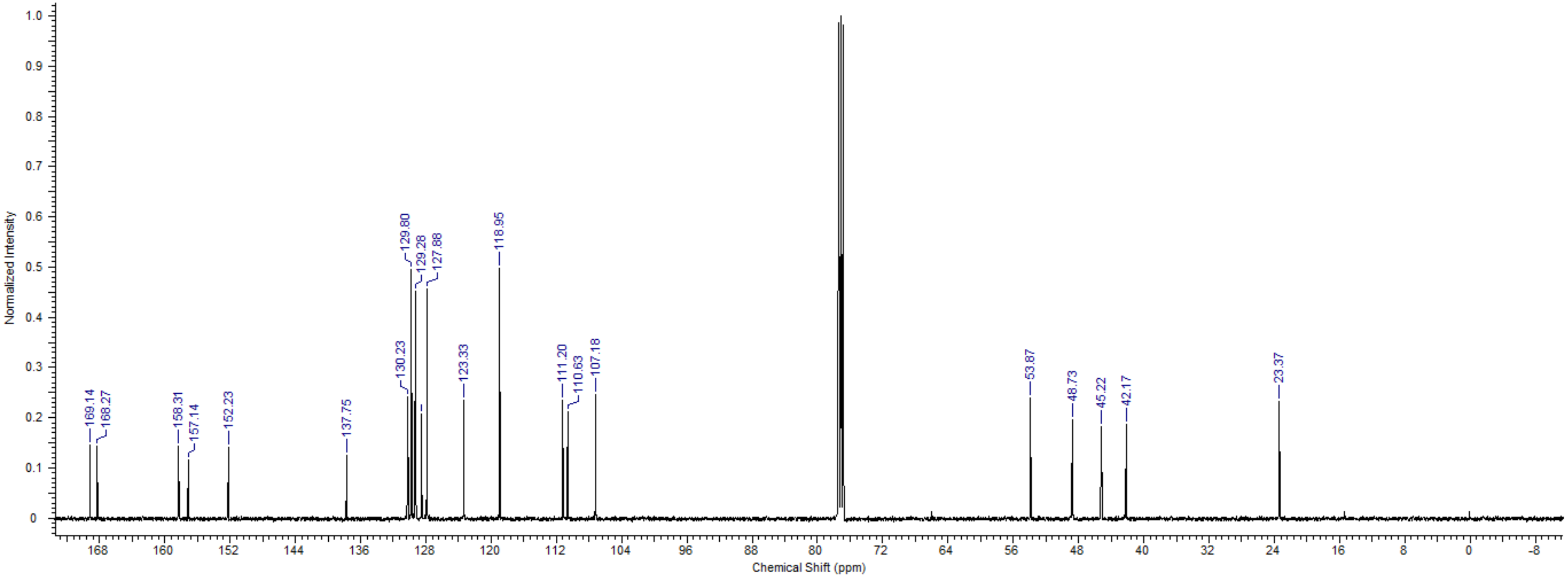

N-(2-oxo-1-phenyl-2-(4-(3-((trifluoromethyl)thio)phenyl)piperazin-1-yl)ethyl)acetamide (62) – ^{1}H NMR

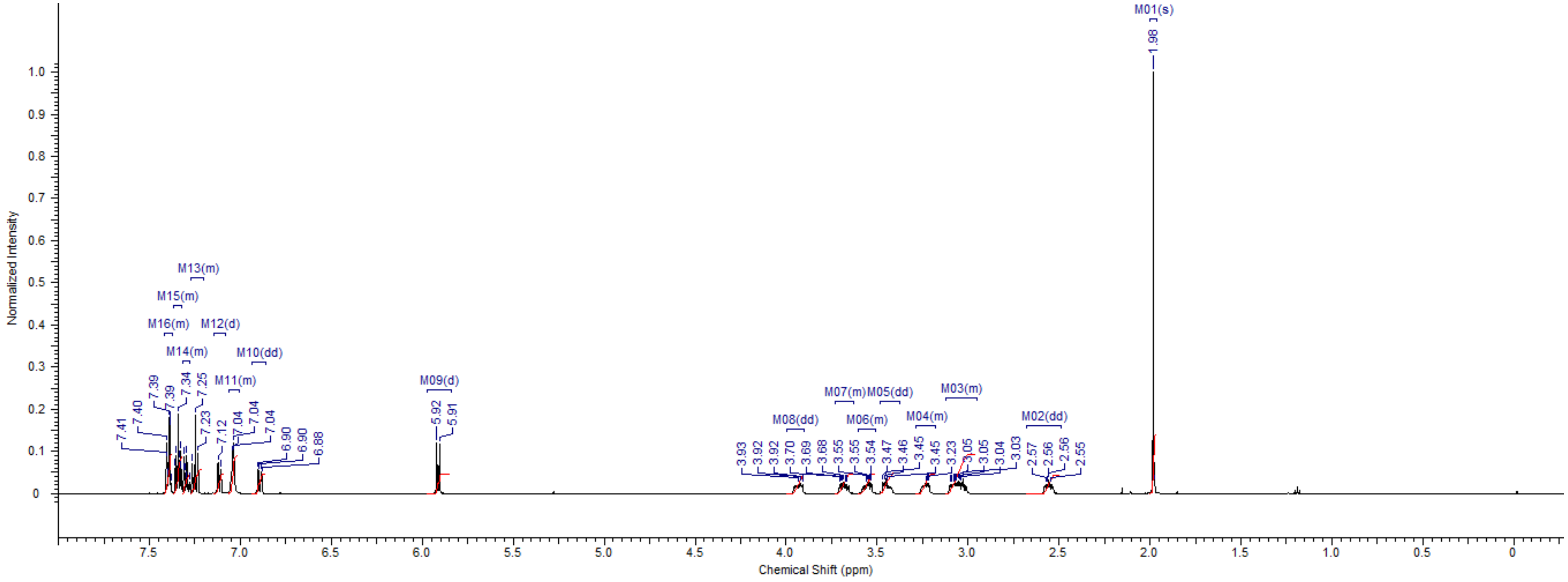

***N*-(2-oxo-1-phenyl-2-(4-(3-((trifluoromethyl)thio)phenyl)piperazin-1-yl)ethyl)acetamide (62) –** ^{13}C NMR

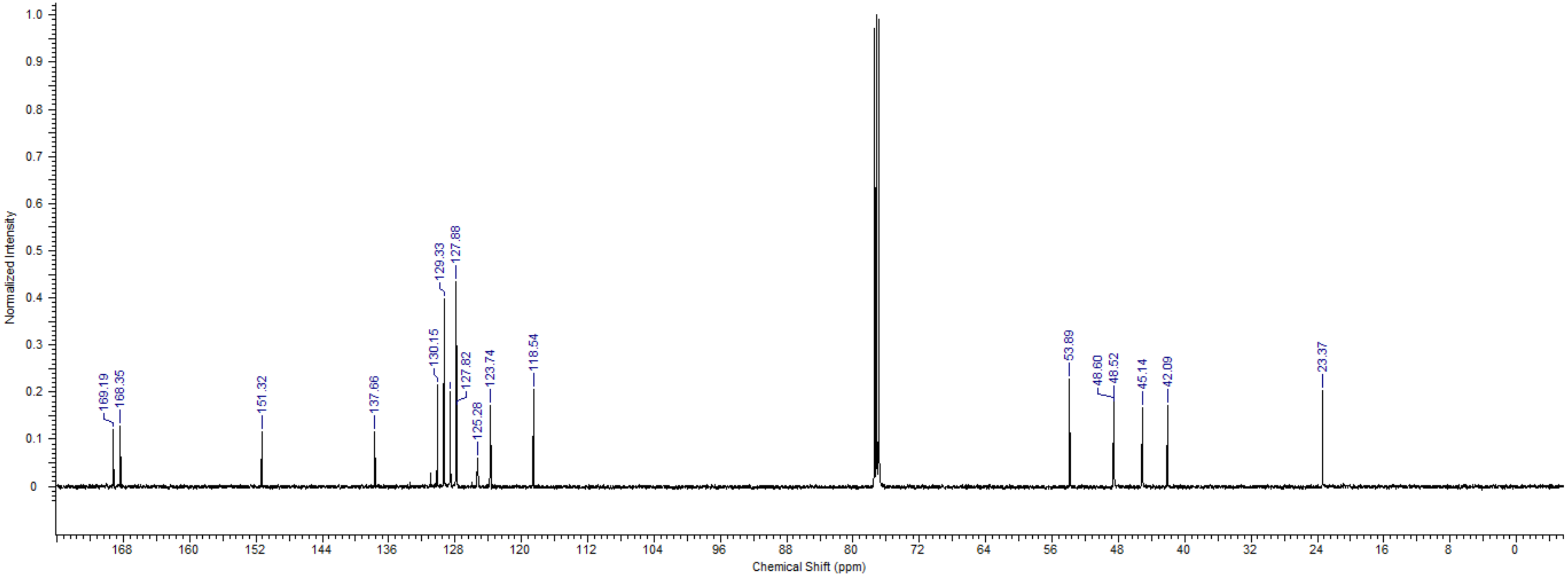

***N*-(2-oxo-1-phenyl-2-(4-(3-(trifluoromethyl)pyridin-2-yl)piperazin-1-yl)ethyl)acetamide (63) –** ^{1}H NMR

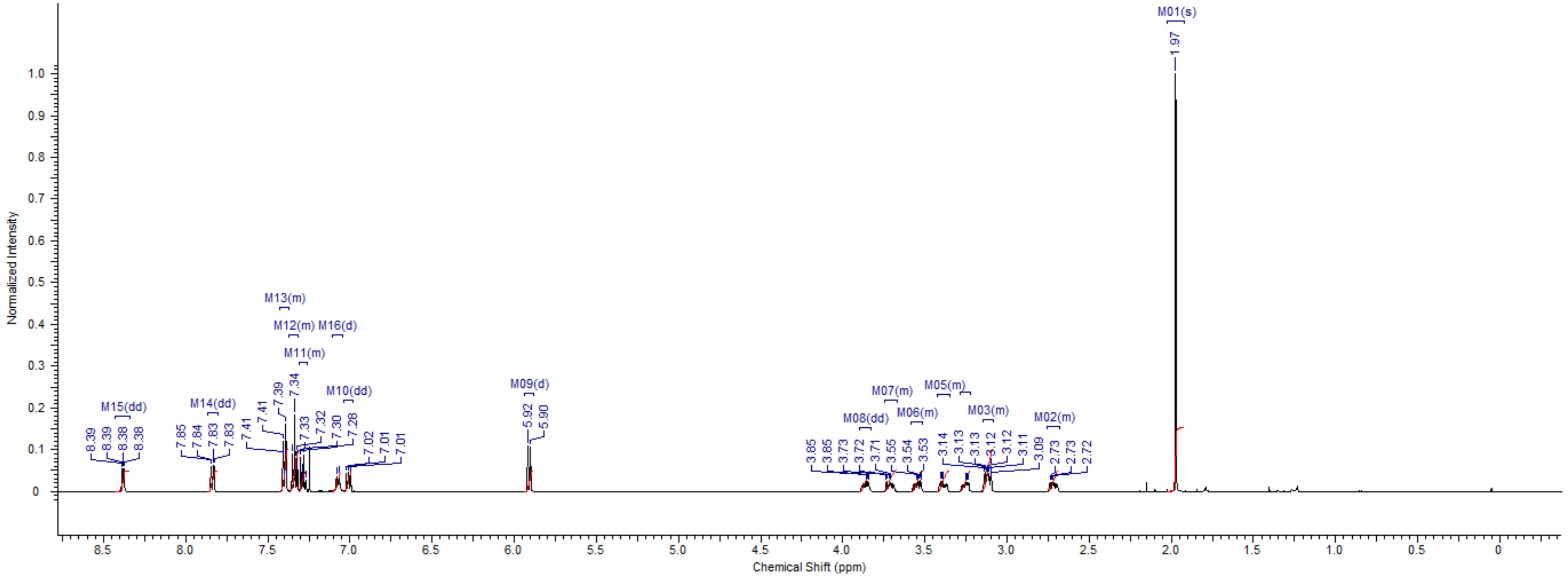

***N*-(2-oxo-1-phenyl-2-(4-(3-(trifluoromethyl)pyridin-2-yl)piperazin-1-yl)ethyl)acetamide (63) –** ^{13}C NMR

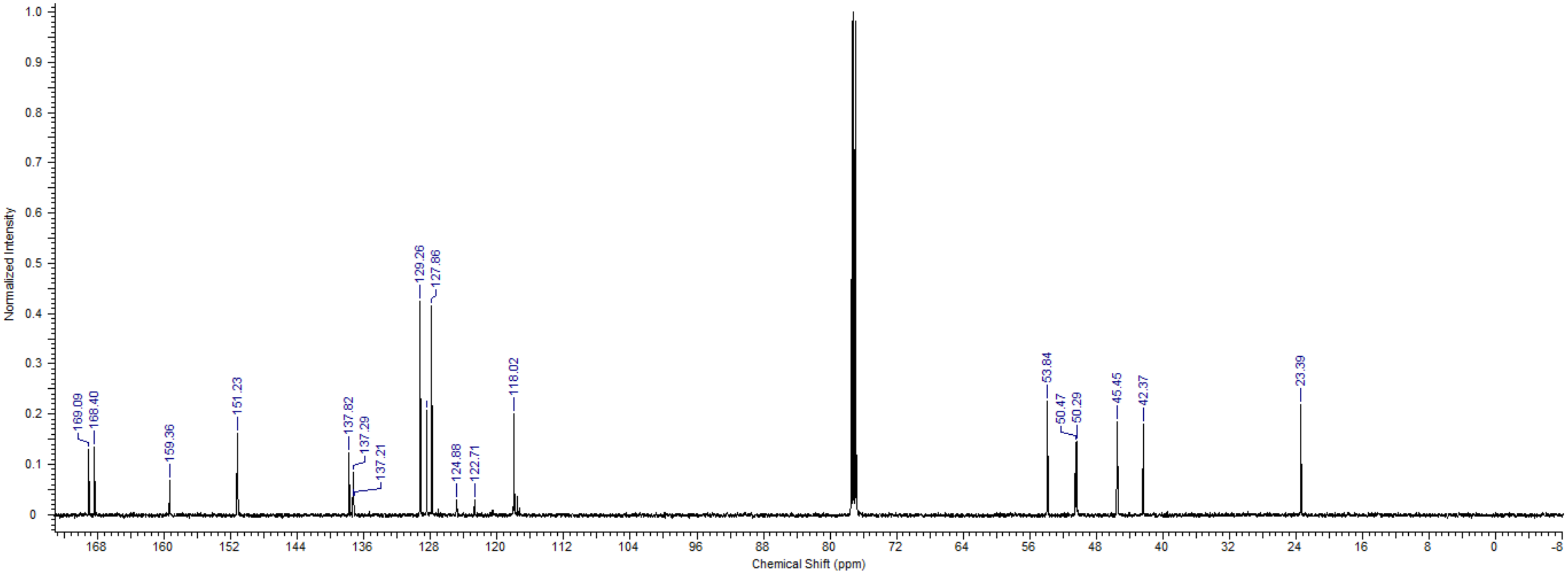

***N*-(2-oxo-1-phenyl-2-(4-(4-(trifluoromethyl)pyridin-2-yl)piperazin-1-yl)ethyl)acetamide (64)** – ^{1}H NMR

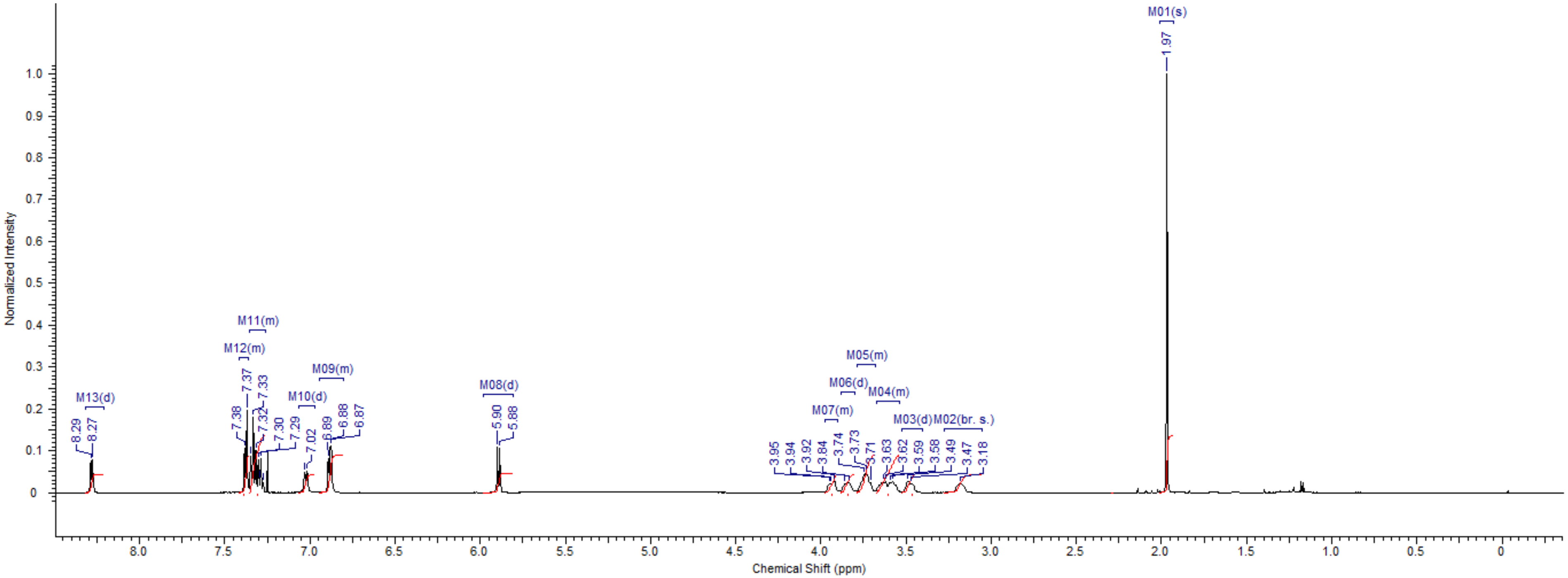

***N*-(2-oxo-1-phenyl-2-(4-(4-(trifluoromethyl)pyridin-2-yl)piperazin-1-yl)ethyl)acetamide (64) –** ^{13}C NMR

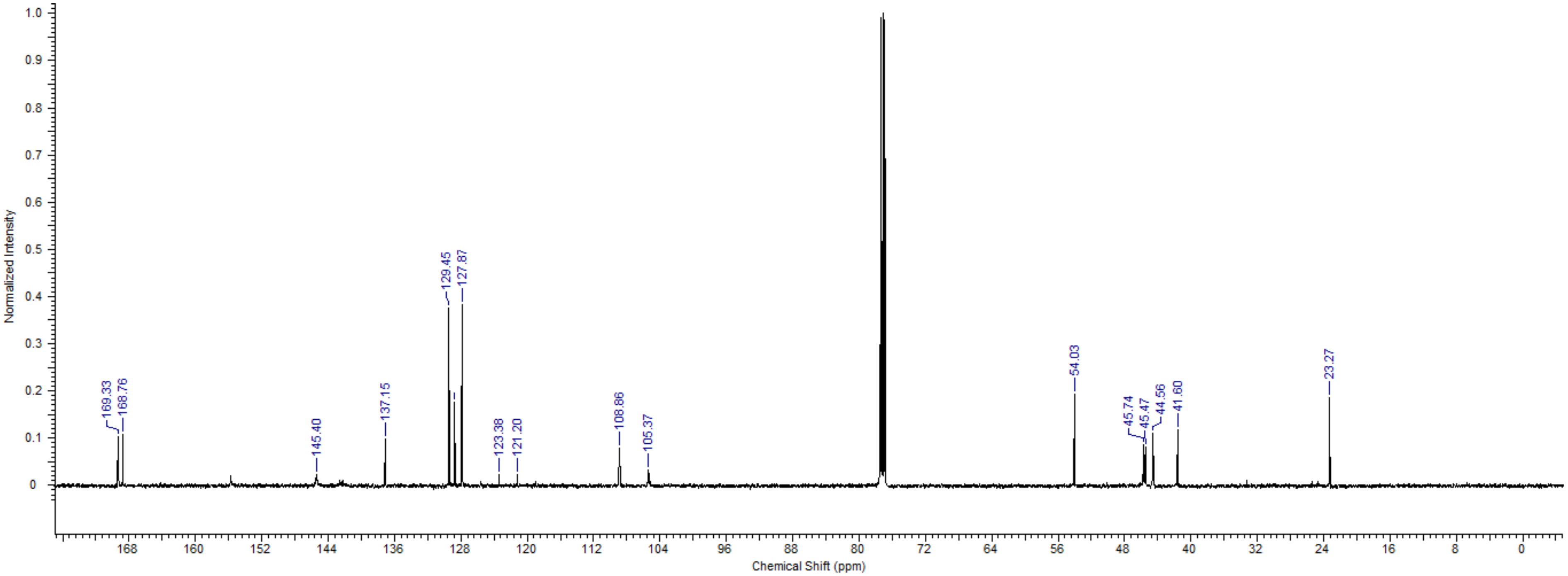

N-(2-oxo-1-phenyl-2-(4-(5-(trifluoromethyl)pyridin-2-yl)piperazin-1-yl)ethyl)acetamide (65) – ^{1}H NMR

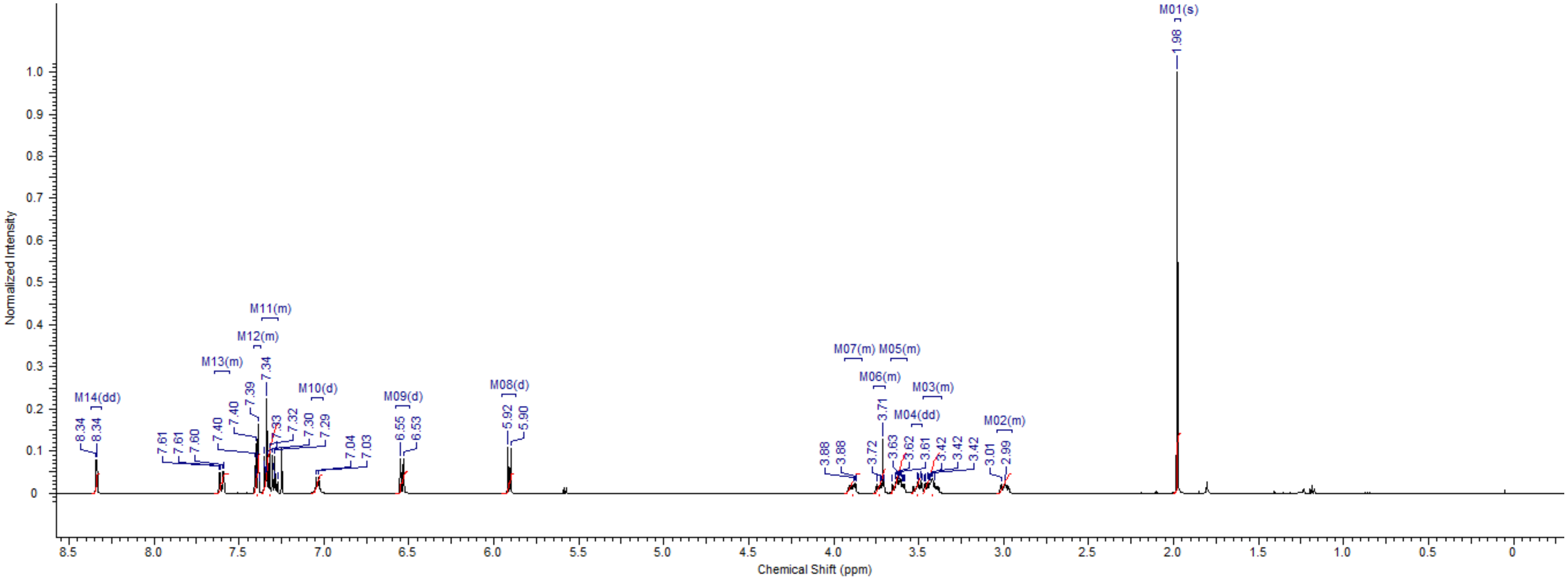

***N*-(2-oxo-1-phenyl-2-(4-(5-(trifluoromethyl)pyridin-2-yl)piperazin-1-yl)ethyl)acetamide (65) –** ^{13}C NMR

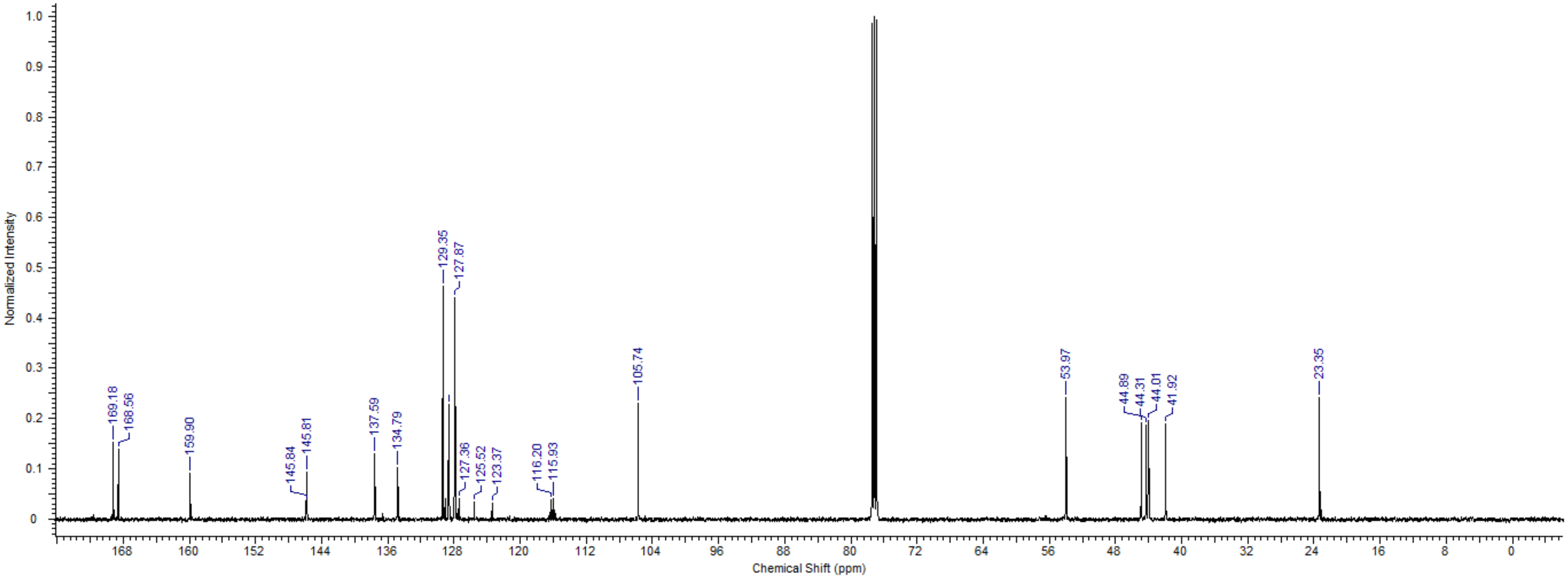

***N*-(2-oxo-1-phenyl-2-(4-(6-(trifluoromethyl)pyridin-2-yl)piperazin-1-yl)ethyl)acetamide (66) –** ^{1}H NMR

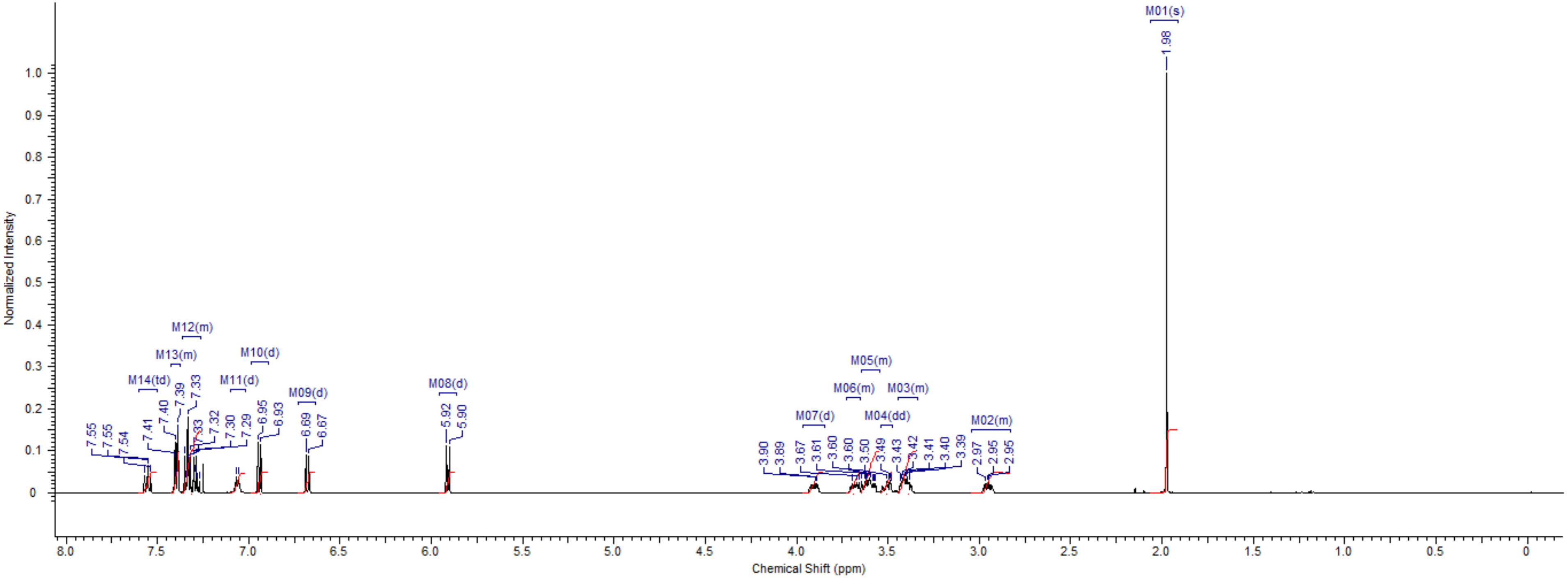

***N*-(2-oxo-1-phenyl-2-(4-(6-(trifluoromethyl)pyridin-2-yl)piperazin-1-yl)ethyl)acetamide (66) –** ^{13}C NMR

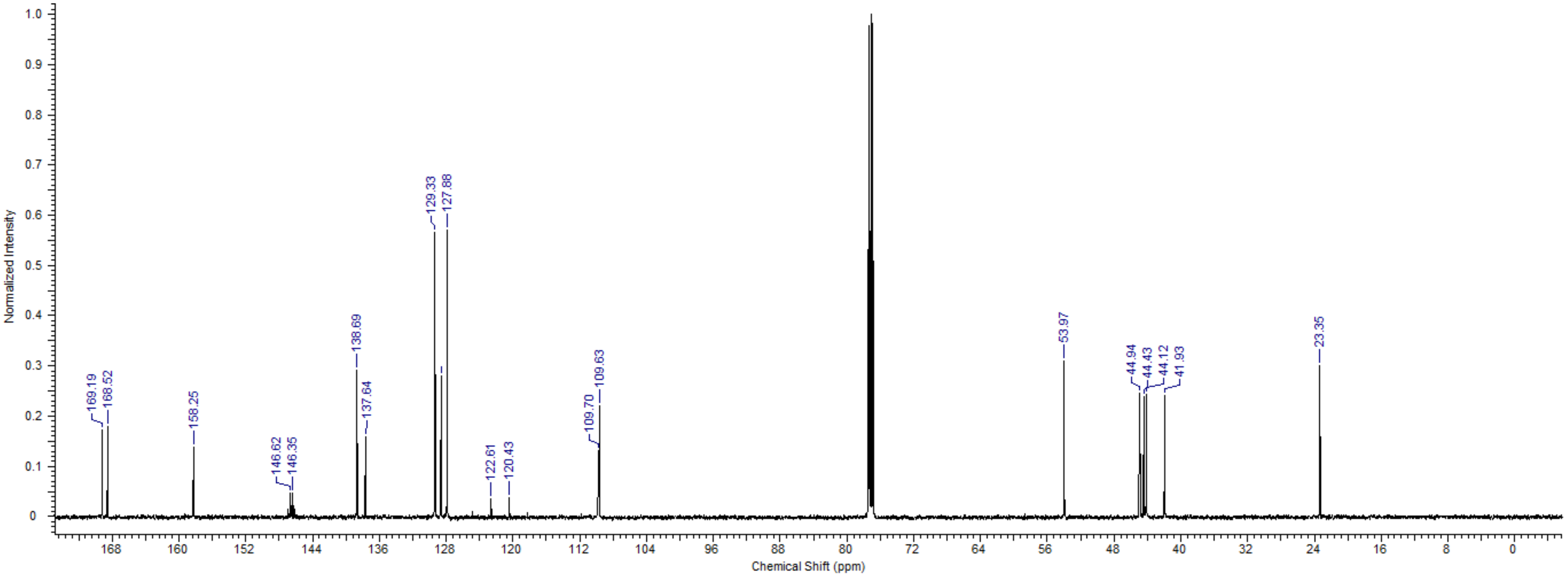

Supplement: Supplementary file 1 [file cells-11-01862-s001.zip › cells-1753974-supplementary.pdf]
